# Immunoregulation of follicular renewal, selection, POF, and menopause *in vivo*, vs. neo-oogenesis *in vitro*, POF and ovarian infertility treatment, and a clinical trial

**DOI:** 10.1186/1477-7827-10-97

**Published:** 2012-11-23

**Authors:** Antonin Bukovsky, Michael R Caudle

**Affiliations:** 1 The Institute of Biotechnology, Academy of Sciences of the Czech Republic, Prague, Czech Republic; 2 Cherokee Health Systems, 2018 Western Avenue, Knoxville, Tennessee, 37921, USA

**Keywords:** Fetal neo-oogenesis, Follicular renewal in mammals, Follicular selection, Granulosa cell renewal, Immune physiology, Neo-oogensis during the prime reproductive period, Neo-oogenesis *in vitro*, Ovarian function, Ovary, Tissue homeostasis

## Abstract

The immune system plays an important role in the regulation of tissue homeostasis ("tissue immune physiology"). Function of distinct tissues during adulthood, including the ovary, requires (1) Renewal from stem cells, (2) Preservation of tissue-specific cells in a proper differentiated state, which differs among distinct tissues, and (3) Regulation of tissue quantity. Such morphostasis can be executed by the tissue control system, consisting of immune system-related components, vascular pericytes, and autonomic innervation. Morphostasis is established epigenetically, during morphogenetic (developmental) immune adaptation, i.e., during the critical developmental period. Subsequently, the tissues are maintained in a state of differentiation reached during the adaptation by a “stop effect” of resident and self renewing monocyte-derived cells. The later normal tissue is programmed to emerge (e.g., late emergence of ovarian granulosa cells), the earlier its function ceases. Alteration of certain tissue differentiation during the critical developmental period causes persistent alteration of that tissue function, including premature ovarian failure (POF) and primary amenorrhea. In fetal and adult human ovaries the ovarian surface epithelium cells called ovarian stem cells (OSC) are bipotent stem cells for the formation of ovarian germ and granulosa cells. Recently termed oogonial stem cells are, in reality, not stem but already germ cells which have the ability to divide. Immune system-related cells and molecules accompany asymmetric division of OSC resulting in the emergence of secondary germ cells, symmetric division, and migration of secondary germ cells, formation of new granulosa cells and fetal and adult primordial follicles (follicular renewal), and selection and growth of primary/preantral, and dominant follicles. The number of selected follicles during each ovarian cycle is determined by autonomic innervation. Morphostasis is altered with advancing age, due to degenerative changes of the immune system. This causes cessation of oocyte and follicular renewal at 38 +/-2 years of age due to the lack of formation of new granulosa cells. Oocytes in primordial follicles persisting after the end of the prime reproductive period accumulate genetic alterations resulting in an exponentially growing incidence of fetal trisomies and other genetic abnormalities with advanced maternal age. The secondary germ cells also develop in the OSC cultures derived from POF and aging ovaries. *In vitro* conditions are free of immune mechanisms, which prevent neo-oogenesis *in vivo*. Such germ cells are capable of differentiating *in vitro* into functional oocytes. This may provide fresh oocytes and genetically related children to women lacking the ability to produce their own follicular oocytes. Further study of "immune physiology" may help us to better understand ovarian physiology and pathology, including ovarian infertility caused by POF or by a lack of ovarian follicles with functional oocytes in aging ovaries. The observations indicating involvement of immunoregulation in physiological neo-oogenesis and follicular renewal from OSC during the fetal and prime reproductive periods are reviewed as well as immune system and age-independent neo-oogenesis and oocyte maturation in OSC cultures, perimenopausal alteration of homeostasis causing disorders of many tissues, and the first OSC culture clinical trial.

## Table of contents

1. Background

1.1. A concept for the immune system role in the regulation of ovarian function

1.2. Developmental immune adaptation and development of immune tolerance

2. Immune system and tissue homeostasis (tissue immune physiology)

2.1. The tissue control system theory

3. Immune system and *in vivo* regulation of ovarian function

3.1. Comparison of oocyte "storage" and "continued formation" theories

3.1.1. The prime reproductive period theory

3.2. A reversal of the oocyte storage to the continued oocyte formation theory and new perspectives in the treatment of POF and ovarian infertility caused by a lack of ovarian follicles with functional oocytes

3.3. Primordial germ cells

4. Human embryonic and fetal ovaries - mechanisms of oocyte formation

4.1. Human embryonic ovaries

4.2. Human fetal ovaries

4.2.1. Origin of secondary germ cells and granulosa cells from fetal ovarian stem cells

4.2.2. Rete ovarii channels contain immune system-related cells

4.2.3. Degeneration of fetal oocytes

4.2.4. Origin of primitive granulosa cells

4.2.5. Secondary germ cells originate from asymmetric division of ovarian stem cells

4.2.6. Monocyte-derived cells and T cells accompany origin of secondary germ cells

4.2.7. Conclusions on the origin of secondary germ cells

5. Cessation of oogenesis in prenatal human ovaries

6. Oocyte and follicular renewal in humans during the prime reproductive period

6.1. Origin of new granulosa and germ cells from bipotent ovarian stem cells

6.1.1. Origin of new granulosa cells

6.1.2. Origin of new germ cells

6.2. Involvement of the immune system-related cells

6.3. Localization of SCP3 in adult human and monkey ovaries

6.4. Summary on oocyte and follicular renewal in adult human ovaries

7. Developmental immune adaptation and determination of the aging of the ovary and other tissues

7.1. Thymus and reproduction

7.2. The working hypothesis

7.3. Premature failure of ovaries with primordial follicles and animal models

7.4. The tissue control system theory and a "stop-effect" of monocyte-derived cells

7.5. The immune system memory and aging of the body

8. Former and current views on ovarian oogenesis and follicular renewal

8.1. Milestones of the oocyte storage theory

8.2. Oogenesis in adult prosimians

8.3. Rodent ovaries

8.3.1. Functional repair of anovulatory mouse ovaries with cultured germline stem cells

8.4. Summary on the current views

9. Follicular Selection

9.1. Selection of growing (secondary) follicles

9.2. Selection of a dominant follicle

9.3. Novel aspects of follicular selection

9.4. Follicular atresia

10. Developmental potential of ovarian stem cells *in vitro*

10.1. Cell types developing from omnipotent ovarian stem cells

10.2. Culture conditions and techniques

10.3. Estrogens are essential for the neo-oogenesis in vitro

10.4. Development of oocytes and parthenogenetic embryos in vitro

10.4.1. Primary ovarian stem cell cultures

10.4.2. Secondary ovarian stem cell cultures

10.4.3. Ovarian stem cell cultures vs. in vivo oocyte and follicular development in mammals

10.4.4. A comparison of the primary vs. secondary ovarian stem cell cultures

10.5. Development of embryonic stem cells from in vitro developed parthenotes

10.6. Development of oocytes from postmenopausal and POF ovaries

10.7. Cultures from ovaries lacking ovarian stem cells fail to produce oocytes

11. Oocyte formation by mitotically active germ cells purified from ovaries of reproductive-age women

11.1. Repowering the ovary

11.2. The importance of the presence of uncommitted granulosa cell nests for the preservation and development of transplanted primitive germ cells

11.3. A lack of uncommitted granulosa cell nests causes a degeneration of the germ cells

11.4. Alternative approaches for the treatment of ovarian infertility

11.5. Restoration of the ovarian stem cell niche after chemotherapy

12. Neo-oogenesis *in vitro* vs. conventional IVF

13. Why does menopause occur?

13.1. A physiological role of ovarian stem cells in normal ovaries

13.2. Availability of granulosa cells

13.3. Why ovarian stem cells do not prevent a menopause?

13.4. Perimenopausal disorders

14. Clinical trial

14.1. Differentiation of oocytes from ovarian stem cells in vitro

14.2. Potential treatment of ovarian infertility

14.3. Suitability of patients for clinical trial

14.4. Collection of ovarian stem cells and in vitro culture of oocytes

14.5. Potential pitfalls

14.6. Initiation of the first clinical trial

15. Conclusions

16. References

## 1. Background

Available data indicate that the function of distinct tissues, including the ovary, is regulated by so-called morphostasis. Morphostasis is a complex event requiring: 1) Renewal from stem cells, 2) Preservation of tissue-specific cells in a proper differentiated state, and 3) Regulation of tissue quantity. This can be executed by the tissue control system (TCS) consisting of immune system-related components, vascular pericytes, and autonomic innervation. Morphostasis is established epigenetically, during morphogenetic developmental immune adaptation, i.e., during the critical developmental period in embryonic and fetal life. Subsequently, the tissues are maintained in a state of differentiation reached during the adaptation by a “stop effect” of the resident monocyte-derived cells (MDC). Alteration of tissue differentiation during the critical developmental period causes persistent alteration of tissue function. Morphostasis is altered with age advancement, due to degenerative changes of the immune system. Thus ovarian function ceases and an increased incidence of neoplasia and degenerative diseases occurs with aging (reviewed in [1]).

In this article, the term primordial germ cells is used to designate the extragonadal germ cells migrating into gonads during the early embryonic period. The term secondary, or just germ cells, designates cells originating from the ovarian stem cells (OSC) *in vivo*. Ovarian stem cells are epithelial cells covering ovaries, and were originally called “ovarian germinal epithelium” [2,3], then ovarian surface epithelium [4-6], and now ovarian stem cells [7-10]. They are considered to be epithelial in nature, but could originate by mesenchymal-epithelial conversion from the mesenchymal precursors in ovarian tunica albuginea (TA) of adult individuals in some species, such as humans [11,12].

The primordial follicles can be divided into "fetal primordial follicles," formed during the second trimester of intrauterine life from secondary germ cells and emerging granulosa cells, and persisting until the perimenarcheal period, and "adult primordial follicles," formed by follicular renewal during the prime reproductive period (from menarche till 38 +/-2 years of age) and persisting thereafter until menopause.

### 1.1. A concept for the immune system role in the regulation of ovarian function

Until late 1970', regulation of ovarian function was considered limited to interactions between the hypothalamo-pituitary system and the ovary. Such concepts, however, did not explain a number of experimental data in rats and mice, such as ovarian "dysgenesis" after neonatal thymectomy [13-16], prevention of steroid-induced sterility in neonatal rats by thymocytes from fertile females [17], and superovulation after cyclophosphamide [18] and x-ray treatment [19].

Our observations indicated that intraperitoneal treatment of adult rats with antithymocyte serum causes anovulation with the persistence of corpora lutea (CL) and a persisting diestrus [20]. We also found a migration of immune system-related cells among granulosa cells of atretic rat follicles [21,22], and a delayed ovarian maturation and shortened reproductive period in nude mice with congenital absence of the thymus [23].

In 1979, we were the first who proposed a role for the immune system in ovarian function [24], as recently noted by Joy Pate et al. [25]. We proposed that ovarian structures present during the fetal adaptive period of immune system development, such as primordial follicles, are tolerated by the immune system during adulthood, while those structures which were absent, such as antral follicles and CL, have a limited functional life unless pregnancy occurs. Under normal conditions, the antral follicles and CL should be cyclically destroyed in order to maintain the cyclic character of ovarian function. A lack of atresia of aged antral follicles results in their persistence and the polycystic ovary syndrome. In species with cyclic ovarian function, the length of the ovarian cycle (about four days in the rats and mice and 28 days in humans) is determined by the length of the immune cycle. We also proposed that age-dependent impairment of ovarian function is caused by the age-dependent impairment of the immune system [24]. In reality, the impairment of immune system function with age is the first in the body, and that of the ovary follows.

In 1980, Espey hypothesized that ovulation of ovarian follicles involves an inflammatory reaction [26]. The connective tissue layers of the TA and theca externa must be weakened to allow the follicle wall to dissociate and break open under a modest intrafollicular pressure. Such changes are probably caused by thecal fibroblasts transformed into proliferating cells similar to tissue responses to inflammatory reactions [27].

### 1.2. Developmental immune adaptation and development of immune tolerance

Developmental immune adaptation represents a stage of immune system development during which antigens which are present are tolerated after system immunocompetence, and those which are absent underwent immune rejection. In humans, the developmental immune adaptation is terminated after the second trimester and in small laboratory rodents, it ends after the 7th day of postnatal life [28].

## 2. Immune system and tissue homeostasis (tissue immune physiology)

During the last three decades, the participation of the immune system in regulation of proliferation, differentiation, and aging of tissue-specific cells in various epithelial, parenchymal, and muscle tissues has gained increasing interest [1,29-42].

Ninety years ago, Alexis Carrel demonstrated that, similarly to embryonic tissue extracts, leukocyte extracts also stimulate multiplication of fibroblasts *in vitro*, and suggested that leukocytes bring growth-activating substances to tissue-specific cells [43]. Later in the 1960s and 1970s, lymphocytes were shown to promote tissue growth and regeneration (reviewed in [44]). It has been suggested that participation in host immune responses is only one of the many functions of lymphocytes, since lymphoid cells also participate in a number of physiological processes of homeostasis [44]. While a lot of work has been done on the role of various growth factors and cytokines produced by mesenchymal cells on the cell cycle and death *in vitro*[45-58], still little is known about the interactions between mesenchymal and tissue-specific cells *in vivo*. The role of immune system components in the regulation of tissue physiology and pathology should also be viewed more widely, along with resident mesenchymal cells, such as fibroblast-derived vascular pericytes, and autonomic neural signals.

### 2.1. The tissue control system theory

Our studies in the late 1970s [23,24,59,60] and 1980s [61,62] resulted in the proposal of a wider role of the immune system (immune cells and vascular pericytes), the so-called tissue control system (TCS), in regulation of ovarian function [29]. The TCS theory was further refined when the role of autonomic innervation in the regulation of "quantitative aspects in tissues," including follicular selection, was added [63,64], and the TCS theory was revised [65,66]. More recently, a role for the immune system in the regulation of ovarian aging and the regulation of asymmetric cell division have been described [37,67,68]. In addition, we proposed a role of the TCS in immune maintenance of self related to tissue morphostasis, tumor growth, and regenerative medicine - reviewed in [1,69-72].

## 3. Immune system and *in vivo* regulation of ovarian function

Ovarian compartments are among those structures showing the most pronounced morphological (cellular proliferation, differentiation, and regression) and functional changes in the body. Regulation of ovarian function is complex, involving interactions between follicular compartments (oocyte, granulosa, and theca cells), as well as of sex steroids produced by follicles, CL, and interstitial tissues originating from theca of degenerating follicles. Additionally, communication of the hypothalamo-pituitary system and the influence of gonadotropins, autonomic innervation, growth factors, and cytokines produced by mesenchymal cells of the immune system all regulate functions of ovarian compartments. While gonadotropins are essential for follicular maturation and ovulation [73], autonomic innervation is necessary for regulation of follicular selection [74,75]. Interactions between the immune system and ovary are numerous, as immune cells are associated with regulation at every level of the hypothalamo-pituitary-ovarian axis, regulating growth and regression of both follicles and CL [76,77].

Ovulatory ovarian function during adulthood requires the presence of oocytes, the origin of which in higher vertebrates remains uncertain.

### 3.1. Comparison of the oocyte "storage" and "continued formation" theories

The origin of germ cells in adult females of higher vertebrates (birds and mammals) has been a matter of dispute more than one hundred years. There are, in principle, two views: the oocyte "storage" and "continued formation" theories [78].

The "storage" doctrine is based on the belief that there is never any increase in the number of oocytes beyond those differentiating during fetal or perinatal ovarian development from embryonic (primordial) germ cells [79]. This prevailing dogma was enhanced by the assumption that the process of oogenesis in animals follows a uniform pattern, with two main variants. One variant is that the oogenesis continues either uninterruptedly or cyclically throughout reproductive life, e.g., most teleosts, all amphibians, most reptiles, and possibly a few mammals. The other variant is that the oogenesis occurs only in fetal gonads, and oogonia neither persist nor divide mitotically during sexual maturity - for instance in cyclostomes, elasmobranchs, a few teleosts, perhaps some reptiles, all birds, monotremes, and with a few exceptions, all eutherian mammals [80-82].

The advantage of the "storage" theory is that it is easy to understand: The extragonadal primordial germ cells migrate into developing ovaries, achieve sex-specific properties, multiply, and complete meiotic prophase of oocytes, which form primordial follicles serving a reproductive function up to menopause in humans.

The essential disadvantage is the requirement for storage of female gametes for up to several decades prior to utilization. Under such conditions, there is a higher probability of genetic alterations in stored oocytes due to the long influence of environmental and other hazards. On the other hand, the "storage" doctrine supports evidence in invertebrates and lower vertebrates that oogenesis continues throughout reproductive life. What is the advantage of oocyte storage in higher vertebrates from the Darwinian evolutionary theory point of view on the development of animal species toward higher and more adaptive forms of life and reproduction, e.g., frogs vs. mammals?

The "continued formation" theory proposes that primordial germ cells degenerate and new oocytes form during adulthood cyclically from the OSC [78,83], and that new oocytes are formed throughout life, and in phase with the reproductive cycle, from germinal epithelium (i.e. OSC) of the adult mammal, at the same time as vast numbers of already-formed oocytes are eliminated through atresia [3].

The advantage of the "continued formation" theory is that it defines a uniform capability of oocyte and follicular renewal in all adult females throughout all species, making this doctrine acceptable from the evolutionary insight. The disadvantage is that it is not easy to define a distinct pattern of this process between the species, for instance the apparent formation of new oogonia in adult prosimian primates [84-86], vs. a more cryptic process in adult humans [11,87].

#### 3.1.1. The prime reproductive period theory

**Figure 1:**
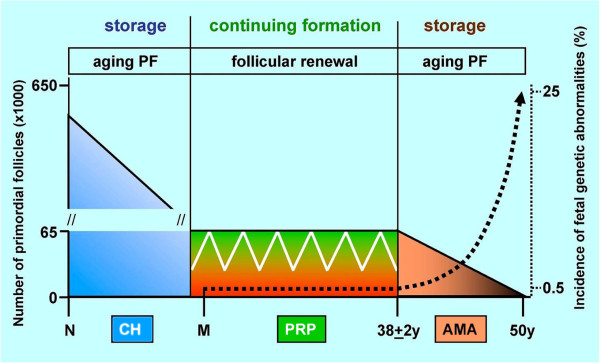
**The prime reproductive period doctrine. **The incidence of trisomic fetuses (dotted line) exponentially increases after 38+2 years of age, i.e., after the termination of follicular renewal during the prime reproductive period (PRP). White line indicates fluctuation of primordial follicle numbers due to their cyclic atresia and renewal during the prime reproductive period. PF, primordial follicles; N, neonate; CH, childhood; M, menarche; AMA, advanced maternal age. Adapted from [94] with permission, © Informa Healthcare, London, UK.

We attempted to establish harmony between the storage (lack of follicular renewal) and continued formation (presence of follicular renewal) theories by proposing the "Prime reproductive period" theory [88]. According to the "Prime reproductive period" theory, the "storage" doctrine fits two periods of the life in humans, that between the termination of fetal formation of new primordial follicles and the advanced puberty or premenarcheal period (about 10–12 years, when the primordial follicles are not renewed), and that following the end of the prime reproductive period at 38+/-2 years of age (termination of follicular renewal), until the menopause. On the other hand, the "continued formation" doctrine accounts for the follicular renewal during the prime reproductive period, which ensures the availability of fresh oocytes for healthy progeny. During the prime reproductive period, the number of primordial follicles does not show a significant decline in human [89] and mouse females [90] due to the replacement of aging primordial follicles by follicular renewal [11,87,91]. In adult ovaries, 50-70% of primordial follicles exhibit degenerative changes [92]. Atresia of primordial follicles declines during the premenopausal period [93], allowing a significantly reduced number of persisting primordial follicles to remain functional in humans for another 10–12 years, after the termination of the follicular renewal. While there are no consequences of oocyte aging for the progeny during childhood, the oocytes persisting after termination of the prime reproductive period accumulate genetic alterations resulting in an exponentially increasing number of fetal trisomies and other genetic abnormalities with advanced maternal age (Figure 1; reviewed in [11]).

### 3.2. A reversal of the oocyte storage to the continued oocyte formation theory and new perspectives in the treatment of POF and ovarian infertility caused by a lack of ovarian follicles with functional oocytes

The oocyte storage theory, developed in the middle of the last century, remained unaffected until 1990'. However, in 1995, it was shown that, similarly to adult mice [78] and prosimians [95], ovarian follicular renewal exists in adult human females [87]. In functional adult human ovaries certain segments of OSC descend into the ovarian stroma and fragment into individual small granulosa cell nests. In other OSC segments germ-like cells were detected, and they migrated into the ovarian stroma, associated with the microvasculature, and eventually aggregated with granulosa cell nests. Hence OSC may be involved in the formation of new primordial follicles in adult human ovaries [87].

The human ovarian study was expanded in 2004 [11], by showing that OSC in adult human ovaries originate from ovarian TA, which is formed perinatally by a mesenchymal transformation of the fetal OSC. In contrast to males, adult human female gonads do not preserve fetal germ line stem cells. Differentiation of OSC converted into primitive granulosa and germ cells from the bipotent mesenchymal cell precursors of TA in adult human ovaries represents a sophisticated adaptive mechanism created during the evolution of female reproduction. It has also been shown that the number of new adult primordial follicles is not determined by the number of new germ cells emerging cyclically during the periovulatory periods of the prime reproductive period, but by the number of available granulosa cell nests. The superfluous intravascular germ cells increase to the oocyte size and die in the medullary vessels. This suggests that transplantation of germ cells into human ovaries lacking granulosa cell nests will cause their death and not formation of new adult primordial follicles. Collectively, the data indicated that the pool of primordial follicles in adult human ovaries does not represent a static but a dynamic population of differentiating and regressing structures. An essential mission of such follicular turnover may be elimination of spontaneous or environmentally induced genetic alterations of oocytes in resting primordial follicles due to the termination of the follicular renewal [11].

In addition, the study in 2005 [7] for the first time demonstrated that cultured OSC exhibit neo-oogenesis *in vitro*. The neo-oogenesis was detected regardless of the advanced patient's age or existing premature ovarian failure (POF), except for the ovaries not exhibiting *in vivo* a presence of the OSC [94]. The fertilization of *in vitro* developed mature oocytes requires intracytoplasmic sperm injection, since the *in vitro* developed eggs do not express surface zona pellucida proteins - (see Chapter 10.4.1. below), required for sperm affinity [96]. It has been shown that intracytoplasmic sperm injection into zona-free human oocytes results in normal fertilization and blastocyst development [97] and oocyte morphology does not affect fertilization rate, embryo quality and implantation rate after intracytoplasmic sperm injection [98]. Moreover, *in vitro* developing oocytes may utilize a phylogenetically ancient mechanism, known from Drosophila ovaries [99]. They divide in order to produce several satellite (nurse) cells, which are exploited to provide additional organelles (see Chapter 10.4.1. below) required for the oocyte growth, that are *in vivo* provided by granulosa cells injecting the ooplasm and forming a Balbiani body [11].

In another 2005 article [100] it was shown that in midpregnancy human fetal ovaries, the granulosa and secondary germ cells also develop from the OSC and contribute to the formation of fetal primordial follicles.

Neo-oogenesis in adult mice, described in 1920' and 1930' [78,101], was in 2004 confirmed by another group of investigators using several lines of evidence [91]. The authors argued that the fetal germline stem cells do not disappear at birth but persist in adult mouse ovaries [91].

In 2009, it was shown that isolated mouse germline stem cells transplanted into ovaries of infertile mice developed into mature oocytes in antral follicles and produced offspring [102] (see Chapters 11 and Chapter 13. for more details).

Finally, in 2012, germline stem cells purified from functional adult human ovaries were shown to produce new primordial follicles when injected into functional human ovarian tissue and transplanted into immunodeficient mice [103] (see also Chapter 11.). The study is important, since it is the first direct confirmation of our report from 1995 that functional adult human ovaries contain germ cells and exhibit follicular renewal [87].

Collectively, the above reports published from 1995 until now, clearly demonstrate that neo-oogenesis and follicular renewal exist in adult mammalian females with functional ovaries, including humans [104]. It remains to be determined whether ovaries of reproductive-aged women with terminated follicular renewal after the prime reproductive period and ovaries of women with POF also carry germline stem cells, and if so, whether such cells can produce new primordial follicles in their ovaries lacking granulosa cells.

In our opinion, adult mammalian ovaries do not preserve fetal germline stem cells but are capable of producing germ cells by the asymmetric division of OSC (see Chapter 4.2.5.). The only conceivable way to enable women with ovarian infertility to have their own offspring is to collect ovarian tissue and establish a culture of OSC with ovarian stromal cells substituting for granulosa cells. The *in vitro* developed mature eggs (see Chapter 10) can be fertilized [94], with subsequent ART management of the collected embryos. This approach can not be used in women lacking *in vivo* OSC or TA precursors in their ovaries (see Chapter 10.7.).

### 3.3. Primordial germ cells

It is now well known that mammalian primordial germ cells originate from uncommitted (totipotent) somatic stem cells, known as embryonic stem cells (ESC) in the inner cell mass of the blastocyst expressing *STELLAR* and deleted azoospermia-like (*DAZL*) proteins of human germ cells, oocytes, and ESC [105,106]. The sex commitment of germ cells is determined by local gonadal environment - signals produced by neighboring somatic cells [107]. Studies of mouse embryos, in which genetically marked cells were introduced at the 4- and 8-cell stage blastomere, have shown that such cells can either become germ or somatic cells [108]. This suggests that no specific germ cell commitment exists prior to implantation. During the postimplantation period, mouse germ cells are not identifiable before seven days after fertilization [109], suggesting that germ cells differentiate from somatic lineage [110]. It has also been shown that cellular differentiation of grafted embryonic cells does not depend on where the grafts were taken, but rather where they are placed [111].

After primordial germ cells enter the developing embryonic gonad, they commit to a developmental pathway that will lead them to become either eggs or sperm, depending not on their own sex chromosome constitution but on whether the gonad has begun to develop into an ovary or a testis. The sex chromosomes in the gonadal somatic cells determine which type of the gonad will develop, as a single *Sry* gene on the Y chromosome can redirect a female embryo to become a male (reviewed in [107]).

## 4. Human embryonic and fetal ovaries - mechanisms of oocyte formation

### 4.1. Human embryonic ovaries

Ovarian differentiation begins before follicles form. It is characterized by the evolution of the OSC from coelomic (peritoneal) mesothelium in the region of the gonadal ridge, organization of the rete ovarii developing from mesonephric ducts, and development of oocytes from germ cells. In human embryos, primordial germ cells arise outside the urogenital ridge, in the dorsal endoderm of the yolk sac at 24 days of developmental age. They migrate by ameboid movements into indifferent gonadal primordial tissue at 28–35 days [112]. After reaching the urogenital ridge, the primordial germ cells expressing *VASA*, a protein which is required for germ cell maintenance and function, and initially accumulate among OSC of the developing gonads [113]. Differentiation of an indifferent gonad into an ovary or a testis takes place during the second month [114]. At the age of nine weeks, female gonads show a marked development of rete cords with lumen formation. The rete reaches the center of the ovary at 12 weeks, when meiosis of oocytes begins. The nuclei of the germ cells lie close together in clusters without clearly defined cell membranes. These syncytia are surrounded by slender stromal (mesenchymal) cells [112].

### 4.2. Human fetal ovaries

Developing fetal ovaries are filled with numerous germ cells and maturing oocytes expressing *VASA* at 15 weeks of age, but at the same age the developing testes contain only scattered *VASA* positive germ cells in seminiferous tubules [113]. Numerous germ cells 10 micrometers in diameter are present in human fetal OSC, and they often exhibit a tadpole-like shape, suggesting their ability to migrate [115,116].

The first primordial follicles are formed in the human fetus after month four. This is substantially latter than the early embryonic occurrence of primordial germ cells. The delayed appearance of primordial follicles may be caused by a requirement for activated MDC with formation of primitive granulosa cells from bipotent OSC and also association of activated MDC with a formation of primordial follicles (see below). Activated MDC are detected in the second trimester human fetuses [117].

Follicle formation always begins in the innermost part of the cortex, close to the rete ovarii, which is essential for follicular development. Follicles will not form if it is removed before formation of primordial follicles has started [118]. Formation of the follicle requires attachment of granulosa cells to the oocyte surface and closure of the basement membrane around this unit. At five months of fetal age, the ovary contains its peak population of oocytes. In human fetal ovaries at the developmental age 5.5 months, newly differentiated oogonia are found to lie within and just bellow the OSC (plate 17C in Ref. [119]). At seven months of intrauterine life the last oogonia enter meiosis (reviewed in [112]).

#### 4.2.1. Origin of secondary germ cells and granulosa cells from fetal ovarian stem cells

The OSC have been implicated in the formation of oocytes in mice and humans [2,3,11,78,87,100,114,120], and it also has been suggested that the OSC are a source of granulosa cells in fetal and adult mammalian ovaries [11,87,115,121-123]. The formation of germ cells from OSC is, however, a selective process. In a given time, only some OSC are transformed into germ cells [87].

**Figure 2:**
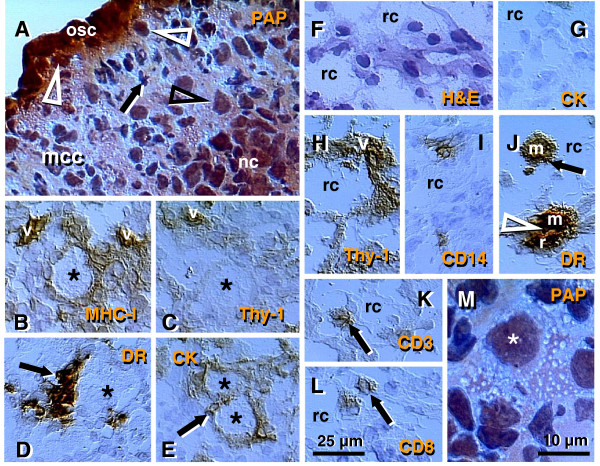
**The human fetal ovary (24 weeks). **Papanicolaou (PAP) staining and immunohistochemistry as indicated in panels. **A**) Secondary germ cells descend (white arrowheads) from the OSC between mesenchymal cell cords (mcc), enlarge within the cortex (black arrowhead) above the nuclear cluster (nc) or syncytium of germ cells. The arrow indicates a mesenchymal type cell. Expression of MHC class I (**B**), Thy-1 (**C**), DR of activated MDC (**D**), and cytokeratin (CK) accompany primordial and primary follicles (asterisks) and vessels (v). **F**) H&E staining of rete ovarii showing rete channels (rc). The rete shows no CK expression (**G**) but show high Thy-1 staining (**H**). The presence of CD14+ primitive MDC (**I**), DR+ MDC (**J**) and CD3 (**K**) and CD8 T cells (**L**). **M**) Numerous oocytes exhibit degenerative changes (vacuolization). A scale bar in (**L**) for panels A-L. See text for details. Adapted in part from [100] with permission, © Humana Press.

**Figure 3:**
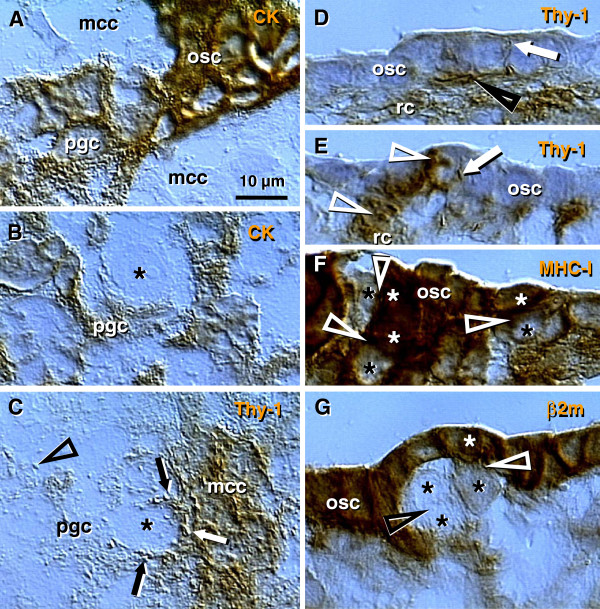
**The human fetal ovary (24 weeks). A**) Sprouts of primitive granulosa cells (pgc) originating from OSC between adjacent mesenchymal cell cords. **B**) In the cortex the primitive granulosa cells associate with available oocytes (asterisk). **C**) Pericytes (white arrow) in mesenchymal cell cords release large quantities of Thy-1 (black arrows and arrowhead) among adjacent oocytes and primitive granulosa cells. **D** and **E**) Rete cord (rc) extensions underline OSC and secrete Thy-1 (arrowheads) collapsing into spikes (arrows). **F** and **G**) Secondary germ cells (black asterisks) originating by asymmetric division of OSC (white asterisks) show depletion of MHC heavy (**F**) and light chain (**G**). Staining as indicated in panels (see Figure 2 legend). **G**) beta 2 microglobulin (beta 2m) = MHC class I light chain. Asterisks indicate germ cells/oocytes. Abbreviations and arrows/arrowheads are explained in the text. Adapted in part from [100] with permission, © Humana Press.

**Figure 4:**
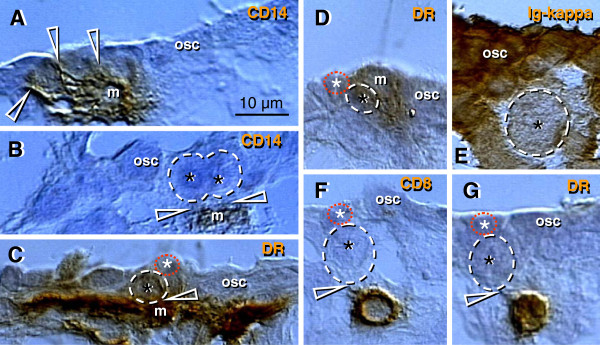
**The human fetal ovary (24 weeks). **CD14+ MDC (m, **A**) exhibit extensions (arrowheads) among some OSC, and accompany (**B**) symmetrically dividing (arrowheads) secondary germ cells. **C** and **D**) Germ cells (black asterisks) originating by asymmetric division from OSC (white asterisks) are accompanied by DR+ MDC (m), and also by CD8+ (**F**) and DR+ (**G**) T cells. **E**) Ig kappa light chain of immunoglobulins (Ig-kappa) is depleted in emerging germ cells. Adapted in part from [100] with permission, © Humana Press.

Figures 2, 3, 4 show morphological and immunohistochemical observations in midpregnancy human fetal ovaries. The germ cells within OSC are smaller (white arrowheads, Figure 2A; PAP staining) when compared to oocytes positioned deeper in the cortex (black arrowhead). Oocytes with well-defined cytoplasm (black arrowhead) lie among smaller cells with round or elongated nuclei (black arrow). Beneath the well-defined germ cells lies a nuclear cluster (nc) or syncytium of germ cells, and the entire area is surrounded by mesenchymal cell cords (mcc), i.e., extension of the rete cords into the cortex.

Figures 2B-E show fetal primordial follicles and associated mesenchymal cells. In the innermost part of the cortex follicles (asterisk, Figure 2B) develop in close vicinity to the microvasculature (v). Note the strong major histocompatibility heavy chain class I antigens (MHC-I) expression by endothelial cells and moderate expression by granulosa cells. Figure 2C shows secretion of Thy-1+ intercellular vesicles (arrow) from vascular pericytes (v) to the fetal primordial follicle (asterisk). An arrow in Figure 2D demonstrates the large activated MDC (HLA-DR^+^) associated with a growing primary follicle (asterisk). Figure 2E shows moderate cytokeratin (CK) expression by granulosa cells (arrow).

#### 4.2.2. Rete ovarii channels contain immune system-related cells

Figures 2F-L show the rete at the center of the fetal ovary. Staining with H&E (Figure 2F) shows the loose character of the rete, containing spacious lumina or rete channels (rc). Cells forming the rete ovarii do not express CK (Figure 2G), but do express Thy-1 differentiation protein, and the strongest Thy-1 expression is characteristic of pericytes accompanying rete vessels (v, Figure 2H). The rete ovarii also contains CD14^+^ (primitive) small MDC (Figure 2I) differentiating into large activated [class II major histocompatibility antigens ( HLA-DR) +] MDC (m, Figure 2J), which migrate through the channels (arrow) and interact (arrowhead) with resident MDC (r). In addition, T cells expressing CD3 (Figure 2K) or CD8 (Figure 2L) of cytotoxic/suppressor T cells are also present in the rete channels.

#### 4.2.3. Degeneration of fetal oocytes

Many germ cells and oocytes within the developing ovary degenerate [112]. The maximum of oocytes in human fetal ovaries (5–7 millions) is present in the sixth fetal month, and they are reduced to one million birth birth. Figure 2M shows a degenerating oocyte (asterisk) accompanied by an irregular layer of granulosa cells. Note extensive oocyte cytoplasmic vacuolization.

#### 4.2.4. Origin of primitive granulosa cells

Sprouts of primitive granulosa cells (pgc, Figure 3A) originate from the OSC which migrate into the ovary. Individual sprouts of granulosa cells are surrounded by mesenchymal cell cords (mcc), and primitive granulosa cells show a decrease in CK expression compared to OSC (osc, Figure 3A). Primitive granulosa cells associate with available oocytes (asterisk, Figure 3B) to form fetal primordial follicles. Pericytes accompanying microvasculature in mesenchymal cell cords release large quantities of Thy-1^+^ intercellular vesicles (arrows, Figure 3C), which then migrate between adjacent oocytes and primitive granulosa cells. These intercellular vesicles collapse into the characteristic empty "spike-like" structures (arrowhead, Figure 3C) after reaching their targets, indicating release of their vesicular content.

Rete cords (rc, Figure 3D and E; rete extensions) consisting of mesenchymal cells, underlie segments of the OSC. Pericytes adjacent to the OSC secrete Thy-1^+^ material among OSC. This material consists of intercellular vesicles (arrowheads, Figure 3D and E) converted into empty "spikes" (arrows).

#### 4.2.5. Secondary germ cells originate by asymmetric division of ovarian stem cells

Some cells within the OSC show asymmetric division (white arrowheads, Figure 3F and G) accompanied by a diminution of MHC-I and light chain (beta2m) expression in one of the daughter cells (black vs. white asterisks, Figure 3F and G). The size of these cells substantially increases compared to typical OSC. Such cells resemble intraepithelial germ cells [116], and subsequently divide symmetrically (black arrowhead and black asterisks, Figure 3G).

#### 4.2.6. Monocyte-derived cells and T cells accompany origin of secondary germ cells

Why are only some OSC transformed into germ cells? It has been suggested that to become a germ cell, the OSC receive an impulse from ovary-committed bone marrow cells, such as monocytes and T cells, in a milieu of favorable systemic (hormonal) conditions ( [100] and Table 1). During ovarian development the immune system-related cells migrate through the rete ovarii and interact with resident MDC (Figure 2I-L), and this may result in their ovarian commitment.

**Table 1:** Working model of age-associated changes of ovary-committed bone marrow cells (OC-BMC) and hormonal signals (LH/hCG & E2) required for the initiation & resumption of oogenesis in human ovaries

**Period of life**	**OC-BMC**^**3**^	**LH/hCG**^**4**^	**E2**^**5**^	**Oogenesis**
*First trimester - midpregnancy*	yes	yes	yes	yes^6^
*Last trimester - newborn*	yes	**no**	yes	**no**^6^
*Postnatal - menarche*	yes	**no**	**no**	**no**^7^
*The prime reproductive period*^*1*^	yes	yes	yes	yes^6^
*Premenopause*^*2*^	**no**	yes	yes	**no**^**7**^
*Postmenopause*	**no**	yes	**no**	**no**^6^

^1 ^From menarche till 38 +/-2 years of age.

^2 ^From 38+/-2 years till menopause.

^3 ^MDC & T cells with commitment for stimulation of OSC to germ cell transformation.

^4 ^Levels corresponding to the mid cycle LH peak, or more (hCG levels should be 10x more, since it has only a 10% affinity to the LH receptor compared to that of LH - see Ref. [124]).

^5 ^Levels corresponding to the preovulatory E_2_ peak, or more.

^6 ^Confirmed.

^7 ^Predicted.

Adapted from [100] with permission, © Humana Press Inc.

In the upper cortex adjacent to the OSC, primitive CD14+ MDC exhibit extensions among some OSC (arrowheads, Figure 4A), and accompany (arrowheads, Figure 4B) the symmetric division of germ cells (asterisks). Activated MDC exhibit the morphology of mature dendritic cells (DC) (Figure 4C). Focal HLA-DR staining is seen among OSC (Figure 4D), suggesting that DC may undergo apoptosis similar to that observed in the normal stratified epithelia of adults [125,126] and/or some OSC may express HLA-DR when activated. Strong binding of Ig-kappa (most probably fetal IgM) is apparent in OSC but not the germ cells (asterisk, Figure 4E). T cells migrating through the rete channels and cords associate (arrowhead, Figure 4F) with the emerging germ cells (black vs. a white asterisk) and exhibit an activation (HLA-DR expression) pattern (Figure 4G). The association of MDC and T lymphocytes with the OSC suggest that they are ovary-committed, possibly occurring during their migration through the rete ovarii channels (Figure 2I-L).

Together, the origin of the germ cells from the OSC is a process driven by ovary-committed bone marrow cells. The number of ovary-committed bone marrow cells interacting with the OSC may determine the number of germ cells actually originating in the ovaries. High binding of immunoglobulins to OSC may prevent them from spontaneous (not driven by ovary-committed bone marrow cells) transformation into germ cells. Heat-inactivated serum in media of OSC cultures lacks immunoglobulins, and, therefore, the OSC proliferate and differentiate into oocytes without the need of the presence of MDC and T cells (see Chapter 10).

In contrast to the association of T cells with the origin of OSC-derived secondary germ cells *in vivo*, T cells were not found accompanying (i.e., to be required for) follicular growth. Both processes were accompanied by activation of Thy-1+ pericytes and association of MDC, however.

#### 4.2.7. Conclusions on the origin of secondary germ cells

These observations indicate that secondary germ cells originate by asymmetric division of the OSC. This is quite complex, since it requires a sequential involvement of the immune system and TCS related cells and molecules. This includes the involvement of primitive MDC, activation of Thy-1+ vascular pericytes, the interaction of activated (HLA-DR+) MDC, and involvement of activated (HLA-DR+) T cells. Once formed, the germ cells undergo symmetric division required for crossing over of chromosomes. Next, they attain the tadpole like shape, enabling them to leave the OSC and enter the ovarian cortex, where they differentiate into oocytes. The oocytes then associate with OSC-derived granulosa cells to form fetal primordial follicles. Therefore, the OSC have the dual potential to differentiate either into somatic granulosa cells or female secondary germ cells, depending on the local cellular signaling and hormonal conditions [100].

An interesting question is whether the OSC are committed toward production of secondary germ cells per se, once differentiation from peritoneal mesothelium in developing embryonic gonads begins. Alternatively, such commitment may require the arrival of extragonadal primordial germ cells. Although extragonadal primordial germ cells may degenerate after entering the gonad, they nevertheless play an important role in gonadal development. In the chick, the germ cells are first recognizable in the crescentic area of the germ-wall endoderm as early as twenty-four hours of incubation [127]. Reagan [128] cut out this crescentic area, in which the primordial germ cells were supposedly located. The operated chicks were then further incubated and killed for examination after varying lengths of time. In no instance, where the removal of this sex cell area was complete, did germ cells arise from somatic cells of the gonads, even after establishment of the OSC. In the normal chick, the OSC are well formed on the fourth day of incubation, and the primitive ova are clearly recognizable among them [129]. But in Reagan's operated chicks, even after five days of incubation, no germ cells were recognized [128]. These observations indicate that the production of secondary germ cells from the OSC require OSC commitment induced by primordial germ cells.

**Figure 5:**
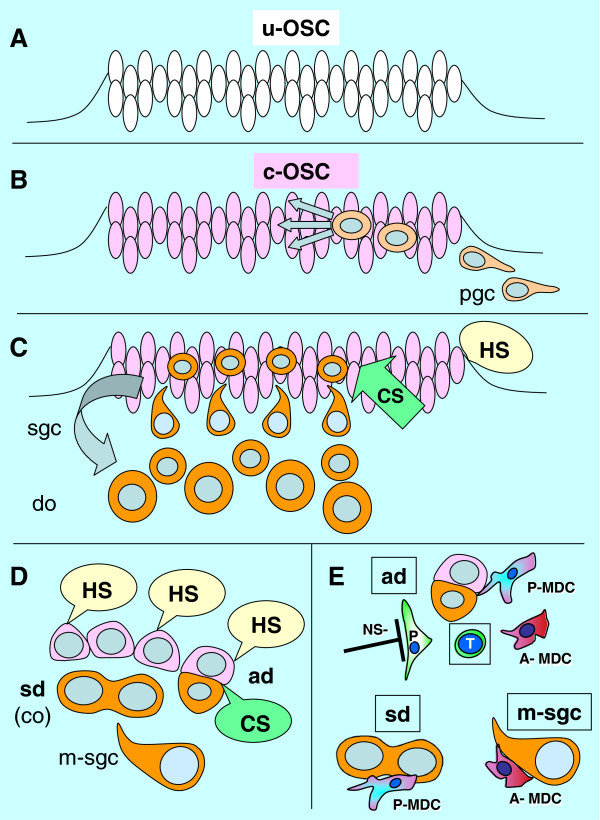
**Model of OSC commitment for production of secondary germ cells. A**) The uncommitted OSC (u-OSC) is present during sixth week of gestational age, prior to the arrival of primordial germ cells (pgc). **B**) Primordial germ cells invade OSC during seventh week and cause commitment of OSC (c-OSC) for production of secondary germ cells (sgc). **C**) The primordial germ cells degenerate and secondary germ cells are produced from OSC influenced by hormonal signaling and cellular signaling (MDC, Thy-1 pericytes, and T cells). The secondary germ cells enter ovarian cortex and differentiate into definitive oocytes (do). **D**) All OSC are influenced by systemic hormonal signals (HS), but only those influenced by CS undergo asymmetric division (ad) followed by symmetric division (sd) required for crossing over (co). Tadpole-like migrating secondary germ cells (m-sgc) leave OSC and enter the ovarian cortex. **E**) Origination of secondary germ cells from OSC by asymmetric division appears to require primitive (CD14+) MDC (P-MDC), activated pericytes (P) with a lack of suppressive neural signaling (NS-), activated (DR+) MDC (A-MDC) and activated (DR+) T cells (T). Adapted from [69] with permission, © Transworld Research Network.

A proposed model of OSC commitment for the production of secondary germ cells is shown in Figure 5. Uncommitted ovarian surface (coelomic) epithelium cells (u-OSC, Figure 5A) are present in human embryos during the fourth week of development, prior to arrival of primordial germ cells. The primordial germ cells (pgc, Figure 5B) invade the OSC during the fifth week and commit the OSC (c-OSC) for production of secondary germ cells. The primordial germ cells degenerate and secondary germ cells (sgc, Figure 5C) are produced from OSC influenced by hormonal and cellular signaling. Hormonal signaling includes estradiol (E2) and hCG, cellular signaling includes MDC, Thy-1 pericytes, and T cells. The secondary germ cells enter the ovarian cortex and differentiate into definitive oocytes (do). All OSC are influenced by hormonal signaling, but only those influenced by cellular signals undergo asymmetric division (ad, Figure 5D) followed by the symmetric division (sd) required for crossing over (co). Tadpole-like migrating secondary germ cells (m-sgc) enter the ovarian cortex.

The involvement of cellular signaling in the origin of secondary germ cells by asymmetric division of OSC (ad, Figure 5E) is complicated. It requires primitive and activated MDC, activated pericytes [(P), with permissive autonomic neural signaling (NS-), i.e., a lack of neural inhibition], and activated T cells (T). The thymus-derived ovary-committed T cells may eventually diminish with the age-induced thymic regression. This may be why the development of new germ cells ceases by the end of the third decade of life (Figure 1 and Table 1). The symmetric division of germ cells follows (sd, Figure 5E) and appears to require primitive MDC (see Figure 4B and below). Migration of secondary germ cells (m-sgc, Figure 5E) is accompanied by activated MDC, which contributes HLA-DR to germ cells (see Chapter 6.2).

Another interesting question is why the developing ovary exhibits so high number of germ cells developing into oocytes, and why the oocytes enter meiotic prophase at the time when the rete ovarii developing from mesonephric ducts reaches the center of the ovary. This contrasts with scarce male gametes and a lack of meiotic prophase in developing male gonads [130]. An important aspect for unique meiotic activity of female germ cells and oocytes appears to be the prevention of mesonephric cell migration and testis cord formation in developing ovaries [131]. Hence, arrest of some oocytes of developing ovaries in meiotic prophase appears to help determine ovarian structure preventing the tendency to develop testicular morphology.

Most fetal oocytes are not preserved till adulthood, but undergo the process of perinatal demise. Nevertheless, it is possible that fetal differentiation of oocytes and primordial follicles, not functionally required until at least puberty and during sexual maturity, may play an important role in programming the time span for the existence of periodical follicular renewal during the prime reproductive period in adulthood [132] (see Chapter 7.).

## 5. Cessation of oogenesis in prenatal human ovaries

The origin of new human oocytes and fetal primordial follicles ceases after the second trimester of fetal intrauterine life, possibly due to the diminution of circulating human chorionic gonadotropin in the fetal blood [100]. Thereafter (perinatally), the layer of loose mesenchymal cells forming ovarian TA develops by mesenchymal conversion of OSC, exhibiting some features of the OSC (CK expression), possibly originating from epithelial-mesenchymal conversion of the OSC [11,12], as described in OSC cultures [133]. These TA mesenchymal cells could be converted back into the OSC by mesenchymal-epithelial conversion, i.e., into bipotent stem cells capable of differentiating into granulosa and secondary germ cells in adult human ovaries [11,87,134]. This, however, may not happen prior to puberty or menarche, due to a lack of hormonal signaling (Table 1, [100]).

## 6. Oocyte and follicular renewal in humans during the prime reproductive period

During adulthood, the CK+ fibroblast-like mesenchymal cells in TA are progenitors of bipotent OSC in humans. One advantage of mesenchymal cell progenitors is that they are much more resistant to epigenetic and environmental hazards, compared to highly sensitive oocytes, as evident from exponentially growing fetal abnormalities after termination of oocyte and follicular renewal from OSC (Figure 1).

### 6.1. Origin of new granulosa and germ cells from bipotent ovarian stem cells

Follicular renewal in adult human ovaries from bipotent OSC, i.e. precursors of germ and granulosa cells, is a two-step process, consisting of the new formation of epithelial cell nests (primitive granulosa cells) and the formation of new germ cells [11].

#### 6.1.1. Origin of new granulosa cells

Similar to fetal ovaries, new granulosa cells develop in adult human ovaries from the bipotent OSC, but the process and results are different. In human fetal ovaries, primitive granulosa cells develop between mesenchymal cell cords and form in the adjacent cortex solid epithelial cords, similar to those described in adult rodent ovaries [3]. These primitive granulosa cells are then available for an unlimited number of germ cells entering epithelial cords to form primordial follicles in fetal human ovaries (see Figure 3A-C) and adult rodent ovaries [135]. The formation of new primordial follicles in adult human ovaries is, however, different, as they originate from the association of a single germ cell with a single epithelial nest of primitive granulosa cells. Since the number of epithelial granulosa nests formed during the follicular phase of the each menstrual cycle is lower than the number of new germ cells formed during the periovulatory periods, excessive germ cells degenerate (see Chapter 11.3.).

**Figure 6:**
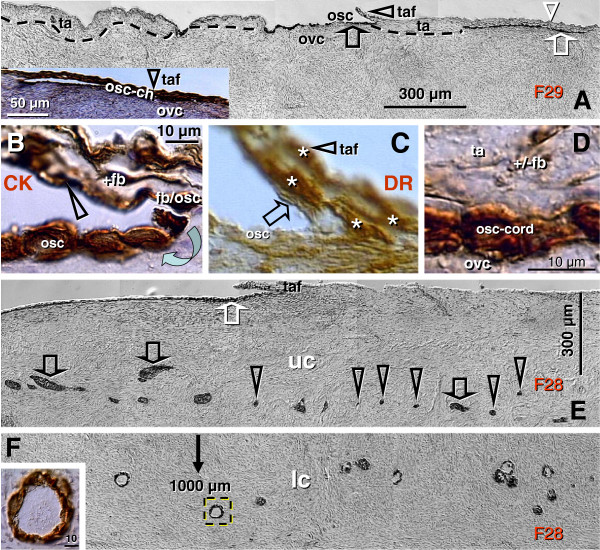
**Origin of new granulosa cells from OSC during the prime reproductive period in adult human ovaries. A**) Panoramic view of ovarian surface and adjacent cortex. Dashed line indicates interface between TA and stroma of the ovarian cortex. osc and black arrow - ovarian stem cells; taf and black arrowhead - TA flap; white arrowhead - a lack of OSC above the TA; white arrow -bilaminar epithelial cord. **B**) Detail from (A) shows association of CK+ (brown color) fibroblasts (+fb,) with the TA flap surface (arrowhead), transition from mesenchymal to epithelial morphology (fb/se), and ovarian stem cells (osc, arched arrow). **C**) A parallel section to (B) showing numerous DR+ MDC (asterisks) in the TA flap. Note DR expression also in early OSC (arrow). **D**) Detail from (A) shows CK+ epithelial cord consisting of two layers of epithelial cells and lying between the ovarian cortex (ovc) and TA (ta). Note diminution of CK immunoexpression in TA fibroblasts (+/-fb). E) Epithelial cords (black arrows) fragmenting into granulosa cell nests (arrowheads in the upper ovarian cortex (uc). White arrow CK+ OSC associated with the TA with flap. **F**) Lower ovarian cortex (lc) with primordial follicles. Arrow indicates distance from the ovarian surface, dashed box indicates follicle shown in the inset. F29 indicates female age in years. Bar in (D), for (B-D). Panels A, B, D-F adapted from [11], © Antonin Bukovsky; panel C from [134], © Wiley-Liss, Inc. with permission.

The initiation of granulosa cell nest formation consists of an extension of TA (ta, Figure 6A) over the ovarian surface, the so called TA flap (taf arrowhead). F29 indicates the age of the female ovaries which were studied. This TA flap contains CK+ fibroblast-like cells (+fb, Figure 6B, detail of TA flap from panel A), which are converted into OSC precursors (fb/osc) and OSC (rounded arrow and osc). For the emergence of granulosa cells, this process is associated with numerous DR+ MDC (asterisks, Figure 6C) and early OSC show weak DR expression (arrow). Inset in Figure 6A shows formation of the bilaminar OSC channel (osc-ch). The channel collapses into a bilaminar OSC cord (white arrow, Figure 6A) and is overgrown by CK unstained TA (white arrowhead). Detail of CK+ OSC cord adjacent to the ovarian cortex (ovc) is shown in Figure 6D. Dashed line in Figure 6A indicates TA and ovarian cortex interface - note that the formation of the OSC cord begins on the ovarian cortex lacking the TA.

An ovary from another female in the prime reproductive period (F28) shows in Figure 6E an upper ovarian cortex (uc) with a fragmentation of OSC cords (black arrows) into primitive granulosa cell nests (arrowheads). Figure 6F shows lower ovarian cortex (lc, close to the ovarian medulla - 1000 micrometers from the ovarian surface) containing primordial follicles. Dashed box indicates primordial follicle shown in the inset. New primitive granulosa cell nests move to this level during each periovulatory phase in the prime reproductive period and associate with intravascular or migrating germ cells to form new primordial follicles (see below).

#### 6.1.2. Origin of new germ cells

**Figure 7:**
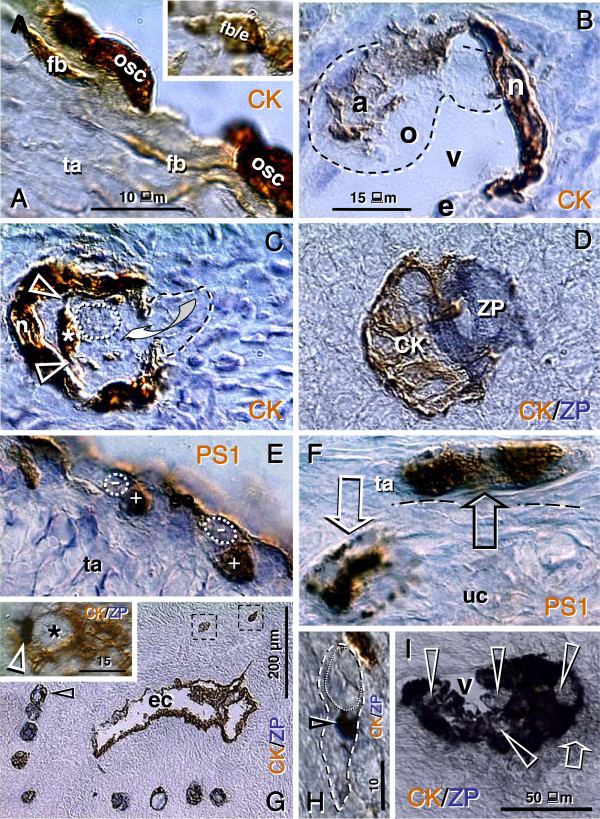
**Follicular renewal in adult human ovaries. A**) Cytokeratin (CK) positive (brown color) cells of fibroblast type (fb) in tunica albuginea (ta) exhibit mesenchymal-epithelial transition into OSC (osc). Inset shows a transitory stage (fb/e). **B**) The CK+ epithelial nest (n) inside of the venule (v) in deep ovarian cortex, which extends an arm (a) to catch the oocyte (o, dashed line) from the blood circulation. e = endothelial cell. **C**) The nest body (n) and closing "gate". A portion of the oocyte (dashed line) still lies outside of the complex, and is expected to move inside (arched arrow). The oocyte contains intraooplasmic CK+ extensions from the nest wall (arrowheads), which contribute to the formation of CK+ paranuclear (Balbiani) body (asterisk). The oocyte nucleus is indicated by a dotted line. **D**) The occupied "bird's" nest type indicates a half way oocyte-nest assembly. CK indicates cytokeratin staining of primitive granulosa cells and ZP indicates zona pellucida expression in the assembling oocyte. **E**) Segments of OSC show cytoplasmic PS1 (meiotically expressed carbohydrate) expression. Asymmetric division of OSC gives rise to cells exhibiting nuclear PS1 (+ nuclei vs. - cell daughters) and descending from the OSC into tunica albuginea (ta). **F**) In tunica albuginea, the putative germ cells increase in size, show a symmetric division (black arrow) and exhibit development of cytoplasmic PS1 immunoexpression when entering (white arrow) the upper ovarian cortex (uc). **G**) Association of primary follicles (arrowhead) with the cortical epithelial crypt (ec). Dashed boxes indicate unassembled epithelial nests. Inset shows origination of germ-like cells among CK+ cells (CK) in epithelial crypt. Note ZP+ segment (white arrowhead) associated with unstained round cell nucleus (asterisk). **H**) Migrating germ cells with tadpole shape (dashed line), unstained nucleus (dotted line) and ZP+ staining of the intermediate segment (arrowhead). **I**) Some medullary vessels (v) show accumulation of ZP+ (dark color) degenerating oocytes with unstained nuclei (arrowheads). Arrow indicates ZP release. Adapted from [11], © Antonin Bukovsky.

For the formation of secondary germ cells in adult human ovaries during the prime reproductive period, the single layer of OSC is formed at the ovarian surface from CK+ TA fibroblasts (Figure 7A) during the periovulatory period. The granulosa cell nests (n, Figure 7B) associate with ovarian vessels (v) in the lower ovarian cortex to catch the circulating oocytes (o) from the blood. During follicle formation, extensions of granulosa cells penetrate the ooplasm (arrowheads, Figure 7C) like a sword in the shield to form a single paranuclear Balbiani body (asterisk). It contains additional organelles which the oocyte needs to develop later into the mature egg [136]. Formation of adult primordial follicles is documented by double color staining for CK of the nest cells (CK, Figure 7D) and zona pellucida (ZP) glycoprotein of assembling oocytes [11].

Germ cells originate by asymmetric division of OSC differentiating above the TA (Figure 7E). The germ cells showing PS1 meiotically expressed oocyte carbohydrate antigen [(+) vs. (-) sign] enter the TA, where they undergo a symmetric division (black arrow, Figure 7F) required for the crossing over, and then enter (white arrow) the adjacent upper cortex (uc) and cortical vessels. During vascular transport the germ cells increase in size, and are picked up by epithelial nests (see above panel B).

An alternative origin of germ cells in human ovaries is by OSC crypts originating from OSC invaginations into the deep cortex. Such epithelial crypts (ec, Figure 7G) show transformation of OSC into ZP+ (arrowhead in inset, Figure 7G) germ cells (asterisk), which are capable of saturating neighboring nests of primitive granulosa cells (dashed boxes, Figure 7G) to form new primordial follicles (arrowhead). Hence, even if the OSC is not available (or is destroyed) the cortical epithelial crypts are an alternative source of germ cells. For formation of adult primordial follicles, however, the nests of primitive granulosa cells should be available. When the epithelial nests are not available locally, the ZP+ tadpole-like germ cells resembling sperm (Figure 7H) migrate to attain cortical vessels and utilize vascular transport to reach distant targets (see Figure 7B). Finally, the intravascular oocytes, which were not utilized in the formation of adult primordial follicles, degenerate (arrowheads, Figure 7I) in the medullary vessels (v).

### 6.2. Involvement of the immune system-related cells

**Figure 8:**
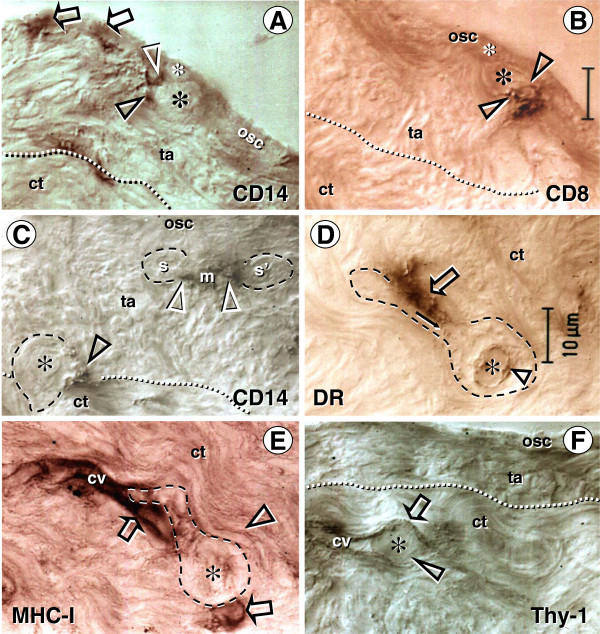
**Immune type cells influence commitment of OSC. **Staining of the adult human OSC (osc), tunica albuginea (ta), and an adjacent cortex (ct) for CD14 of primitive MDC and HLA-DR of activated MDC, CD8 of cytotoxic/suppressor T cells, MHC class I heavy chain, and Thy-1 glycoprotein of pericytes, as indicated in panels. Large asterisks and dashed lines indicate putative germ cells. **A**) Primitive MDC associate with OSC (arrows) and accompany (arrowheads) origination of germ cells by asymmetric division of OSC (asterisks). **B**) Asymmetric division is also accompanied by extensions from T cell (arrowheads) into a putative germ cell daughter. **C**) Primitive MDC accompany (white arrowheads) symmetric division (s-s') of germ cells in tunica albuginea and their migration into the adjacent cortex (ct). **D**) Migrating tadpole-like germ cells are accompanied by activated MDC (open arrow), and HLA-DR material is apparent in the cytoplasm (solid arrow) and in the nuclear envelope (arrowhead). **E**) The germ cells associate with cortical vasculature (cv) strongly expressing MHC-I (arrows vs. arrowhead), enter and are transported by the bloodstream (**F**). Adapted from [87], with permission, © Blackwell Publishing, Oxford, UK.

Like in fetal ovaries, the ovary-committed bone marrow cells are associated with oogenesis in adult human ovaries [87]. CD14 primitive MDC interact with OSC (arrows, Figure 8A) and asymmetrically dividing OSC (white and black arrowheads) - note small cell size of the OSC stem cell daughter (yellow asterisk) compared to the secondary germ cell daughter (black asterisk). CD8+ T cells accompany germ cells through asymmetric division of OSC (arrowheads, Figure 8B). Primitive MDC (m, Figure 8C) are also associated with symmetric division of germ cells (white arrowheads, Figure 8C) in TA and with germ cells entering (black arrowhead) the upper cortex (ct). Activated MDC are associated (arrow, Figure 8D) with migrating germ cells, and these cells associate (arrow, Figure 8E) with the cortical vasculature (cv) and utilize (arrowhead and asterisk, Figure 8F) vascular transportation (arrow) to reach distant destinations.

### 6.3. Localization of SCP3 in adult human and monkey ovaries

In a study by Liu et al. [137] the authors compared the expression of meiotic entry synaptonemal complex protein-3 (SCP3) in fetal and in functionally undefined adult human ovaries. The authors argued that SCP3 protein was not detectable in the TA, OSC, or in oocytes of adult primordial follicles in adult human ovaries, and concluded that no meiotic oocytes are present in ovaries during adulthood. In a subsequent commentary, Tilly and Johnson [138] stated that the lack of evidence in this study of neo-oogenesis in adult human females is not convincing, and that some data of Liu et al. [137] actually support the existence of neo-oogenesis in adult women.

We studied SCP3 expression using the same SCP3 antibody, and found the immunoreactivity with segments of TA, OSC, and in oocytes of one third of adult primordial follicles in functional midcycle adult human and monkey ovaries [139]. Functional ovaries without evidence of ongoing follicular renewal (midfollicular, mid- and late luteal phases) indeed lacked staining for SCP3 in TA, OSC, and in adult primordial follicles.

Functional ovaries with ongoing follicular renewal, i.e., with unoccupied nests of primitive granulosa cells (often occupying a portion of the vascular lumen), oocyte/nest assemblies, and degenerating superfluous oocytes in ovarian medullary vessels (see Figure 7I), were observed during the postovulatory (early luteal) phase of the menstrual cycle. In these ovaries a strong SCP3 expression was shown in some segments of TA. Such TA cells exhibited a mesenchymal pattern, characteristic of the OSC precursors. The differentiated OSC layer also showed SCP3 immunostaining. SCP3 immunoexpression was also evident in differentiated OSC of postovulatory monkey ovaries.

Moreover, the SCP3 immunostaining was observed in the nucleoli of oocytes in some adult primordial follicles in human and monkey ovaries. Laura L. Tres has reported that male germ cells exhibit nucleolar SCP3 expression during early stages of meiotic prophase [140]. In addition, an SCP3+ synapsis of two chromosomes was detected in human primordial follicle oocyte, possibly representing XX chromosomal synapsis, since sex chromosomes start synapsis during early zygotene, before autosomes synapse [140]. Rare SCP3+ oocytes (less then 10%) were detected in midfollicular phase ovaries. The most frequent expression (10 to 30% of primordial follicle oocytes) was found in postovulatory ovaries during the early luteal phase in younger (till 38 years of age) women. However, at the age of 42 years, postovulatory ovaries showed no SCP3 expression.

No staining of oocytes was observed in three younger women studied during the mid- and late luteal phases and in PCO ovaries [139]. SCP3 expression was also reported in juvenile and adult mouse ovaries [102].

These observations indicate that SCP3 is expressed in adult human, monkey, and mouse ovaries. SCP3 expression detected in TA stem cells indicates that preparation for meiotic activity may have already occurred at the level of TA OSC progenitors, and meiotic prophase activity may continue and terminate in the oocytes of newly formed adult primordial follicles. SCP3 expression in about 30% of primordial human follicles during follicular renewal indicates that during each menstrual cycle about 30% of adult primordial follicles are replaced. These data also confirm that follicular renewal occurs during the prime reproductive period, but not thereafter, and is not present in PCO ovaries.

### 6.4. Summary on follicular renewal in adult human ovaries

**Figure 9:**
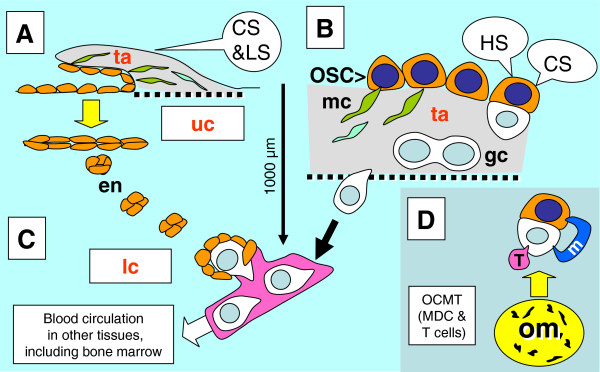
**Survey of follicular renewal in adult human ovaries. **Follicular renewal in adult human ovaries is a two-step process based on mesenchymal-epithelial transition of tunica albuginea (ta) bipotent progenitor cells into OSC. **A**) Epithelial nests: Segments of the OSC directly associated with the upper ovarian cortex (uc) are overgrown with tunica albuginea, which forms a solid epithelial cord that fragments into small epithelial nests (en) descending into the lower ovarian cortex (lc) and associating with the blood vasculature. Initiation of this process may require cellular and other local signaling (CS & LS), possibly neural [87]. **B**) Germ cells: Under the influence of cellular signaling (CS) of ovary-committed MDC & T cells (OC-BMC) and hormonal signaling (HS), some OSC covering the tunica albuginea undergo asymmetric division and give rise to new germ cells (gc). The germ cells subsequently divide symmetrically and enter adjacent cortical blood vessels. During vascular transport, they are picked up by epithelial nests associated with vessels. **D**) The ovary-committed bone marrow cells originate from bone marrow (MDC) and from lymphoid tissues (T cells) carrying "ovarian" memory (om), which diminishes with utilization; when spent, the follicular renewal ceases, in spite of persisting hormonal signaling (Table 1).

The origin of new epithelial nests and germ cells and their assembly in adult human ovaries is schematically depicted in Figure 9. Under the influence of cellular and other local signaling (CS & LS, Figure 9A), including immune system-related activated MDC [134] and neural signals and molecules [87], the TA overgrows the upper ovarian cortex (uc, Figure 9A) and its mesenchymal cells attain CK expression and transform into OSC. In this way, the bilaminar OSC layer is formed, which descends into the cortex (arrow), and fragments into epithelial nests (en) of primitive granulosa cells [87]. The epithelial nests move through stromal rearrangements into the lower cortex (lc) [11], where they associate with cortical vessels (venules) to pick up circulating oocytes.

Under the influence of hormonal and cellular signaling (HS, CS, Figure 9B) of ovary-committed bone marrow cells (arrow, Figure 9D) derived from immune system-related structures supposed to carry an "ovarian memory" (om, [12]), the mesenchymal OSC precursors (mc, Figure 9B) differentiate into the OSC covering not the cortex, as above, but the TA layer (ta, Figure 9B), and produce germ cells (gc) by asymmetric division [11]. This is followed by a single symmetric division of germ cells required for crossing over. Subsequently, the germ cells enter the upper cortex, associate with blood vessels, and enter the circulation (arrow, Figure 9B). They assemble with epithelial nests of primitive granulosa cells in the lower cortex (lc, Figure 9C). The circulating germ cells may contaminate other tissues, including bone marrow, and eventually degenerate in the ovarian medullary vasculature (Figure 7I), to which they appear to have an affinity for homing. For a schematic description of alternative origin of germ cells from epithelial crypts in adult ovaries (see Figure 7G and [11]).

The rete ovarii is absent in adult ovaries. Ovary-committed bone marrow cells during adulthood may originate from bone marrow and lymphoid tissues. It has been suggested that during fetal developmental immune adaptation an "ovarian" memory is build within the developing immune system for support of follicular renewal by ovary-committed bone marrow cells during adulthood [12]. It appears that the termination of follicular renewal occurs at about 38 years of age, resulting in a significant decline of oocyte numbers in human ovaries [89] and an abrupt increase in the rate of primordial follicle loss [141]. The termination of follicular renewal is not necessarily due to the lack of germ cells, which are detected in aging mouse ovaries [142], but rather due to the lack of formation of new granulosa cell nests (see below). This may be programmed by a significantly later emergence of granulosa cells in developing ovaries (4^th^ month of intrauterine life) compared to the very early emergence of germ cells (1^st^ month of intrauterine life) - see Chapter 4 and below.

## 7. Developmental immune adaptation and determination of the aging of the ovary and other tissues

In normal individuals, the first organ affected by aging is the thymus [143] and next the ovary in human females [144,145]. There is a strong correlation between the period at which an organ is present during early ontogeny and that organ's functional longevity. For instance the heart, which differentiates very early, can function more than one hundred years. However, the ovary, in which the granulosa cells and primordial follicles differentiate later, does not function for more than half of that time [1]. We have proposed that the later the differentiation of certain type of tissue occurs during early ontogeny, the earlier its function expires during adulthood [66].

During developmental immune adaptation, through the end of the second trimester of intrauterine life in humans [28], the differentiating tissues are recognized by the developing lymphoid (immune) system as self. However, depending on the time at which a certain tissue arises during developmental immune adaptation, a memory can be built for how long such tissue would be supported by tissue-specific mesenchymal cells. Immune system-related cells (MDC and T cells) are present in peripheral tissues, and influence the differentiation of tissue cells [126], including the formation of germ and granulosa cells and differentiation of primordial follicles [87,100].

Monocyte-derived cells play an important role in regulation of the immune system. These cells control the function of tissue lymphocytes associated with tissue-specific cellular differentiation [126]. Lymphoid tissues not only produce cells promoting differentiation of tissue-specific epithelial and parenchymal cells, but also receive information from peripheral tissues via afferent lymph. This information is transmitted by “veiled” MDC, a subpopulation of tissue MDC. They are HLA-DR positive, highly immunogenic and present antigens to T cells in the draining lymph nodes [146-149].

In the fetal ovary presumptive memory cells reside in the rete ovarii, and immature MDC and T cells migrate through rete channels toward the ovarian surface. There they participate in the development of germ cells from OSC [100]. Similar interactions of immune cells with OSC were described in the ovaries of adult women [87]. During adulthood, however, no rete is present in ovaries, so memory cells may reside in the bone marrow and lymphoid tissues. The immune system shows a significant functional decrease between 35 and 40 years of age [150] and concomitantly ovarian follicular renewal ceases [11].

### 7.1. Thymus and reproduction

It has been suggested that thymic peptides play a role in determining the reproductive lifespan of females [151,152]. A relationship between age-associated thymic involution and the diminution of ovarian function is supported by the alteration of ovarian function seen in neonatally thymectomized mice [13]. Additionally, in congenitally athymic (nude) mice, follicular loss is first evident at two months of age. This is specifically due to a reduction in the number of primordial follicles. The initial ovulation is delayed until two and a half months of age, compared to the first ovulation at one and a half month old normal mouse females. By four months, an overall reduction in all fractions of the follicle population occurs in nude mice, and ovulation ceases [153]. Interestingly, the absence of the thymus might also be responsible for the lack of hair in nude mice, due to the lack of thymus-derived T cells, which may be required for hair development.

### 7.2. The working hypothesis

**Figure 10:**
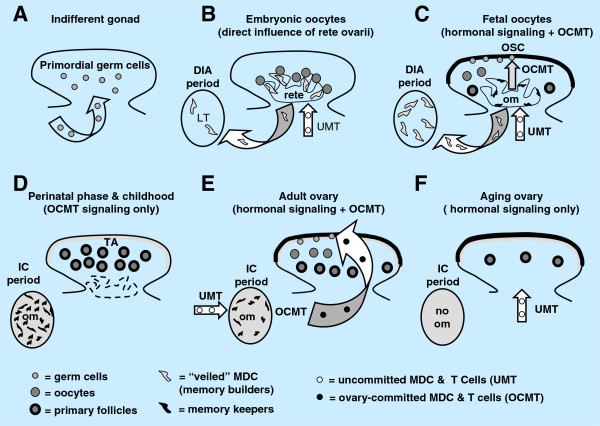
**Evolution of ovaries during developmental immune adaptation and their behavior during immune competence. A**) Primordial germ cells imprint the OSC for production of secondary germ cells (see Figure 5B vs. 5A). **B**) Development of rete ovarii and lymphoid tissue (LT). Uncommitted MDC and T cells (UMT) saturate rete ovarii to be converted into ovary-committed MDC and T cells (OCMT). **C**) Secondary germ cells originate by asymmetric division of OSC under the influence of rete-derived OCMT and hormonal signaling. The ovary commitment is also transferred into draining lymphoid tissues (arched arrows). During the perinatal period immune competence (ic) is initiated, the ovarian memory (om) is built and the rete ovarii regresses. During childhood the OCMT is available but hormonal signaling is absent until menarche. **E**) During the prime reproductive period (from menarche to 38+/-2 years of age) OCMT and cyclic hormonal signaling cause cyclic formation of germ cell and renewal of primordial follicles. **F**) After the prime reproductive period the hormonal signalling persists but follicular renewal ceases due to the lack of OCMT. Reprinted from [132], with permission, © Bentham Science Publishers, Ltd.

Our working hypothesis on the role of gonadal environment in the regulation of human oogenesis [132] is presented in Figure 10. After the indifferent gonad is populated with primordial germ cells (Figure 10A), the rete ovarii stimulates differentiation of oocytes from secondary germ cells (Figure 10B). During developmental immune adaptation, the rete is populated by uncommitted MDC and T cells (UMT), from which the MDC differentiate into the veiled cells. The veiled cells transmit information on oocytes from the rete into the developing lymphoid tissues (curved arrowhead, Figure 10B). The MDC in the rete ovarii then become ovarian memory cells able to convert UMT passing through the rete channels into ovary-committed bone marrow cells. These ovary-committed bone marrow cells, with appropriate hormonal stimulation, induce the development of germ cells from the OSC (Figure 10C). The number of veiled cells populating lymphoid tissues increases further.

When the developmental immune adaptation is terminated, the rete ovarii degenerates and oogenesis ceases, due to diminution of hormonal signaling (fetal hCG barrier [100]). The ovarian TA develops from OSC (epithelial-mesenchymal transformation), and the number of ovarian memory cells (om; the transformed veiled cells) in lymphoid tissues is set (Figure 10D).

Around menarche and during the prime reproductive period, the hormonal signaling and ovary-committed bone marrow cells resume cyclic oogenesis to replace aging primordial follicles undergoing atresia (Figure 10E). The cyclic follicular renewal during adulthood requires a cyclic supply of ovary-committed bone marrow cells, and their generation in lymphoid tissues (ovary-committed T cells in particular) causes depletion of the pool of memory cells. Hence, the pool of ovarian memory cells in lymphoid tissues, but not the pool of primordial fetal follicles, is what is set during mammalian fetal development.

Once the available pool of ovarian memory cells is consumed, oogenesis and follicular renewal cease, in spite of the presence of hormonal signaling (Figure 10F). Remaining adult primordial follicles persist and are utilized until gone. However, the aging oocytes accumulate genetic alterations and may become unsuitable for ovulation and fertilization. Postmenopausal ovaries are reported to carry occasional follicles with degenerated oocytes (reviewed in [144]).

The initial pool of ovarian memory cells in lymphoid tissues may be reduced due to the retardation of normal ovarian development during embryonal and fetal immune system adaptation [1]. If the period for which the primordial follicles differentiate during developmental immune adaptation is shorter (delayed or terminated earlier), the period of follicular renewal after menarche ceases earlier compared to normal ovaries, and POF with secondary amenorrhea results. The restriction of primordial follicle development during adaptation may result in a lack of follicular renewal after menarche and primary amenorrhea (early POF). Primordial follicles and ovarian memory in the lymphoid system can also be depleted by cytotoxic chemotherapy. Interestingly, the incidence of POF after such chemotherapy increases with age. In women <20 years old the incidence is 13%, in 20-30 years is 50%, and >30 years is 100% [154]. This suggests a diminution of ovarian memory in the lymphoid system with age and increasing sensitivity to chemotherapy.

Together, we speculate that a lack of follicular renewal may be caused by age associated exhaustion of memory cells in the lymphoid system, which are required to generate effector cells that migrate to ovaries and stimulate the transformation of OSC into primitive granulosa and germ cells. Premature ovarian failure may be caused by delayed ovarian development during developmental immune adaptation [1], by earlier termination of developmental immune adaptation, or by cytostatic chemotherapy affecting both the existing pool of primordial follicles and ovary-committed mesenchymal cells required for follicular renewal. Patients with POF have been found to have abnormalities in the function of circulating monocytes, activated lymphocytes, NK cells, and exhibited other immune abnormalities [155-157], including anti-ovarian autoantibodies [158], suggesting a relationship of immune system alteration to the pathogenesis of POF.

### 7.3. Premature failure of ovaries with primordial follicles and animal models

Premature ovarian failure is the result of a lack of adult primordial follicles within the ovaries of women less than 40 years of age [159]. However, POF is also often clinically associated with follicular resistance to gonadotropins, called "hypergonadotropic amenorrhea.” Here ovaries contain normal primordial or even antral follicles not responding to gonadotropins by the production of estrogens. No woman with primary amenorrhea has been reported to ovulate or conceive with her own oocytes, but more than one third of the women with secondary amenorrhea were pregnant at least once before developing hypergonadotropic POF. A quarter of them had evidence of ovulation after the diagnosis was established, and 8% of those with secondary amenorrhea later conceived [157].

Animal models (rats and mice) indicate that there are two types of POF with primordial and antral follicles within the ovaries. The first, persistent ovarian immaturity, can be induced by inhibition of ovarian development (temporary inhibition of androgen receptor expression) with estrogens during developmental immune adaptation, while the second, premature ovarian aging, can be induced by acceleration of ovarian development (premature expression of an androgen receptor) with androgens [37,67].

### 7.4. The tissue control system theory and a "stop-effect" of monocyte-derived cells

The TCS theory [38,63,65,67,126,160] addresses the role of vascular pericytes, MDC, and T and B cells in the regulation of tissue function. It proposes that MDC stimulate early differentiation of tissue-specific (epithelial, parenchymal, neural, and muscle) cells. Monocyte-derived cells also regulate expression of epitopes of tissue specific cells, and in this way control their recognition by circulating tissue-committed T cells and antibodies. Such T cells and antibodies promote the advanced differentiation of tissue cells, which ultimately results in the aging and apoptosis of these cells [126].

By the end of the developmental immune adaptation in early ontogeny, the MDC encounter the most differentiated tissues in a tissue-specific manner, and prevent them from differentiating beyond the encoded state by the so-called "stop effect." The nature of the "stop effect" may reside in the inability of monocyte-derived cells to stimulate differentiation of tissue cells beyond the encoded stage. Retardation or acceleration of certain tissue differentiation during developmental immune adaptation causes a rigid and persisting alteration of this tissue function. The ability of monocytes to preserve tissue cells in a functional state declines with age. This is accompanied by functional decline of various tissues within the body, including the ovary, resulting in menopause and an increased incidence of degenerative diseases in humans.

In large mammals including primates developmental immune adaptation is terminated during intrauterine life, while in small laboratory rodents it continues for several postnatal days, ending about one week after birth [28]. Estrogens given to neonatal rats and mice inhibit ovarian development. During adulthood such rat females exhibit persisting ovarian immaturity, characterized by retardation of follicular development [65] despite normal levels of gonadotropins [161,162]. This shows that suppression of early ovarian development results in persisting ovarian immaturity, resembling POF and gonadotropin resistence of ovarian follicles. Injection of estrogens in neonatal mice (days 0-3) caused permanent anovulation, but mice injected later (days 3-6; closer to the end of developmental immune adaptation) show a resumption of ovulatory cycles after initial anovulation [163]. Hence, persisting ovarian immaturity can result in delay of normal ovarian function. Since the incidence of degenerative diseases increases with age, one would expect a tendency of the "stop-effect" to shift up with age [1]. This could explain how persistent ovarian immaturity may change to a functioning ovary.

On the other hand, injection of androgens causes premature ovarian aging which persists throughout adulthood. Androgen-induced anovulation can be prevented by neonatal injection of a thymic cell suspension from immunocompetent prepubertal normal female donors, but not if given from animals prior to completion of developmental immune adaptation [17]. This suggests that certain thymic cells (thymocytes, or thymic MDC) of normal immunocompetent females carry information on the differentiation of ovarian structures, and this information can be transferred to immunologically immature neonatal rats.

However, when a low dose of androgens is injected during developmental immune adaptation, the rats exhibit a delayed anovulatory syndrome. Ovaries exhibit the onset of normal function after puberty (~40 days of age), but premature aging of the ovary occurs between 60-100 days [164]. This delayed manifestation of ovarian dysfunction resembles human POF with secondary amenorrhea, as well as some human degenerative diseases with autoimmune character, which also occur after a shorter (juvenile diabetes mellitus) or longer (Alzheimer's disease) period of normal tissue function.

An application of the TCS theory on the regulation of tissue function via the "stop effect" is described in Ref. [1]. In normal tissues, the functional cells are present during developmental immune adaptation and the tissue-specific cells are "parked" in the functional state during adulthood. Retardation of cell differentiation during adaptation results in persisting immaturity (POF with primary amenorrhea) and an acceleration of premature aging (POF with secondary amenorrhea, degenerative diseases). If the tissue was absent during adaptation, like CL, it is handled as a "graft" [160].

Note that the functional stage of cell differentiation differs between distinct tissue types, being very low in the vagina (in the absence of hormonal stimulation the basal/parabasal cells are only present), low in the brain, skeletal muscle, and pancreatic beta cells (lack of intraepithelial lymphocytes), moderate in the gut (presence of intraepithelial lymphocytes), and high in the skin (apoptosis of surface keratinocytes) [165].

### 7.5. The immune system memory and aging of the body

The "ovarian memory" built within the lymphoid system can be viewed as a charged battery, which is drained by periodical follicular renewal. The higher the charge during the developmental immune adaptation, the longer it will last, and *vice versa*. The possible involvement of developmental immune adaptation in the programming of ovarian function and various types of POF can be extrapolated to other tissues in the body as well. From this point of view, the degenerative changes of the immune system with advancing age could be responsible for the aging of other tissues and the body in general.

## 8. Former and current views on ovarian oogenesis and follicular renewal

### 8.1. Milestones of the oocyte storage theory

The current belief that all oocytes in adult mammalian gonads should be from the fetal period was originally based on the “continuity of germ plasm” theory. It declared that an essential condition for the development of another embryo is the retention of a part of the progeny of the primary impregnated germ cell, and that germ cells have a different character as compared to the somatic cells, since the somatic cells serve as nurse cells for the germ cells and cannot be considered as their progenitors [82,166]. This theory assumes that the primary impregnated cell determines subsequent individuals. This, however, contrasts with the tenants of evolutionary theory indicating that surviving species are those who reproduce themselves, mutate, and are capable of transmitting these mutations into the next generations.

### 8.2. Oogenesis in adult prosimians

The original report by Gerard on the unique oogenic activity from germinal cords in the cortex of the ovary of adult *Galago*[84] was followed by studies of ovaries of adult South Indian prosimians, where the origin of multiple oocytes from deep invaginations of OSC was reported [85]. The occurrence of oogonia in adult ovaries of other prosimian species was confirmed subsequently by many investigators (reviewed in [86]). Although the fate of such oogonia is a matter of dispute [167], these observations indicate that new oocytes are formed in adult prosimians, i.e., primate species appear evolutionary much more developed than mice in this regard.

### 8.3. Rodent ovaries

Edgar Allen was the first to suggest the possibility of follicular renewal in mammals during sexual maturity [78]. Evans and Swezy, in studies of oogenesis in adult guinea pigs [3], showed that cells move from the OSC into the ovarian stroma, usually as a solid cord, and one or more cells become enlarged into oocytes; the remaining cells form the granulosa cells. Another possibility is that oocytes are formed along the cord and other cells group themselves around these, forming primordial follicles [3]. We observed both mechanisms in normal adult rat ovaries and showed that a group of cells within the descending OSC cord express ZP proteins. Some of these ZP cells are transformed into tadpole-like cells capable migrating [100]. This enables the development of adult primordial follicles either directly in adjacent areas, or via the blood stream in more distant sites. Follicular renewal was also detected in ovaries of neonatally estrogenized sexually mature rats (ages 45–60 days) [135].

#### 8.3.1. Functional repair of anovulatory mouse ovaries with cultured germline stem cells

Former observations indicating that neo-oogenesis occurs in adult mouse and human ovaries [11,78,87,90,91,139] were in 2009 confirmed by evidence that cultured germline stem cells transplanted into the ovaries of sterilized mice could produce new oocytes and offsprings [102]. Ji Wu and colleagues studied germline stem cell cultures derived from 5 day old and adult mouse ovaries, and their ability to produce functional oocytes when transplanted into the ovaries of infertile mice [102]. The cultured cells were infected with the GFP virus and, when transplanted, they were transformed into oocytes and produced offspring expressing GFP transgene [102]. These observations suggest the possibility of restoring fertility in women with POF [168,169].

### 8.4. Summary on the current views

In conclusion, the prevailing standard for the current doctrine on the storage of mammalian fetal primordial follicles with primordial oocytes from the period of ovarian development is that the process of oogenesis occurs only in fetal gonads, and oogonia neither persist nor divide mitotically during sexual maturity with a few possible exceptions, such as in prosimian primates [81,82].

From our point of view, observations in human ovaries confirm that oogonia do not persist during sexual maturity, since during the prime reproductive period new female gametes and granulosa cells originate from bipotential OSC. Observations from ovaries of adult human and rat females [7,11,87,100,135] and from laboratory mice [78,90,91,170] support a paradigm that the oocyte and follicular renewal in adult females during the prime reproductive period (Figure 1) exists throughout all animal species (see the prime reproductive period theory - Chapter 3.1.1.). We are confident that it is just a matter of time until additional scientists will confirm this.

## 9. Follicular selection

Ovarian follicles are selected at two occasions. Firstly, from the cohort of resting adult primordial follicles some follicles are stimulated to grow. Once selected, the growing follicles either ovulate or degenerate. Secondly, during the midfollicular phase in human females one follicle is usually selected to finish maturation and ovulate and the other large antral follicles degenerate in both ovaries.

### 9.1. Selection of growing (primary) follicles

**Figure 11:**
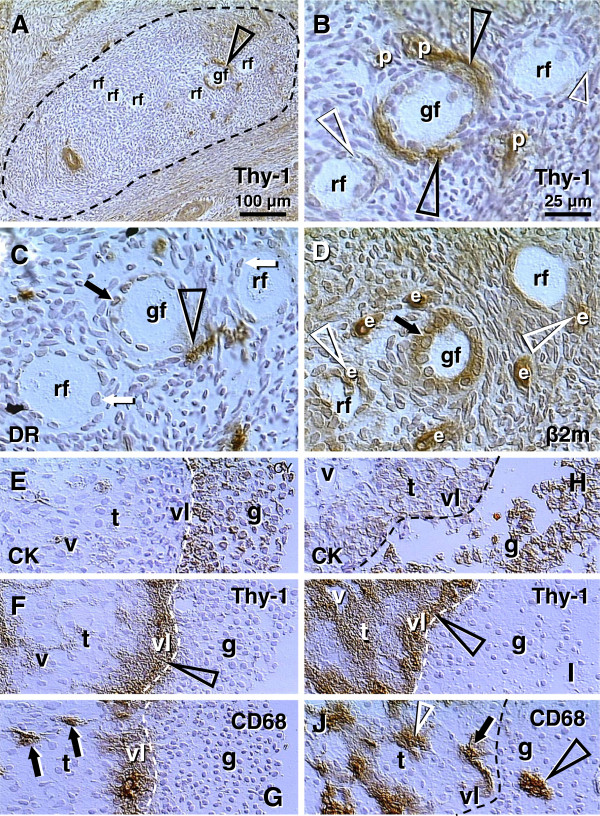
**Follicular selection. **Selection of secondary (**A-D**) and preovulatory (dominant) follicles (**E-G**), and large antral follicle atresia (**H-J**) in the adult human ovary. Staining for Thy-1, HLA-DR (DR), MHC class I light chain (beta 2m) and CD68 of mature MDC, as indicated in panels. Dashed line in **A **indicates an "ovary-in-ovary" area exhibiting diminution of Thy-1 expression by stromal cells. **B**) detail from **A**. **C **and **D** are semi-parallel sections to **B**. Dashed line in **E-J**, follicular basement membrane. rf, resting follicles; gf, growing follicle; p, pericytes; e, endothelial cells; v, microvasculature in theca interna (t); vl, vascular layer adjacent to the follicular basement membrane; g, granulosa layer. Reprinted from [134] with permission, © Wiley-Liss.

Within the human ovary, cohorts of adult primordial follicles occupy distinct areas in the cortex that are characterized by a diminution of Thy-1 protein expression in stromal cells (dashed line, Figure 11A[11,134]). These areas exhibit an "ovary-in-ovary" pattern, and stromal cells show enhanced MHC class I expression (see Figure 11D and [87]). Most of the adult primordial follicles remain in the resting state (rf, Figure 11A), but some show an increase in size and an apparent transformation into growing (primary) follicles (gf). Initiation of follicular growth is triggered by activation (Thy-1 release) of vascular pericytes (p and black arrowheads, Figure 11B) and activated (HLA-DR+) MDC (semi-parallel section, Figure 11C). The MDC secrete HLA-DR^+^ (arrowhead, Figure 11C ), which accumulates in the nuclear envelope of granulosa cells (black vs. white arrows; see also the arrowhead in the germ cell, Figure 8D). Activated MDC also interact with the granulosa cells of preantral follicles, at the site of antrum formation [87]. Figure 11D, semi-parallel section to Figure 11B and C, shows enhanced MHC class I expression in granulosa cells (arrow) of the growing follicle. Enhanced MHC class I expression in the vasculature is apparent by strongly stained endothelial cells (e). Note that vasculature endothelial cells also accompany resting follicles (white arrowheads, Figure 11D), but such vasculature lacks Thy-1+ (activated) pericytes (white arrowheads, Figure 11B).

Why some adult primordial follicles in the given cohort initiate growth and other remain resting is not well understood. Our observations indicate that selective stimulation of some adult primordial follicles to growth and development is initiated by activation (Thy-1 release) of vascular pericytes. Since vessels accompanying follicles are innervated [112], one may speculate that the number of adult primordial follicles entering growth in the given time is controlled by the neural system, which controls the activity of Thy-1+ pericytes and quantitative aspects of tissues (reviewed in [63]). Consequently, the MDC accompanying vasculature are activated too (arrowhead, Figure 11C), in order to stimulate continuation of follicular differentiation.

In other words, the activity of pericytes of resting adult primordial follicles is inhibited by vascular autonomic innervation (AI+). Activity of pericytes in growing (primary and secondary) follicles is devoid of such inhibition (AI-). This assumption is supported by the evidence that tissues lacking autonomic innervation, such as CL and cancers, show activated vascular pericytes [134].

### 9.2. Selection of a dominant follicle

In human ovaries, usually only one dominant follicle is selected for ovulation during the mid follicular phase of each menstrual cycle. This process of follicular selection still remains an unresolved puzzle. Premature stimulation with gonadotropins results in multiple mature antral follicles, suggesting that more than one large antral follicle in the follicular cohort of those developing until the midfollicular phase is capable of maturing. Hence, under normal conditions, there seems to be a competition among growing follicles themselves in an attempt to reach the mature state and suppress the development of others. However, in contrast with this traditional view, our data indicate that the follicles showing more advanced development of theca interna during selection are those destined to degenerate [64]. Hence, a critical role in the process of dominant follicle selection appears to belong to the theca interna compartment.

Figure 11E shows CK staining of a human dominant follicle in the mid-follicular phase with multiple CK+ granulosa cell layers (g) adjacent to the follicular basement membrane (dashed line). Under the follicular membrane is a vascular layer (vl) and theca interna (t) layers with narrow vessels (v). Staining for Thy-1 (Figure 11F) shows high activity of Thy-1 pericytes (arrowhead) in the dominant follicle, which is restricted to the vascular layer. The CD68 MDC (Figure 11G) show no activity within theca interna of the dominant follicle (arrows) but high CD68 release in the vascular layer.

Large antral follicles undergoing atresia in the same ovary show a detachment of granulosa cells from the basement membrane (g, Figures 11H vs. 11E). This is accompanied by marked activation of thecal pericytes (t, Figure 11I vs. 11F) and dilation of thecal vessels (v). In addition, the thecal MDC become highly activated (white arrowhead, Figure 11J vs. arrows, Figure 11G), but those in the vascular layer in regressing follicle show virtually no CD68 release (arrow, Figure 11J). Instead, MDC from the vascular layer invade among detached granulosa cells (black arrowhead).

### 9.3. Novel aspects of follicular selection

Follicles are selected twice during their development (preantral from adult primordial follicles and preovulatory from antral follicles), but the consequences for the remaining follicles are different. First, during basal growth, primary follicles are selected from adult primordial follicles under the control of growth factors of paracrine origin. Unselected adult primordial follicles remain in the resting state. The selection of secondary follicles is associated with activation of pericytes in adjacent microvasculature, possibly due to permissive signals (i.e., lack of inhibition) from autonomic innervation, which is involved in the regulation of quantitative aspects (amounts) of specific cells and structures in tissues from early periods of life [171,172]. Activated pericytes are accompanied by activated perivascular MDC. Hence, during growth initiation, the selected follicles are stimulated for further development.

After attaining the antral stage, follicles become gonadotropin dependent and immature granulosa cells lacking aromatase can be affected by thecal androgens [173]. Hence, premature acceleration of theca interna development may cause follicular atresia via alteration of immature granulosa cells lacking aromatase by thecal androgens. This is associated with conversion of follicular MDC into phagocytes infiltrating the follicular antrum. We show that during selection of a preovulatory follicle, the pericytes in theca interna of nondominant follicles are highly activated and accompanied by activated thecal MDC.

In a dominant follicle, activated MDC are present in the vascular layer adjacent to the follicular basement membrane, and may stimulate maturation of granulosa cells (attainment of aromatase expression). In nondominant follicles, the high activity of pericytes and MDC in the theca interna may cause enhanced production of thecal androgens resulting in atresia accompanied by invasion of MDC among immature granulosa cells.

Hence, it appears that the dominant follicle is selected by a process of temporary retardation of thecal differentiation by a negative influence of autonomic innervation on the activity of thecal pericytes. Indeed, extracts of the superior ovarian nerve have been shown to inhibit androstenedione production by theca interna cells [174,175].

Once the dominant follicle matures into the preovulatory stage, with the ability of mature granulosa cells to convert androgens into estrogens, pericytes and MDC in both thecal and vascular layers show high activity [64]. Taken together, a lack of inhibition of thecal development during the midfollicular phase results in follicular atresia and temporary inhibition of thecal development results in follicular dominance during follicular selection.

### 9.4. Follicular atresia

Follicular atresia and luteal regression are essential mechanisms required for the elimination of unnecessary and aged structures, as well as for normal ovarian function. Elimination of antral follicles undergoing atresia and of degenerating corpora lutea during reproductive years in human females is a fast process, associated with infiltration of activated macrophages [64,160]. There is no reason to expect that the similar process accompanying regression of primordial and secondary follicles [11] will last longer than several days. If at least 60% of all oocytes in adult human ovaries are in various stages of degeneration [92], one may conclude that without follicular renewal the ovarian function will cease in human females within a few months. However, in aging ovaries, the elimination of degenerating ovarian structures appears to be altered [176], possibly due to age-induced alterations of immune system function [176]. Hence, atresia may not affect primordial follicles in aging ovaries [93], and such follicles may persist in spite of an accumulation of genetic alterations of oocytes.

During the prime reproductive period, degeneration may affect groups of primordial and secondary follicles. Immunohistochemically, the follicles undergoing atresia release ZP proteins into the neighboring stroma. This is associated with an altered oocyte morphology and disorganization of the follicular CK+ granulosa layer. In addition, there is a considerable influx of large macrophages into the area from accompanying vessels [11]. Some investigators claim that characteristic morphological features of primordial follicle atresia are often difficult to determine (reviewed in [130]), while others are more confident [93].

In our immunohistochemical study, the assembly of oocytes with epithelial nests was also associated with some release of ZP proteins. Formation of new follicles was characterized by a well-defined oocyte nucleus, intraooplasmic CK+ extensions from the nest cell wall, and formation of the Balbiani body, i.e., structures and processes not apparent during follicular regression. Resting normal primordial follicles and growing secondary/preantral follicles show regular morphology, with no leakage of ZP proteins, and only occasional small tissue macrophages associated with the developing theca [11].

Enhanced follicular atresia was accompanied by the appearance of epithelial nests (fragmented epithelial cords) in adjacent segments of the ovarian cortex. We have shown that these nests are small CK+ spheroidal cell clusters of 20–30 micrometers in diameter. There were also epithelial crypts, likely originating from deep OSC invaginations. These do not communicate with the ovarian surface, as evidenced from serial sections. The movements of epithelial nests and crypts appear to be caused by a rearrangement of stromal bundles, and their migration is probably guided by HLA-DR+ (activated) tissue macrophages. We have also described an alternative germ cell origin from epithelial crypts in the lower ovarian cortex (inset, Figure 7G), accompanied by migration of tadpole like germ cells with ZP+ intermediate segment (arrowhead, Figure 7H), the presence of epithelial cell nests without oocytes (dashed boxes, Figure 7G), and the accumulation of primordial follicles in the neighborhood of epithelial crypts [11] (Figure 7G).

These observations indicate that adult human ovaries exhibiting atresia of primordial and secondary follicles initiate formation of new epithelial nests from epithelial cords with granulosa cell features [122]. This is one of the prerequisites for the formation of new primordial follicles. Cortical crypts, consisting of epithelial cells retaining a relatively embryonic structure of OSC [122,177], appear to be an alternate source of germ cells. Germ cells entering the vasculature may reach epithelial nests at distant destinations, although vascular proximity is not always a requirement for follicular development (see Figure 7G).

## 10. Developmental potential of ovarian stem cells *in vitro*

As indicated above, alteration of ovarian function and the onset of the menopause are associated with the changes in ovarian immunoregulation. Yet, in OSC cultures, such immunoregulation is absent and the neo-oogenesis is renewed, including cultures from POF and postmenopausal ovaries exhibiting a presence of OSC (Table 2). In addition, our observations also indicate that OSC show *in vivo* a strong binding of natural autoantibodies (see Figure 4E), which are absent in OSC cultures. This suggests that the OSC have the ability to differentiate into oocytes if *in vivo* immunoregulation is absent.

**Table 2:** Presence of ovarian OSC and emergence of oogenesis in culture

**Case #**^**1**^	**Age**^**2**^	**POF**	**Diagnosis**	**CK+ OSC/ epithelial crypts**		**Oogenesis in primary and secondary cultures**		**Morulae**
		**pre/post menopause**		**left ov**	**right ov**	**primary**	**secondary**	
1	30	POF	POF	yes	yes	yes	N.A.	no
2	36	pre	PFD^4^	no	yes	yes	yes	no
3	38	POF	POF	**no**	**no**	**no**	N.A.	no
4	39	pre	endometriosis	yes	yes	yes	N.A.	no
5	40	POF	POF	yes	yes	yes	N.A.	no
6	42	pre	fibroids	yes	no	yes	yes	yes
7	43A	pre	menorrhagia	yes	yes	yes	yes	no
8	43B	pre	fibroids	yes	no	yes	yes	no
9	45	pre	endometriosis	yes	yes	yes	yes	yes
10	48A	pre	menorrhagia	no	yes	yes	yes	yes
11	48A	pre	fibroids	no	yes	yes	yes	no
12	**49**^**3**^	pre	PFD	**no**	**no**	**no**	N.A.	no
13	**50**^**3**^	pre	fibroids	**no**	**no**	**no**	N.A.	no
14	**52**^**3**^	post	ovarian cyst	**no**	**no**	**no**	N.A.	no
15	53	post	PFD	no	yes	yes	yes	no
16	55	post	uterine bleeding	yes	no	yes	yes	yes
17	60	post	PFD	no	yes	yes	yes	yes
18	67A	post	PFD	yes	yes	yes	N.A.	no
19	67B	post	PFD	yes	no	yes	N.A.	no

Evaluation of menopausal status, OSC in ovaries, and presence of oogenesis and morulae in primary and secondary ovarian cultures. Data adapted from [178] and [94] with permission, © Informa UK Ltd.

^1^ Age arrangement.

^2^ Letters are added in patients exhibiting identical age.

^3^ Perimenopausal women.

^4^ Pelvic floor disorders (uterine/vaginal prolapse with or without urinary incontinence).

POF, premature ovarian failure; pre, premenopause; post, postmenopause; N.A., not available.

### 10.1. Cell types developing from omnipotent ovarian stem cells

After observations in adult and fetal human ovaries, we asked ourselves if a similar potential for OSC could be demonstrated *in vitro*. Our observations have shown that oocytes, fibroblasts, and epithelial type cells can develop spontaneously, as well as neuronal cells [7].

### 10.2. Culture conditions and techniques

The surface of intact ovaries is gently scraped in an aseptic laminar flow hood with a sterile stainless steel surgery knife blade No. 21 (Becton Dickinson, AcuteCare, Franklin Lakes, NJ). This procedure is selected with the intention to include OSC and some adjacent TA and ovarian stromal cells.

The cells are collected into sterile petri dishes containing tissue culture medium supplemented with heat inactivated 20% fetal bovine serum (FBS; Gibco/BRL, Grand Island, NY) and antibiotics (50 microgram/ml gentamycin, 100 U/ml penicillin, and 100 microgram/ml streptomycin). The tissue culture media utilized is either Dulbecco's Modified Eagle's Medium containing 25 mM HEPES, 4500 mg/L glucose, and phenol red (DMEM-HG; with estrogenic stimuli) or Dulbecco's Modified Eagle Medium/Ham's F12, phenol red free (DMEM/F12; without estrogenic stimuli). No other treatment is imposed during the culture.

The cells are spun down (1000 × g, 5 min, 24 degrees C), diluted in 0.75 – 1.5 ml of supplemented media, seeded in either 6 or 3 wells of a 24-well plate (250-350 microliters per well) (Fisher Scientific, Pittsburgh, PA), and cultured in an humidified atmosphere with 5% CO2 at 37 degrees C. The number of wells is chosen by the size of ovaries. Cells collected from larger ovaries are seeded into six wells, and from small ovaries are seeded into 3 wells. All ovaries involved in the experiment were anovulatory, and no CL detected. The culture medium was changed once after 24 hours. This left only adherent (viable) cells in culture, and eliminated non-adherent cells and the majority of contaminating erythrocytes. The cell cultures were monitored daily by phase contrast microscopy and live cells evaluated by immunohistochemistry after 5–6 days from the initial seeding. Viability of cells is apparent from their active movement, changes in shape, and movement of their nuclei. The number of adherent cells in a single well of 24-well plate ranged between ~100 to 1000 during the late culture period (day 5 or 6).

### 10.3. Estrogens are essential for the neo-oogenesis in vitro

**Figure 12:**
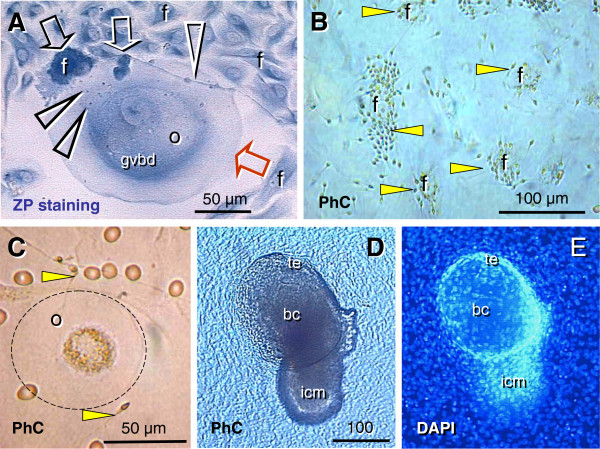
**Oocyte and parthenote development *in vitro. *A**) The oocyte (o) development in OSC culture is accompanied by satellite (black arrow) and neuronal (white arrow) cells. Black arrowheads indicate organelles mowing from the satelite cell into the oocyte and white arrowhead indicates neuronal extension. Note ZP staining of fibro-epithelial cells (f) but no expression of ZP proteins at the oocyte surface (red arrowhead). Sperm associate (arrowheads) with fibroepithelial cells (**B**) but not with the oocytes (**C**). **D **and **E**) The parthenote shows a trophectoderm (te), blastocoele (bc), and inner cell mass (icm). (**A**) shows staining for ZP proteins, (**B**, **C** and **D**) are live cultures in phase contrast (PhC), (**E**) is a DAPI staining of the fixed culture. Adapted from [179], © Cambridge University Press, and unpublished observations.

The OSC cultures maintained in DMEM-HG with estrogenic stimuli (phenol red) showed large cells exhibiting the phenotype of oocytes on day 5. These cells reached 100–180 micrometers in diameter and showed a centrally located germinal vesicle break-down with nucleus and nucleolus (see Figure 12A).

In contrast, utilization of DMEM/F12 without estrogenic stimuli in another culture from the same patients produced no large cells exhibiting an oocyte phenotype on day 5. However, 12 day cultures of ovarian cells showed the presence of oocytes. It is thought that natural estrogenic stimulation is provided by ovarian stromal cells that accompany OSC in culture.

It was found that stromal tissue from human ovaries produces progesterone, androgens, and estrogens *in vitro*[180]. In addition, ovarian stromal cells produce *in vitro* high levels androgens similar to that of the thecal cells [181]. These androgens can be converted into estrogens by fibroblasts [182] present in OSC cultures [7]. The OSC are also capable of secreting hCG [183] and steroid hormones, including estrogens [133]. Hence the hormonal conditions required for transformation of OSC into oocytes *in vivo* (Table 1) also can be achieved *in vitro*. The OSC cultures without estrogenic stimuli showed the presence of granulosa type cells on day 5 [7]. It is useful to mix the 5 day OSC cultures with and without estrogenic stimuli from the same patient in order to accelerate the oocyte maturation. The granulosa cells from culture without estrogenic stimulation may provide the Balbiani body [11] and contribute to the expression of ZP proteins by developing oocytes [184]. Since ZP proteins are sperm ligands [185], their expression by *in vitro* developing oocytes may preclude the need for utilization of intracytoplasmic sperm injection (ICSI) for their fertilization (see below). *In vivo*, new granulosa cells develop in functional ovaries from OSC during the mid follicular phase, when the estrogenic stimuli and LH levels are low, and new oocytes are formed during the periovulatory period, when the estrogenic stimuli and LH levels are high [11,87].

It requires twice as long for cultures initially lacking estrogen to reach a level of estrogens produced by ovarian stromal cells compared to media with sufficient exogenous estrogen (phenol red) level from the beginning of culture.

### 10.4. Development of oocytes and parthenogenetic embryos in vitro

#### 10.4.1. Primary ovarian stem cell cultures

**Figure 13:**
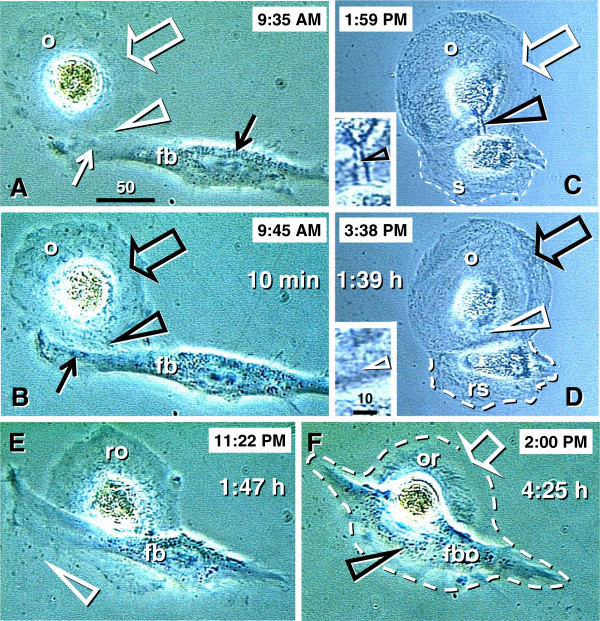
***In vitro *developing oocytes supplied with organelles from fibroblasts resulting in fibro-oocyte hybrids, or by satellite cells produced by the oocytes and exploited for the progression of the oocyte growth. **Images from time lapse photography (time in hours:minutes). **A**) *In vitro* developing oocytes (o) deficient in organelles (white arrow) can be joined (arrowhead) by a fibroblast (fb and white arrowhead). **B**) The optically dense organelles are supplied to the oocyte. **C**) Alternatively, the oocyte is supplied by adjacent satellite cell (s) with an extended tube (black arrowhead; see detail in inset). **D**) When completed, the tube disappears (inset) and the satellite is regressing (rs). **E**) In contrast, the fibroblast moves above the oocyte and releases organelles out (white arrowhead) of the regressing oocyte (ro). **F**) Subsequently, a fibro-oocyte (fbo) hybrid is formed exhibiting oocyte remnants (ro). Bar in **A **for **A-F**. Panels **A-D **adapted with permission from [9], © Wiley-Liss, Inc, and complemented with **E **and **F**.

The advanced development of oocytes (o, Figure 13A) *in vitro* with germinal vesicle breakdown (gvbd) is accompanied by fibro-epithelial (f) satellite cells (black arrow) substituting a granulosa cell-derived Balbiani body to provide additional organelles (black arrowheads) needed by the developing egg. To increase the volume of the oocyte, the human granulosa cell microvilli penetrate *in vivo* deep into the ooplasm of developing oocytes and supply a variety of organelles such as Golgi vesicles, endoplasmic reticulum membranes, and nascent forms of smooth endoplasmic reticulum [136].

*In vitro*, neural type cells are also involved (white arrow, Figure 12A) with an extension (white arrowhead) over the developing oocyte. Note a strong expression of ZP proteins in fibro-epithelial cells on the cell surface but lack of ZP expression at the oocyte surface (red arrow). The presence of sperm causes their association (arrowheads, Figure 12B) with fibro-epitheial cells (f), but not with the oocytes (Figure 12C), due to the lack of surface ZP expression (red arrow, Figure 12A).

Eventually, some oocytes differentiate into parthenotes with a trophectoderm (te, Figure 12D and E), blastocoele (bc) and inner cell mass (icm) [179], similarly to some oocytes in *in vitro* fertilization (IVF) cultures [186]. This indicates that the OSC-derived oocytes developed *in vitro* are functionally similar to the mature follicular oocytes. The lack of ZP expression at the surface of *in vitro* developed oocytes, will, however, mandate the utilization of ICSI for their fertilization.

Images from time lapse photography show that early developing oocytes (o, Figure 13A) are low in optically dense cytoplasmic organelles (white open arrow). They can be joined (arrowhead) by fibroblast-type cells (fb) which provide additional organelles. Such fibroblast-type cells initially show optically dense organelles (black solid arrow) close to the nucleus, but not in the arm extended toward the oocyte (white solid arrow). Within 10 min (Figure 13B), however, optically dense organelles are apparent in the extended arm (solid black arrow) within adjacent oocyte cytoplasm (black arrowhead) and in distant oocyte regions (open black arrow).

Alternatively, developing oocytes (o, Figure 13C) deficient in the cytoplasmic organelles (white arrow) are supplied by satellite cells (s), i.e., nurse cells, which are produced by the oocytes themselves (see below). The satellite cell shows an extended tube (black arrowhead; see also detail in the inset) providing the cytoplasm with organelles for the oocyte. Figure 13D (1 hour, 39 min from Figure 13C) shows that when such a process is completed, the oocyte exhibits an enhanced content of the optically dense organelles (black arrow, Figure 13D) and the tube draining the satellite cell disappears (white arrowhead – see detail in the inset). The satellite cell size is reduced (dashed line) and the content is altered (compare with Figure 13C).

The fate of oocytes supplied by fibroblasts is, however, different. Figure 13E (1 h, 47 min from Figure 13A) shows that the fibroblast moves above the oocyte and releases the organelles out (white arrowhead) of the regressing oocyte (ro). Figure 13F (4h, 25 min) shows fusion of the fibroblast (rich in organelles - black arrowhead) with a structure showing oocyte remnants (or) lacking organelles (white arrow), a so-called fibro-oocyte (fbo) hybrid.

**Figure 14:**
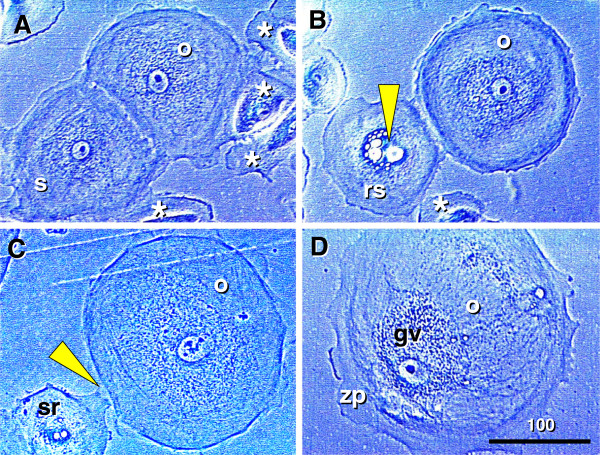
**Oocyte satellites *in vitro. *A**) Early stage after oocyte (o) division producing the satellite (s) cell. Asterisks indicate additional small satellites. **B**) Oocyte growth and regressing satellite (rs) with vacuolization in the perinuclear space (arrowhead). **C**) Progressive oocyte growth and its separation (arrowhead) from the satellite remnants (sr). **D**) Large isolated oocyte exhibits germinal vesicle (gv) and a thick zona pellucida (zp) cytoplasmic membrane. Panels **A-D **adapted with permission from [9], © Wiley-Liss, Inc.

Figure 14A shows an early stage after oocyte (o) division producing the satellite (s) cell. Note a similar content of cytoplasmic organelles; asterisks indicate former (regressing) satellites. Figure 14B indicates that oocyte growth is accompanied by a regressing satellite (rs) exhibiting nuclear alteration and vacuolization in the perinuclear space (arrowhead). Note also depletion of cytoplasmic organelles compared to the satelite in Figure 14A. Figure 14C shows progressive oocyte growth and its separation (arrowhead) from the satellite cell remnants (sr). A large isolated oocyte (Figure 14D) exhibits germinal vesicle (gv) and a thick ZP cytoplasmic membrane (zp).

#### 10.4.2. Secondary ovarian stem cell cultures

**Figure 15:**
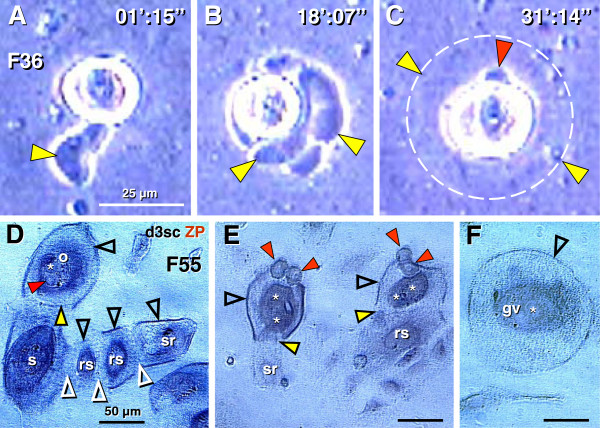
**Differentiation of early oocytes and behavior of oocytes in secondary OSC cultures. A-C**) Time lapse cinematography of oocyte development in secondary OSC culture. **A**) Early developing cell with a cytoplasmic tail (arrowhead). **B**) Multiple cytoplasmic eruptions (arrowheads). **C**) Development of the 50 micrometers oocyte-like cell (yellow arrowheads indicate cell surface, red arrowhead a polar body). Time in minutes':seconds". Movie segments are available in the Additional file 1, supplemental video S1. **D-F**) Day 3 secondary OSC culture stained for ZP (d3sc ZP) - 55 year old postmenopausal women three days afer seeding. **D**) Oocyte (o) with a "chain" of satellite cell (s), regressing satellites (rs), and satelite remnants (sr) interconnected by intercellular bridges (open white arrowheads) and surface ZP expression (open black arrowheads). The "leading" oocyte shows formation of the cell surface in the bridge (solid yellow arrowhead). Asterisk indicates cell nucleus and red arrowhead developing polar body. **E**) Two "leading" oocyte type cells with surface ZP expression, each of which shows expulsion of two polar bodies (red arrowheads) and retention of two pronuclei (asterisks). Yellow arrowheads indicate a line of separation from remnants of the "satellite" cells lacking surface ZP. **F**) Large oocyte (140 micrometers) with surface ZP expression (arrowhead), nucleus (asterisk), and germinal vesicle (gv).

Primary OSC cultures can be trypsinized and stored frozen for additional utilization. Figures 15A-C show images from time lapse cinematography of oocyte development in secondary OSC culture, beginning four hours after seeding (F36, 36 year old woman - for the OSC presence in the ovarian biopsy see Chapter 10.7). An early developing cell (Figure 15A, time 1 min, 15 seconds of cinematography) shows a cytoplasmic tail (arrowhead). At 18 min, seven seconds multiple cytoplasmic eruptions (arrowheads) are evident (Figure 15B). At 31 min, 14 seconds the 40 micrometers oocyte-like cell developed (Figure 15C, yellow arrowheads indicate a cell surface, red arrowhead a polar body). This video is available as Additional file 1, supplemental video S1.

Panels 15D-F show a secondary OSC culture from a 55 year old postmenopausal women (for OSC in the ovarian biopsy see Chapter 10.7) stained for ZP afer three days of seeding (d3sc ZP). Oocyte precursors can produce, by several divisions, a "chain" of satellite cells. Such a chain is presented in Figure 15D. Note that cells in the chain are interconnected by intercellular bridges lacking division by cytoplasmic membranes (open white arrowheads). A membrane with ZP expression is apparent on their surface (open black arrowheads).

The last cell in the chain represents satellite remnants (sr). Regressing satellites (rs) follow, and a large satellite (s) is close to the developing oocyte (o) leading the chain. The developing oocyte shows formation of the cell surface in the bridge (solid yellow arrowhead). An asterisk indicates the leading oocyte nucleus and the red arrowhead a developing polar body. Figure 15E shows two "leading" oocyte type cells with surface ZP expression, each of which shows expulsion of two polar bodies (red arrowheads), retention of two pronuclei (asterisks), and surface ZP expression (open black arrowheads). Yellow arrowheads indicate a line of separation from a regressing satellite (rs) and satellite remnants (sr) lacking surface ZP. A large oocyte ( micrometers) with a nucleus (asterisk), germinal vesicle (gv), and surface ZP expression (open black arrowhead) is shown in Figure 15F.

These observations resemble the formation and fate of *Drosophila melanogaster* ovarian cysts (egg chambers). In Drosophila, ovarian cysts are produced through a series of synchronous mitotic divisions during which cytokinesis is not completed. After completion of four mitotic divisions, all 16 cells enter the premeiotic S phase. However, only the true oocyte at the center of the syncytium remains in meiosis and continues to develop. The remaining 15 cells lose their meiotic features and develop as polyploid nurse cells. In contrast to nurse cells, the oocyte remains in prophase of meiosis I until it proceeds to the first meiotic metaphase late in oogenesis. The oocyte fate is dependent on the unique presence of the *missing oocyte* (*mio*) gene, which is required for the maintenance of the meiotic cycle and oocyte identity. *Mio* associates with the conserved nucleoporin *seh1*, which influences the oocyte development. In drosophila females lacking *seh1* about 20% of egg chambers develop with 16 polyploid nurse cells and no oocyte (for data and review see Ref. [180]). Oocyte syncytia (ovarian cysts) also develop in mouse and human fetal ovaries [112,187,188].

#### 10.4.3. Ovarian stem cell cultures vs. in vivo oocyte and follicular development in mammals

The behavior of developing oocytes in human OSC cultures resembles their behavior in ovarian cysts, from adult Drosophila to mouse and human fetuses. In other words, the oocyte development from human OSC *in vitro* may utilize the *mio* gene and nucleoporin *seh1* mechanisms to produce own nurse (satellite) cells, thereby preventing them from developing into additional oocytes. Since some *in vitro* developed oocytes continue to differentiate into parthenotic embryos (Figure 12D and E, and below), like some cultured follicular oocytes, the *in vitro* developed oocytes are functionally competent.

In adult inverterbrates and lower vertebrates (fish, amphibia), all periodically developed oocytes are ovulated. In higher vertebrates, however, each oocyte lies in the ovarian follicle, where its further development is dependent on the activity of granulosa cells providing additional organelles required by growing oocytes (Balbiani body). The activity of granulosa cells is regulated by the follicular TCS niche (see Figure 11A-D). In addition, the selection of dominant follicle(s) (Figure 11E-J) ensures the number of ovulations in a species specific manner, which is optimal for the maintenance of the certain number of fetuses during pregnancy. Therefore, once granulosa cells are available during ontogeny, the development of oocytes into ovarian cysts with nurse cells is switched into follicular development (Figures 2, 3 and 4). *In vitro*, the follicles with granulosa cells and the TCS niche are absent, so oocytes developing from OSC utilize the developmentally primitive mechanism of ovarian cysts formed by the oocyte and its nurse cells.

#### 10.4.4. A comparison of the primary vs. secondary ovarian stem cell cultures

Our observations indicate that the behavior of primary and secondary OSC cultures are different. The primary OSC cultures contain ovarian stromal cells, which contribute to oocyte growth and development. Such oocytes show the ability to develop into parthenotes, similarly to some follicular oocytes during standard IVF techniques. This suggests that *in vitro* developed oocytes are in principle similar to the cultured follicular oocytes. The lack of expression of ZP proteins at the developed oocyte surface will, however, require ICSI for their fertilization, unless they are stimulated to express ZP by granulosa cells from primary OSC cultures without estrogenic stimulation. Secondary OSC cultures utilize a developmentally ancient mechanism known from the Drosophila ovaries. They produce their own satellites for the oocyte maturation and mature oocytes express ZP proteins. This may be useful for standard oocyte fertilization. Yet, the targeted ICSI may still be found useful for the fertilization of the selected oocytes.

### 10.5. Development of embryonic stem cells from in vitro developed parthenotes

**Figure 16:**
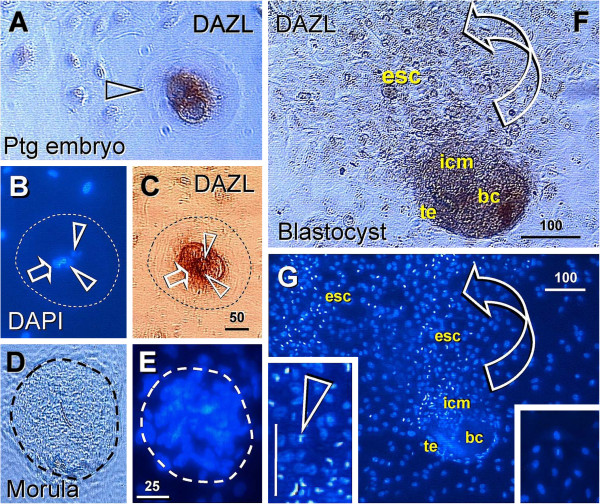
**Parthenotes in OSC cultures. A-C**) Four cell embryo. **D **and **E**) morula. **F** and **G**) Blastocyst consisting of blastocoele (bc), trophectoderm (te) and inner cell mass (icm) releasing (arched arrow) DAZL+ embryonic stem cells (esc). Scale in C for A-C. A, C and F - Dazl staining, B, E and G - DAPI. Adapted with permission from [179], © Cambridge University Press.

DAZL protein, expressed in human oocytes, preimplantation embryos, and ESC [106], is strongly expressed in early (four cell stage) parthenotes (Figure 16A-C). Resulting morulae (Figure 16D and E, no immunohistochemistry) may develop into blastocysts producing `DAZL^+^ ESC from the embryonic inner cell mass into the culture (arched arrow and esc, Figure 16F). The inner cell mass and the released ESC are mitotically active compared to the other cells lacking DAZL expression and by pronounced DAPI staining (arrowhead in left vs. no mitoses in the right inset, Figure 16G) [179].

These observations indicate that OSC cultures could be sources of eggs for the treatment of female ovarian infertility, and can also produce ESC for autologous regenerative medicine.

### 10.6. Development of oocytes from postmenopausal and POF ovaries

Ovarian cultures derived from atrophic postmenopausal ovaries are capable of producing new oocytes, some of which differentiate into parthenogenetic morulae, suggesting their functional potential. Apparently, the OSC may differentiate and proliferate during advanced postmenopause [134,178] and in young women with POF [94,189-191].

### 10.7. Cultures from ovaries lacking ovarian stem cells fail to produce oocytes

**Figure 17:**
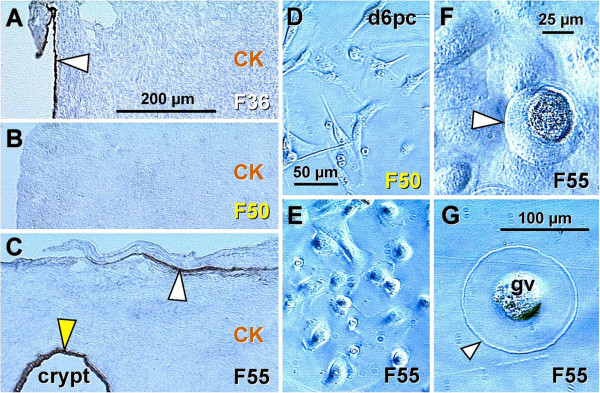
**Influence of the presence vs. absence of OSC in the ovary on ovarian cultures. **Occurrence of OSC in pre- and postmenopausal ovaries (**A-C**); note a lack of adult primordial follicles in all samples. **A**) Staining for CK with hematoxylin counterstain shows CK+ OSC (arrowhead) in the ovary of 36 years old woman (F36). **B**) Ovary of 50 years old women without OSC. **C**) Activity of the TA (white arrowhead) with OSC in 55 year old ovary. The OSC is also present in the cortical crypt (yellow arrowhead) in the deeper cortex. (**D-G**) Phase contrast from live day 6 primary ovarian cultures (d6pc). **D**) Ovarian culture of a 50 year old female shows only narrow fibroblasts (note lack of OSC in panel **B**). **E**) Cluster of epithelial type cells in culture from a 55 year old female (note OSC in panel **C**). **F**) Detail from another cluster of epithelial cells shows small (30 micrometers) round oocyte type cell (arrowhead) with a prominent nucleus. **G**) Large (120 micrometers) round oocyte type cell with germinal vesicle (gv) and thickened plasma membrane (arrowhead).

The presence of OSC in ovaries has been found essential for the development of oocytes in ovarian cultures. Figure 17A shows CK+ OSC (arrowhead) in the ovary of a 36-year old woman (F36). However, perimenopausal ovaries (around 50 years of age, ages 49–52 in our observations - see below) often lacked OSC (Figure 17B), as well as some ovaries (one of three cases) of women with POF [94]. On the other hand, women in advanced menopause exhibited OSC regularly [134]. Figure 17C shows the ovary of a 55-year old women exhibiting CK+ OSC on the ovarian surface (white arrowhead). Ovarian stem cells are also present in the cortical crypt in the deeper cortex (yellow arrowhead). Ovarian culture of the 50-year old female (Figure 17D) showed only narrow fibroblasts. Note a lack of OSC in Figure 17B. A cluster of epithelial type cells was observed in culture from the 55-year old postmenopausal female (Figure 17E). Note OSC in Figure 17C. Detail from another cluster of epithelial cells showed a 30 micrometer round oocyte-type cell (arrowhead, Figure 17F) with a prominent nucleus. A large (120 micrometers) round oocyte type cell with a germinal vesicle (gv) and thickened plasma membrane (arrowhead) was also detected (Figure 17G).

These observations show that ovaries lacking OSC are unable to produce oocytes in ovarian cultures. The presence of OSC is essential for *in vitro* oocyte development. The OSC usually persist in postmenopausal ovaries, serving as a source for development of epithelial cell clusters in ovarian cultures. Some of these OSC-derived epithelial cells appear to be precursors of gradually differentiating oocytes. Spontaneous development of parthenogenetic embryos in ovarian cultures, which can also be observed during oocyte handling for standard IVF purposes [186], indicates the functional similarity of *in vitro* developed eggs with cultured follicular oocytes. Development of oocytes from OSC derived from ovaries with no naturally present follicles and oocytes (POF and postmenopausal women) [94,189] confirms the ability of OSC to produce new eggs in the absence of OSC niche present during neo-oogenesis *in vivo*.

The observations reported above and arranged according to the patient's age are summarized in Table 2. There were three POF cases, out of which two cases (#1 and #3) exhibited OSC and exhibited oogenesis *in vitro*. No oocytes in culture were observed in case #2 lacking OSC in their ovaries. Three perimenopausal women (ages 49–52) lacked OSC in the ovaries and produced no oocytes *in vitro*. It is possible that during the perimenopausal period altered immune physiology causes an alteration of the OSC differentiation from TA precursors.

## 11. Oocyte formation by mitotically active germ cells purified from ovaries of reproductive-age women

### 11.1. Repowering the ovary

In a recent article in Science–Business eXchange (SciBX) of the Nature Publishing Group [104], the Senior Editor Tracey Baas indicated:

“Up until the 1990s, the central dogma of reproductive biology was that female mammals have a restricted capacity for generating oocytes before birth, and once born the ovaries cannot renew egg cells that die because of aging or disease. Consequently, infertility resulting from oocyte loss had been considered irreversible.

However, multiple papers now cast doubt on that belief through the identification of a population of stem cells that give rise to functional oocytes.

First, Antonin Bukovsky and colleagues at The University of Tennessee Knoxville published in the American Journal of Reproductive Immunology in 1995 that a subpopulation of human germline stem cells, now known as oogonial stem cells (OSCs), could be collected from the ovaries of women undergoing surgery and used to generate what were perceived as oocytes in cell culture, based on detection of oocyte markers [7,11].

Almost a decade later, Jonathan Tilly and colleagues at Massachusetts General Hospital (MGH) and Harvard Medical School (HMS) produced multiple datasets that ran counter to the belief that germline stem cells disappear from ovaries at birth [91].

In 2009, Ji Wu and colleagues at Shanghai Jiao Tong University used a unique biomarker of murine OSCs—dubbed DEAD box polypeptide 4 (Ddx4)—to isolate OSCs from adult mouse ovaries. The team used the marker to purify OSCs, which were transplanted into ovaries of infertile mice to generate functional oocytes capable of producing offspring .

Those data were published in Nature Cell Biology. The open question was whether OSCs purified from humans had a similar ability to generate functional ovarian follicles.

To answer that question, researchers at MGH and HMS started by modifying Wu’s Ddx4-based purification protocol to improve its selectivity for OSCs. The team, which was once again led by Tilly, used fluorescence-activated cell sorting (FACS), which is less likely to be contaminated by oocytes than Wu’s technique of magnetic bead sorting.

His group isolated DDX4-positive cells from murine ovarian tissue as well as from human ovarian tissue derived from whole ovaries of reproductive-age women undergoing elective surgery. *In vitro*, both human and murine DDX4-positive cells expressed primitive germline markers but not oocyte markers, suggesting the procedure had indeed purified OSCs. To determine whether the OSCs could differentiate into viable oocytes, the team cultured the cells on mouse embryo fibroblasts used as feeder cells, which supplied a cellular matrix upon which the stem cells grew. The human and mouse OSCs spontaneously differentiated into oocytes, as assessed by morphology, gene expression patterns and progression through meiosis.

The next step was to look at whether the OSCs could generate oocytes *in vivo*. To do so, the team engineered the mouse and human OSCs to express GFP, which made it possible to visualize and track the cells. When GFP-expressing mouse OSCs were injected into the ovaries of mice, primary ovarian follicles developed that contained GFP-positive oocytes. Moreover, those oocytes led to the development of GFP expressing mouse embryos following fertilization.

Finally, GFP-expressing human OSCs were injected into human ovarian tissue and transplanted into immunodeficient mice. The result was primary ovarian follicles that contained GFP-expressing oocytes.

That suggested OSCs might indeed give rise to functional oocytes if transplanted into humans.

Results were published in Nature Medicine [103]. The team also included researchers from Saitama Medical University. Tilly and colleagues are now optimizing the conditions for production of human oocytes *in vitro* and have started studies to assess whether OSCs in nonhuman primates can generate functional oocytes for IVF.

Bukovsky, professor of reproductive biology associated with the Institute of Biotechnology of the Academy of Sciences of the Czech Republic, said he is most interested in seeing the work translated from ovarian tissue obtained from reproductive-aged women to women with ovarian failure who are unable to produce follicle cells or oocytes. “Tilly’s work supports the existing idea that purified germ cells transplanted into mouse ovaries will utilize existing immature follicle cells to produce new follicles and that the survival and function of oocytes in vivo requires interaction with these immature follicle cells,” he said. “It will be very important that the team reproduce the experiments using ovarian tissue biopsies obtained from reproductive-aged women and from women with premature ovarian failure. I would very much like to see the mechanistic details”.

Questions aside, Oktay said the new study is “a game changer because it increases the options available for obtaining viable eggs. When it comes to fertility restoration, it is all about options”.

Wu, professor of molecular reproduction and stem cell biology at the Bio-X Center of Shanghai Jiao Tong University, thinks there are still unaddressed technical challenges. “It is very satisfying to see our protocol optimized and fine-tuned to obtain and differentiate human female germline stem cells into oocytes, but the team’s culturing conditions will have to be optimized to avoid use of animal components, and mouse feeder cells will need to be replaced with either human feeder cells or a nonfeeder culturing system,” Wu told SciBX [104]”.

For complete SciBX article see Additional file 2, Supplemental material.

The article published in Nature Medicine [103] indicates that primitive germ cells purified from the cortex of functional adult human ovaries form new ovarian follicles when injected into human ovarian cortical biopsies and xeno-transplanted into immunodeficient NOD-SCID mice. The article is important, since it is the first confirmation of former observations on neo-oogenesis and follicular renewal in adult human ovaries published in 1995 [87] and expanded thereafter [7,11,68,94,134,135,139,192]. In addition, neo-oogenesis from secondary germ cells is already present in human fetal ovaries, during the second trimester of pregnancy [9,100].

It has to be noted, that the earlier rarely used term “oogonial stem cells” [193-195], now considered as a new term [103], is doubtful, since such cells are in reality well known germ cells capable to proliferate, originating in fetal and adult human ovaries by asymmetric division of OSC progenitor cells (see Chapters 4-6), and not persisting in adult ovaries from the fetal period.

In a recent commentary [10], Telfer and Albertini indicated:

“Work of White et al. [103] represents an advance that has the potential to change the nature of future infertility treatments, although many practical and conceptual obstacles remain before the clinical utility of their methods can be realized. Much effort will be required to improve the efficiency of isolation and transformation of OSCs into oocytes, as the number of OSCs that went on to form follicle-enclosed oocytes in the study by White et al. was small.”

Even in the case of the mouse studies, very few GFP-positive oocytes were shown to fertilize and undergo even minimal embryogenesis, with many of these oocytes clearly arresting at the preblastocyst stage. Further, a more detailed characterization of genetic integrity (euploidy and the appropriate retention of epigenetic marks) and other hallmarks of oocyte quality (such as meiotic and developmental competence) are required before any clinical application of these techniques can be considered.

Given the response to earlier work from this laboratory, questions will undoubtedly linger as to whether these cells are only activated *in vitro* or whether they indeed contribute to de novo neo-oogenesis *in vivo*. Further research will be required before these issues can be fully resolved. Nonetheless, the findings of this study will change the tone of future discourse on the subject toward measured enthusiasm and, most importantly, will prompt speculation and tempered progress into what remains a major obstacle in the treatment of various forms of human infertility [10]”.

In an additional commentary [196] Oatley and Hunt indicated:

“For OSCs, doubt will persist until clear evidence is provided that they give rise to genetically normal, developmentally competent eggs. In the meantime, skeptics are plagued by several nagging questions: What do these cells do in the ovary? Where do they come from? And, most importantly, if they can and do give rise to viable eggs in the adult ovary, why is female reproduction of such limited duration? [195].”

### 11.2. The importance of the presence of uncommitted granulosa cell nests for the preservation and development of transplanted primitive germ cells

It has been shown that the survival and function of oocytes *in vivo* require their interaction with granulosa cells, and the number or activity of granulosa cells may restrict function of female germ cells [11,196]. In human ovaries, follicular renewal is initiated by the formation of granulosa cell nests from the bipotent TA precursors, and they are transported into the deep ovarian cortex [11,87] (Figure 6). Germ cells subsequently develop by asymmetric division of the OSC (see Figure 8A and B), symmetrically divide in the TA (Figure 8C), migrate from the TA into the upper ovarian cortex (Figure 8C and D), where they associate with and enter cortical blood venules (Figure 8E and F). Some of the circulating germ cells are captured by uncommitted granulosa cell nests associated with ovarian vasculature in the deep ovarian cortex (Figure 7B and 9C). Once committed, the granulosa cell nests form a Balbiani body in the ooplasm (asterisk, Figure 7C) and a new "primordial" follicle (Figure 7D). Germ cells originating from the OSC crypts in the deep ovarian cortex migrate toward neighboring uncommited granulosa cell nests to form new follicles (Figure 7G and H), or enter ovarian vasculature to circulate and search for uncommitted nests within vessels.

### 11.3. A lack of uncommitted granulosa cell nests causes a degeneration of the germ cells

**Figure 18:**
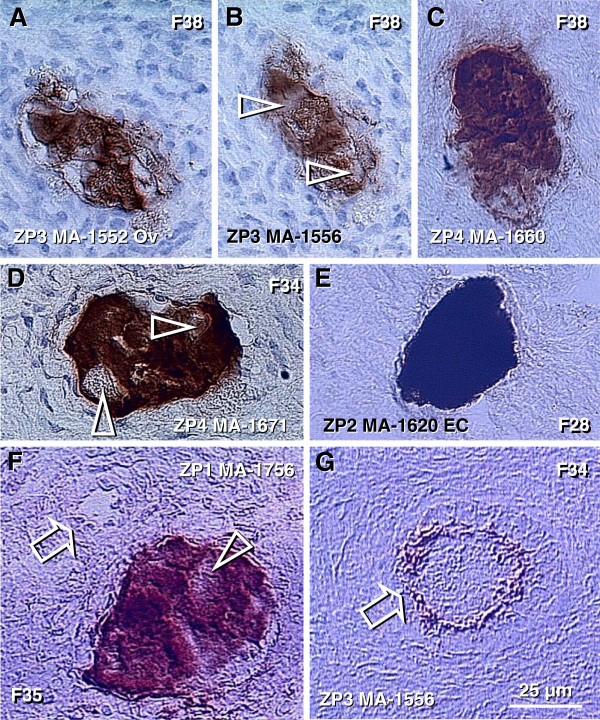
**Intravascular degenerating human oocytes. **Degenerating oocytes in venules of ovarian medulla (**A D **and **F**) and uterine endocervical stroma (**E**) expressing zona pellucida proteins identified by MAb clones against ZP3, ZP4 and ZP 2 [198] as indicated in panels. MAb to ZP1 was kindly provided by Dr. Satish K Gupta. Arrowheads indicate unstained oocyte nuclei, arrow in F shows an arteriole, arrow in G shows ZP expression at the oocyte surface in a normal secondary follicle. F28-F38 indicate patients’ ages. Panels A and C adapted from [139] with permission, © Elsevier.

If or when such nests are not available, the remaining germ cells within several days increase to the 50 micrometers oocyte size, show profound cytoplasmic ZP expression, and degenerate, either within the ovarian medullary vessels (Figure 7I, and Figure 18A-D and F) or elsewhere else (Figure 18E). Figure 18G shows the ZP expression limited to the oocyte surface in a normal secondary follicle.

The availability of transplanted germ cells in adult human ovaries lacking uncommitted granulosa cell nests may not be sufficient for follicular renewal [192]. Unlike mice, adult women with ovarian failure do not exhibit uncommitted granulosa cell nests [94,199] compared to women with follicular renewal [11,87].

### 11.4. Alternative approaches for the treatment of ovarian infertility

An alternative approach for human females with ovarian infertility, most of which exhibit OSC regardless of their age, is the *in vitro* development of new oocytes from OSC in the presence of ovarian stromal cells [7]. Ovarian stromal cells are capable of producing androgens and estrogens [180,181], which accelerate oocyte formation from OSC *in vitro*[7]. In addition, the ovarian stromal cells neighboring the oocyte are transformed into satellite cells and substitute granulosa cell-derived Balbiani bodies to provide additional organelles required by the growing oocyte. Such oocytes are capable differentiating and maturing [9]. They must be fertilized [94] by intracytoplasmic sperm injection, as they have no significant surface expression of ZP proteins [179] (ZP, Figure 12A). *In vitro* developed embryos tested for normalcy can be utilized for assisted reproductive technology.

### 11.5. Restoration of the OSC niche after chemotherapy

Ovarian infertility is caused by alteration of the OSC niche normally consisting of vascular pericytes, immune system-related cells sensitive to chemotherapy (monocyte-derived cells and particularly CD8+ T cells), and autonomic innervation [1,9]. In cancer patients, it should be possible to collect bone marrow or circulating white blood cells prior to chemotherapy, and return them thereafter back to the patient to restore the OSC niche [200].

## 12. Neo-oogenesis *in vitro* vs. conventional IVF

Previous *in vitro* observations of other investigators, including IVF practices, dealt with established follicular oocytes collected from mature ovarian follicles. Follicular oocytes show a uniform pattern, since they have already accumulated organelles supplied by granulosa cells (Balbiani body) and a thick ZP membrane. Cultures of follicular oocytes do not contain satellite cells, which appear to be involved in the stimulation of oocyte development in OSC cultures [7,179]. In contrast, oocytes evolving *de novo* in ovarian cultures show a different morphology during sequential stages of development which cannot be observed in follicular oocyte cultures.

Conventional IVF with autologous follicular oocytes is usually not used in women over 40 years of age, due to failure rate of implantation as well as the increasing incidence of fetal abnormalities. The live birth rates in IVF under age 31 are 66-74%, and decline thereafter, being 18-27% for ages 41 and 42, and 6-11% for age 43 or higher, reportedly due to the diminished ovarian reserve, since the rate is 60-80% for all recipient ages using donor eggs [201]. In contrast fresh oocytes develop *de novo* from OSC in culture regardless of the female age. Therefore, *in vitro* newly developed oocytes could be utilized for IVF in women exceeding 40 years of age with improved success rates and theoretically a low incidence of fetal abnormalities vs. follicular oocytes collected from ovaries of aging females (see Figure 1).

More importantly, conventional IVF and assisted reproduction technique (ART) can never accomplish the provision of genetically related children in women lacking the ability to produce their own follicular oocytes, including young women rendered sterile after chemotherapy associated with oocyte loss and women with POF. Autologous oocytes from OSC in culture have been shown to produce blastocysts after fertilization [202], and therefore could be used in subsequent IVF and ART procedures.

## 13. Why does menopause occur?

As indicated above, Ji Wu and colleagues have shown that ovarian germline stem cell cultures derived from 5 day old and adult mouse ovaries infected with GFP transgene produced functional oocytes when transplanted into ovaries of infertile mice. Such oocytes produced offspring expressing GFP of transplanted germline stem cells [102]. The article confirms that OSC have the capacity to produce meiotically potent and functional oocytes in infertile mouse ovaries. This is also supported by the human OSC culture studies, where some *in vitro* developing oocytes differentiate into parthenogenetic embryos [72,179,189,190].

### 13.1. A physiological role of ovarian stem cells in normal ovaries

In a subsequent commentary [197] to the article of Ji Wu and colleagues [102], several important questions were raised regarding the *in-vivo* biology for ovarian function and the relevance of this work to reproductive health in women. Is there a physiological role of OSC in normal ovaries? It has been suggested that although these stem cells originated from normal ovaries, their full germline potential may be the consequence of long term culture and their oogenic activity normally suppressed *in vivo*.

Regarding the oogenic activity of human OSC in normal ovaries, it is undoubtedly suppressed unless two conditions are met (see Figure 9). Firstly, there should be certain hormonal conditions accompanying the fetal midgestation and adult periovulatory periods, such as high circulating levels of estradiol and LH/hCG. Under these conditions, however, only some ovarian stem cells are converted into germ cells *in vivo* by asymmetric division due to the requirement of local cellular signaling by immune system-related cells [68,134], (so-called immune physiology of the mammalian ovary [203] or immunoregulation of ovarian homeostasis [69]). Immunohistochemistry of the immune system-related cells (T cells and MDC) has shown that the emergence of germ cells from OSC *in vivo* requires primitive MDC (CD14+) and activated (HLA-DR+) CD8+ T cells. The emergence of granulosa cells is accompanied by activated (HLA-DR+) MDC [87,100,203]. Also, during adulthood, activated MDC accompanies migration of germ cells from TA to the ovarian cortex, where they enter ovarian vessels 11,87,100,203] (see Figure 8).

Transplantation of bone marrow cells restores fertility in mice after chemotherapy-induced POF [204], possibly due to the addition of immune system-related cells. It appears, however, that the immune system-related cells are not required for oogenesis from OSC *in vitro*. Hence oogenic activity of ovarian stem cells seems to be inhibited *in vivo*, unless appropriate hormonal and cellular signals occur, but this may not be required *in vitro* (see Chapter 10).

### 13.2. Availability of granulosa cells

Survival and function of oocytes *in vivo* requires their interaction with granulosa cells, and the number or activity of granulosa cells may restrict the function of female germ cells [197]. Regarding granulosa cells, marked differences exist between adult ovaries in different species. In small laboratory rodents, granulosa cells originate from solid epithelial cords [3]. The ovarian structure resembles human fetal ovaries with an abundance of OSC-derived granulosa cells [100,135]. However, in adult human ovaries during the prime reproductive period the development of OSC-derived cortical nests of primitive granulosa cells occurs [122,205]. Granulosa cell nests are transported through dense ovarian stroma to the deep cortex to assemble with newly formed oocytes provided by vascular transport or migration from the adjacent cortical OSC crypt [11]. Our observations also show that the number of newly formed adult primordial follicles is determined by the availability of granulosa cell nests. Superfluous vascular oocytes degenerate in the medullary venules [11,87,139].

After the prime reproductive period and in ovaries with POF, however, degenerating intravascular oocytes and ovarian cortical granulosa cell nests are virtually absent [94,134]. This indicates that the availability of transplanted germ cells in adult human ovaries lacking granulosa cell nests may not be sufficient for follicular renewal. Alternatively, transplantation of uncommitted autologous OSC may be a source of both cell types required for follicular renewal, the granulosa cell nests and germ cells. Furthermore, transplantation of autologous committed germ cells may be accompanied by uncommitted autologous OSC, if available. Cultured human OSC could be frozen for an extended storage and future use [202].

The restoration of ovarian function in ovaries with artificially depleted oocytes [102] was successful probably due to the preservation of the mouse ovarian stem cell niche and the preserved granulosa cells. However, POF in human females is supposedly caused by the programmed premature termination of the ovarian stem cell niche function [9]. Many of such ovaries still carry OSC but fail to renew their missing primordial follicles. Mature oocytes can, however, be produced by OSC culture [12].

### 13.3. Why do ovarian stem cells not prevent menopause?

Another important query raised was that even if the activity of ovarian stem cells *in vivo* is shown to replenish follicular pool and is accepted, why do they fail to maintain ovarian function with advancing age [197]. Indeed, OSC from anovulatory and postmenopausal ovaries have the capacity to differentiate *in vitro* into oocytes [94]. However, it has to be taken into account that the immune system shows a significant functional decline between 35 and 40 years of age [150] and concomitantly the ovarian follicular renewal ceases. Continuation of ovarian function until the menopause is based on utilization of aged primordial follicles previously formed [11]. The age associated changes in the immune system may be responsible for the termination of neo-oogenesis and follicular renewal *in vivo*[87,132].

During development in human females, germ cells differentiate much earlier during the embryonic period, as compared to granulosa cells and fetal primordial follicles, which appear during the second trimester of fetal intrauterine life [112]. There is a striking correlation between the period at which organ components are present during early ontogeny and that organ's functional longevity [1]. It is likely that the lack of formation of granulosa cells required for the formation of new primordial follicles and the resulting cessation of ovarian ovulatory function is central to the occurrence of the menopause.

### 13.4. Perimenopausal disorders

During the perimenopausal period the risk of acute myocardial infarction rises sharply, as well as hypertension and increased lipids and body weight, vascular intima-media thickness, breast cancer risk, and aging brain disorders, especially dementia and Parkinson's disease [206,207]. Differentiation of OSC is dependent on the TCS ensuring tissue homeostasis. The lack of OSC differentiation around 50 years of age (Table 2) suggest that homeostasis is particularly altered at that time in order to terminate any ovarian activity. Such perimenopausal alteration of homeostasis may also induce disorders of other tissues listed above.

## 14. Clinical trial

This chapter is an adapted version published as a "Potential treatment of ovarian infertility" part of the Ref. [94].

### 14.1. Differentiation of oocytes from OSC in vitro

Early in 2005, we established primary OSC cultures and observed that OSC have a capacity to differentiate into distinct somatic cell types (epithelial cells, fibroblasts, granulosa, and neural type cells) and also oocytes [7]. The functional capacity of such OSC-derived oocytes is confirmed by their development into parthenogenetic embryos expressing DAZL protein - see Figure 16. Although OSC originate from fetal mesothelial cells covering peritoneal cavity, the adult peritoneal mesothelial cells do not have characteristics of stem cells as they persist unchanged in culture [94]. Therefore, OSC may represent a new adult stem cell type with unique totipotent features.

### 14.2. Potential treatment of ovarian infertility

The criteria for the utilization of OSC cultures in the treatment of the female ovarian infertility was elaborated [12] as follows.

### 14.3. Suitability of patients for clinical trial

Patients with the diagnosis of premature ovarian failure (POF) may be included in the clinical trial. Optimally, these patients failed to conceive due to a lack of their own functional oocytes during previous standard IVF therapy, or such therapy was impossible due to the lack of oocytes within ovaries, and they are considering new options to have a genetically related child before using donated oocytes. Patients should provide a detailed medical history and available laboratory results for consideration in the trial. Ultrasound or MRI images of ovaries should be done, and patients advised to utilize certain hormonal therapies several weeks prior to the procedure.

Prospective patients and their partners should not carry any genetical alterations that can be transmitted to the child. Of particular importance is the exclusion of POF with the fragile X premutation (>200 CGG trinucleotide repeats of FMR1 gene), since the birth of a child in such women may result in mental retardation of the progeny [208]. Genetic alterations are detected in a proportion of patients with POF, particularly those with primary amenorrhea [157], and fragile X premutation was detected in 4.8% of patients with POF [209]. Therefore, evidence on the lack of a known genetic abnormality should be provided, or the patients tested.

If needed, additional laboratory investigation from blood and urine, as well as imaging procedures would be done after admission. All considered women should have a male partner with normal semen quality. Women with infertile partners (i.e. with azoospermia) should be excluded.

Therapy of ovarian infertility with cultured OSC should be explained to the patient by a specialist in gynecology and obstetrics, who is familiar with this new technique. The medical documentation of each patient and her male partner should be evaluated by an interdisciplinary committee for *in vitro* fertilization, which would approve inclusion into the trial. An institutional review board (IRB) should approve the clinical trial.

### 14.4. Collection of ovarian stem cells and in vitro culture of oocytes

Ovarian stem cells and small ovarian biopsies are collected during laparoscopy. OSC and cells collected by scraping of tissue biopsies are cultured for 5 to 10 days to determine whether or not they can produce oocytes. If oocytes develop, they can be genetically analyzed. In the future, they may be fertilized by classical IVF, or by ICSI with the partner’s semen after approval of the Medical Ethics Committee. Embryos, if developed, are cultured to the blastocyst stage, and before transfer into the uterus, evaluated by preimplantation genetic diagnosis. Embryos may be cryopreserved. When a woman is hormonally prepared, at most two normal blastocysts can be transferred into the uterus and supernumerary blastocysts are cryopreserved for a potential later need of the patient. In case of a pregnancy, amniocentesis should be performed for genetic evaluation of the fetus.

### 14.5. Potential pitfalls

During the clinical trial, the following complications of cultured cells could occur: oocytes could not develop, oocytes could not be appropriate for fertilization, oocytes could not be fertilized, fertilized oocytes could not develop into embryos, or embryos could not be transferred into the uterus because they were genetically abnormal.

### 14.6. Initiation of the first clinical trial

Criteria for initiation of a clinical trial was found appropriate and its initiation in the IVF laboratory, Department of Obstetrics and Gynecology, University Medical Center Ljubljana, approved by the Slovenian Committee for the Medical Ethics. Early in 2006, Dr. Antonin Bukovsky (AB) and Dr. Irma Virant-Klun (IVK) met in her IVF laboratory of the Department of Obstetrics and Gynecology, University Medical Centre Ljubljana, Slovenia, to initiate the trial. The objective was to evaluate if there are OSC in infertile women with POF, if they contain putative stem cells, and if they can develop into oocytes capable of fertilization *in vitro*. After informed consent process, three patients with POF and no naturally present oocytes in ovaries, aged 30, 38, and 40 years, and their normospermic partners were selected.

**Figure 19:**
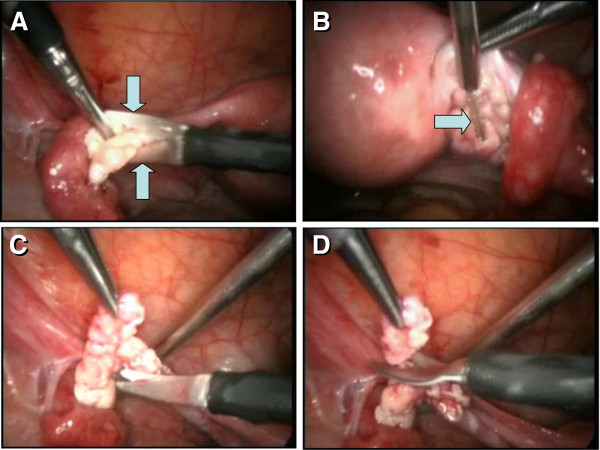
**Snapshots from the collection of the OSC and ovarian biopsy from POF ovary by a laparoscopy.** OSC are collected by scratching the ovarian surface with scissors (arrows in **A**) and with a brush (arrow in **B**)**. **Ovarian biopsies (**C** and **D**) are collected from each ovary. For a complete video see Additional file 3, Video S2.

Collection of the OSC was performed by Dr. Andrej Vogler of the Department of Obstetrics and Gynecology, University Medical Centre Ljubljana, 1000 Ljubljana, Slovenia, in collaboration with AB and IVK. The cells were collected during diagnostic laparoscopy by scratching the ovarian surface with scissors (Figure 19A) and brush (Figure 19B), and ovarian biopsies (Figure 19 C and D) were collected from both ovaries (see also Additional file 3, supplemental video S2). The cells were collected from the scissors and the brush into the culture medium. Before the end of laparoscopy, the ovaries were washed in reverse Trendelenburg position with 37°C warm saline, and the liquid with cells was collected from the cul-de-sac space. The collected liquid was then spun down and cells in the pellet were dissolved in culture medium. From half of each biopsy and collected OSC, the cell cultures were set up in DMEM/F12 medium with phenol red (weak estrogenic action), supplemented with antibiotics and 20% comprehensively heat-inactivated serum (59°C, 60 minutes) of the corresponding patient. The culture was monitored daily.

Ovarian cells attached to the bottom of the dish began to differentiate into epithelial and fibroblast cell types, and some of them into oocytes. On day 3 of culture, the initial medium was replaced with *in vitro* maturation medium (Medicult IVM, Copenhagen, Denmark) supplemented with FSH (75mIU/ml), hCG (5 IU/ml) and 10% heat-inactivated patient's serum. Prepared male partner's sperm were added several hours later. Embryo-like structures developed in the OSC cultures of two POF women on the next day. They detached spontaneously and were transferred into the wells with standard medium for *in vitro* fertilization, where they developed progressively to the morula-, preblastocyst- and blastocyst-like structures. They were frozen to be later genetically analyzed and transferred into the uterus, if normal. Oocytes developed *in vitro* were analyzed genetically and were shown to express genes related to oocytes and pluripotent stem cells.

The remaining oocytes developed *in vitro* were analyzed genetically and were shown to express genes related to oocytes and pluripotent stem cells.

Material from biopsies was investigated by immunohistochemistry for the presence of OSC and granulosa cells of primary follicles (cytokeratin expression). No primary or other follicle types were found. Development of oocytes and embryo-like structures after *in vitro* insemination of cultures correlated with the presence of OSC in the biopsies. In one woman with no OSC in both biopsies no oocytes developed and embryo-like structures were absent after utilization of *in vitro* maturation and sperm [94].

Results of this research confirm the presence of OSC stem cells in some infertile women with POF, which are capable of developing into oocytes and be fertilized *in vitro*. These observations indicate that adult human ovaries are capable of producing new oocytes for follicular renewal. This fails to occur *in vivo* in POF patients. Ovarian stem cultures offer a new chance for infertile women with POF to have genetically related offspring, and should be investigated further.

## 15. Conclusions

In addition to its classically defined role in host defenses, the immune system is integral to the differentiation and maintenance of all normal tissues. Function of distinct tissues during adulthood, including the ovary, requires (1) Renewal from stem cells, (2) Preservation of tissue-specific cells in a proper differentiated state, and (3) Regulation of tissue quantity. Such morphostasis can be executed by the TCS, consisting of immune system-related components, vascular pericytes and autonomic innervation. Morphostasis is established epigenetically, during morphogenetic developmental immune adaptation, i.e., during the critical developmental period. Subsequently, the tissues are maintained in a state of differentiation reached during the adaptation by a “stop effect” of resident and self renewing MDC. Alteration of certain tissue differentiation during the critical developmental period causes persistent alteration of that tissue function, including POF and primary amenorrhea.

Morphostasis is altered with advancing age, due to degenerative changes of the immune system. Immune cells and pericytes in fetal and adult human ovaries participate in: (1) follicular renewal during the prime reproductive period, which includes the origin of new germ cells by asymmetric division of OSC. This process is more complex (formation of primitive granulosa cell nests and vascular transport of new germ cells) than the formation of secondary germ cells and adult primordial follicles in fetal gonads, (2) Activated pericytes and MDC accompany selection of growing follicles from the adult primordial follicles present in ovaries, and (3) Temporary retardation of thecal development and selective support of granulosa cell maturation during large antral follicle selection are required for resistance to atresia.

Our studies have shown for the first time that in addition to neo-oogenesis and follicular renewal in human and rat females, human OSC are capable of differentiating into functional oocytes, as indicated by their ability to produce parthenogenetic embryos, including OSC from POF and postmenopausal ovaries. Conventional IVF with autologous follicular oocytes is usually not utilized in women exceeding 40 years of age, due to the high failure rate and fetal abnormalities. This is due to the cessation of oocyte and follicular renewal at 38+/-2 years of age. In contrast, however, functional oocytes develop *de novo* from OSC in culture regardless of age. Therefore, they can be used for IVF in women exceeding 40 years of age without a risk of high failure rates and high fetal abnormalities.

Most importantly, what conventional IVF and ART cannot accomplish is to provide genetically related children to women lacking the ability to produce their own follicular oocytes, including young women rendered sterile from chemotherapy associated with oocyte loss, and women with POF. This may be solved by clinical use of autologous oocytes from OSC in culture in the future.

Ovarian stem cells are present in premenopausal and postmenopausal women, with exception of the narrow perimenopausal period (ages 49–52). This may be caused by the overall alteration of tissue homeostasis causing sharply increased risk of acute myocardial infarction, hypertension, increased body weight, vascular disorders, breast cancer, and aging brain disorders.

## Abbreviations

ART: Assisted reproduction techniques; CK: Cytokeratin; CL: Corpora lutea; DAZL: Deleted azoospermia-like protein; DC: Dendritic cells; ESC: Embryonic stem cells; HLA-DR: Class II major histocompatibility antigens; IVF: *In-vitro* fertilization; MDC: Monocyte-derived cells; MHC-I: Major histocompatibility class I heavy chain antigens; OSC: Ovarian stem cells; OSCs: Oogonial stem cells; POF: Premature ovarian failure; SCP3: Synaptonemal complex protein-3; TA: Tunica albuginea; TCS: tissue control system; Thy-1: Thy-1 differentiation glycoprotein; ZP: Zona pellucida.

## Competing interests

AB and MRC are listed as inventors of the United States Patent No.: 8,232,077 B2 dated Jul. 31, 2012, entitled "Oocytes derived from ovarian culture initially containing no oocytes", and assigned to Ovacyte LLC (ovacyte.com).

## Authors’ contributions

AB has written the manuscript draft and MRC contributed to its final version. Both authors read and approved the final manuscript.

## Supplementary Material

Additional file 1 **Video S1. **Oocyte development after seeding of the secondary OSC culture. Click here for file

Additional file 2 **Supplemental material (S1). **Baas T: Repowering the ovary. Science-Business eXchange 2012, 5:4-6. Click here for file

Additional file 3 **Video S2. **Laparoscopic collection of OSC and biopsies from POF ovaries. Click here for file

## References

[B1] Bukovsky A Immune maintenance of self in morphostasis of distinct tissues, tumor growth, and regenerative medicine Scand J Immunol 2011 73 159 189 10.1111/j.1365-3083.2010.02497.x 21204896

[B2] Simkins CS Origin of the sex cells in man Am J Anat 1928 41 249 253 10.1002/aja.1000410205

[B3] Evans HM Swezy O Ovogenesis and the normal follicular cycle in adult mammalia Mem Univ Calif 1931 9 119 224 PMC1658094 18742022

[B4] Jirasek JE Paramesonephric epithelium and its relationship to the surface epithelium of the ovary Cesk Gynekol 1973 38 691 694 4765174

[B5] Bjersing L Cajander S Ovulation and the role of the ovarian surface epithelium Experientia 1975 31 605 608 10.1007/BF01932485 1140269

[B6] Auersperg N Siemens CH Myrdal SE Human ovarian surface epithelium in primary culture In Vitro 1984 20 743 755 10.1007/BF02618290 6083974

[B7] Bukovsky A Svetlikova M Caudle MR Oogenesis in cultures derived from adult human ovaries Reprod Biol Endocrinol 2005 3 17 http://www.rbej.com/content/3/1/17 10.1186/1477-7827-3-17 15871747 PMC1131924

[B8] Hayashi K Ogushi S Kurimoto K Shimamoto S Ohta H Saitou M Offspring from Oocytes Derived from in Vitro Primordial Germ Cell-Like Cells in Mice Science 2012 10.1126/science.1226889 23042295

[B9] Bukovsky A Ovarian stem cell niche and follicular renewal in mammals Anat Rec (Hoboken ) 2011 294 1284 1306 10.1002/ar.21422 21714105

[B10] Telfer EE Albertini DF The quest for human ovarian stem cells Nat Med 2012 18 353 354 10.1038/nm.2699 22395699

[B11] Bukovsky A Caudle MR Svetlikova M Upadhyaya NB Origin of germ cells and formation of new primary follicles in adult human ovaries Reprod Biol Endocrinol 2004 2 20 http://www.rbej.com/content/2/1/20 10.1186/1477-7827-2-20 15115550 PMC420494

[B12] Bukovsky A Copas P Virant-Klun I Potential new strategies for the treatment of ovarian infertility and degenerative diseases with autologous ovarian stem cells Expert Opin Biol Ther 2006 6 341 365 10.1517/14712598.6.4.341 16548762

[B13] Nishizuka Y Sakakura T Thymus and reproduction: sex-linked dysgenesia of the gonad after neonatal thymectomy in mice Science 1969 166 753 755 10.1126/science.166.3906.753 5823314

[B14] Nishizuka Y Sakakura T Ovarian dysgenesis induced by neonatal thymectomy in the mouse Endocrinology 1971 89 886 893 10.1210/endo-89-3-886 5566399

[B15] Sakakura T Nishizuka Y Thymic control mechanism in ovarian development: reconstitution of ovarian dysgenesis in thymectomized mice by replacement with thymic and other lymphoid tissues Endocrinology 1972 90 431 437 10.1210/endo-90-2-431 5009328

[B16] Sakakura T Nishizuka Y Thymic control mechanism in ovarian development: reconstitution of ovarian dysgenesis in thymectomized mice by replacement with thymic and other lymphoid tissues Endocrinology 1972 90 431 437 10.1210/endo-90-2-431 5009328

[B17] Kincl FA Oriol A Folch Pi A Maqueo M Prevention of steroid-induced sterility in neonatal rats with thymic cell suspension Proc Soc Exp Biol Med 1965 120 252 255

[B18] Russell WR Walpole AL Labhsetwar AP Cyclophosphamide: induction of superovulation in rats Nature 1973 241 129 130 10.1038/241129a0 4695540

[B19] Hahn EW Morales RL Superpregnancy following prefertilization X-irradiation of the rat J Reprod Fertil 1964 7 73 78 10.1530/jrf.0.0070073 14125187

[B20] Bukovsky A Presl J Krabec Z Dealyed anovulatory syndrome after long-lasting progesterone administration in early postnatal period in the rat and its relation to follicular atresia Cesk Gynekol 1976 41 281 285 949770

[B21] Bukovsky A Trebichavsky I Presl J Zidovsky J Submicroscopic evidence of lymphoid cells within the granulosa of the rat atretic follicle IRCS Med Sci 1978 6 80

[B22] Bukovsky A Presl J Zidovsky J Migration of lymphoid cells into the granulosa of rat ovarian follicles IRCS Med Sci 1979 7 603 604

[B23] Bukovsky A Presl J Holub M Ovarian morphology in congenitally athymic mice Folia Biol (Praha) 1978 24 442 443 729876

[B24] Bukovsky A Presl J Ovarian function and the immune system Med Hypotheses 1979 5 415 436 10.1016/0306-9877(79)90108-7 379555

[B25] Pate JL Toyokawa K Walusimbi S Brzezicka E The Interface of the Immune and Reproductive Systems in the Ovary: Lessons Learned from the Corpus Luteum of Domestic Animal Models Am J Reprod Immunol 2010 64 275 286 10.1111/j.1600-0897.2010.00906.x 20712810

[B26] Espey LL Ovulation as an inflammatory reaction–a hypothesis Biol Reprod 1980 22 73 106 10.1095/biolreprod22.1.73 6991013

[B27] Espey LL Current status of the hypothesis that mammalian ovulation is comparable to an inflammatory reaction Biol Reprod 1994 50 233 238 10.1095/biolreprod50.2.233 8142541

[B28] Klein J Immunology: The Science of Self-Nonself Discrimination 1982 New York: John Wiley and Sons, Inc

[B29] Bukovsky A Presl J Zidovsky J Mancal P The localization of Thy-1.1, MRC OX 2 and Ia antigens in the rat ovary and fallopian tube Immunology 1983 48 587 596 6131030 PMC1454039

[B30] Grim M Bukovsky A How closely are rat skeletal muscle development and regeneration processes related? Bibl Anat 1986 29 154 172 2873812

[B31] Serre G Vincent C Viraben R Soleilhavoup JP Natural IgM and IgG autoantibodies to epidermal keratins in normal human sera. I: ELISA-titration, immunofluorescence study J Invest Dermatol 1987 88 21 27 10.1111/1523-1747.ep12464810 2432133

[B32] Avrameas S Natural autoantibodies: from 'horror autotoxicus' to 'gnothi seauton' Immunol Today 1991 12 154 159 1715166 10.1016/S0167-5699(05)80045-3

[B33] Barrett TA Gajewski TF Danielpour D Chang EB Beagley KW Bluestone JA Differential function of intestinal intraepithelial lymphocyte subsets J Immunol 1992 149 1124 1130 1380032

[B34] Beagley KW Husband AJ Intraepithelial lymphocytes: origins, distribution, and function Crit Rev Immunol 1998 18 237 254 10.1615/CritRevImmunol.v18.i3.40 9637412

[B35] Yoon JW Jun HS Santamaria P Cellular and molecular mechanisms for the initiation and progression of beta cell destruction resulting from the collaboration between macrophages and T cells Autoimmunity 1998 27 109 122 10.3109/08916939809008041 9583742

[B36] Schranz DB Lernmark A Immunology in diabetes: an update Diabetes Metab Rev 1998 14 3 29 10.1002/(SICI)1099-0895(199803)14:1<3::AID-DMR206>3.0.CO;2-T 9605628

[B37] Bukovsky A Ayala ME Dominguez R Keenan JA Wimalasena J McKenzie PP Caudle MR Postnatal androgenization induces premature aging of rat ovaries Steroids 2000 65 190 205 10.1016/S0039-128X(99)00101-4 10713307

[B38] Bukovsky A Caudle MR Keenan JA Dominant role of monocytes in control of tissue function and aging Med Hypotheses 2000 55 337 347 10.1054/mehy.2000.1065 11000064

[B39] Havran WL Jameson JM Witherden DA Epithelial cells and their neighbors. III. Interactions between intraepithelial lymphocytes and neighboring epithelial cells Am J Physiol Gastrointest Liver Physiol 2005 289 G627 G630 16160077 10.1152/ajpgi.00224.2005

[B40] Komori HK Meehan TF Havran WL Epithelial and mucosal gammadelta T cells Curr Opin Immunol 2006 18 534 538 10.1016/j.coi.2006.06.001 16837181

[B41] Havran WL Jameson JM Epidermal T cells and wound healing J Immunol 2010 184 5423 5428 10.4049/jimmunol.0902733 20483798 PMC2944652

[B42] Macleod AS Havran WL Functions of skin-resident gammadelta T cells Cell Mol Life Sci 2011 68 2399 2408 10.1007/s00018-011-0702-x 21560071 PMC3123394

[B43] Carrel A Growth-promoting function of leukocytes J Exp Med 1922 36 385 391 10.1084/jem.36.4.385 19868680 PMC2128314

[B44] Fidler IJ Lymphocytes are not only immunocytes Biomedicine 1980 32 1 3 7370376

[B45] Yonish Rouach E Grunwald D Wilder S Kimchi A May E Lawrence JJ May P Oren M p53-mediated cell death: relationship to cell cycle control Mol Cell Biol 1993 13 1415 1423 8441387 10.1128/mcb.13.3.1415-1423.1993 PMC359451

[B46] Gonzalo JA Baixeras E Gonzalez-Garcia A George-Chandy A Van RN Martinez C Kroemer G Differential in vivo effects of a superantigen and an antibody targeted to the same T cell receptor. Activation-induced cell death vs passive macrophage-dependent deletion J Immunol 1994 152 1597 1608 8120373

[B47] Nargi JL Woodford-Thomas TA Cloning and characterization of a cdc25 phosphatase from mouse lymphocytes Immunogenetics 1994 39 99 108 8276463 10.1007/BF00188612

[B48] Yamamoto M Fujihashi K Amano M McGhee JR Beagley KW Kiyono H Cytokine synthesis and apoptosis by intestinal intraepithelial lymphocytes: signaling of high density alpha beta T cell receptor+ and gamma delta T cell receptor+ T cells via T cell receptor-CD3 complex results in interferon-gamma and interleukin-5 production, while low density T cells undergo DNA fragmentation Eur J Immunol 1994 24 1301 1306 10.1002/eji.1830240609 8206090

[B49] Reynisdottir I Polyak K Iavarone A Massague J Kip/Cip and Ink4 Cdk inhibitors cooperate to induce cell cycle arrest in response to TGF-beta Genes Dev 1995 9 1831 1845 10.1101/gad.9.15.1831 7649471

[B50] Jorres A Ludat K Lang J Sander K Gahl GM Frei U DeJonge K Williams JD Topley N Establishment and functional characterization of human peritoneal fibroblasts in culture: regulation of interleukin-6 production by proinflammatory cytokines J Am Soc Nephrol 1996 7 2192 2201 8915980 10.1681/ASN.V7102192

[B51] Saile B Eisenbach C El-Armouche H Neubauer K Ramadori G Antiapoptotic effect of interferon-alpha on hepatic stellate cells (HSC): a novel pathway of IFN-alpha signal transduction via Janus kinase 2 (JAK2) and caspase-8 Eur J Cell Biol 2003 82 31 41 10.1078/0171-9335-00285 12602946

[B52] Fischer AN Herrera B Mikula M Proell V Fuchs E Gotzmann J Schulte-Hermann R Beug H Mikulits W Integration of Ras subeffector signaling in TGF-beta mediated late stage hepatocarcinogenesis Carcinogenesis 2005 26 931 942 15705598 10.1093/carcin/bgi043

[B53] Li X Lu Y Huang W Xu H Chen X Geng Q Fan H Tan Y Xue G Jiang X In vitro effect of adenovirus-mediated human Gamma Interferon gene transfer into human mesenchymal stem cells for chronic myelogenous leukemia Hematol Oncol 2006 24 151 158 10.1002/hon.779 16700092

[B54] Campanaro S Picelli S Torregrossa R Colluto L Ceol M Del PD D'Angelo A Valle G Anglani F Genes involved in TGF beta1-driven epithelial-mesenchymal transition of renal epithelial cells are topologically related in the human interactome map BMC Genomics 2007 8 383 10.1186/1471-2164-8-383 17953753 PMC2174485

[B55] Fu X He Y Xie C Liu W Bone marrow mesenchymal stem cell transplantation improves ovarian function and structure in rats with chemotherapy-induced ovarian damage Cytotherapy 2008 10 353 363 10.1080/14653240802035926 18574768

[B56] Bierie B Moses HL Transforming growth factor beta (TGF-beta) and inflammation in cancer Cytokine Growth Factor Rev 2010 21 49 59 10.1016/j.cytogfr.2009.11.008 20018551 PMC2834863

[B57] Ramasamy R Tong CK Yip WK Vellasamy S Tan BC Seow HF Basic fibroblast growth factor modulates cell cycle of human umbilical cord-derived mesenchymal stem cells Cell Prolif 2012 45 132 139 10.1111/j.1365-2184.2012.00808.x 22309282 PMC6495492

[B58] Luo H Zhang Y Zhang Z Jin Y The protection of MSCs from apoptosis in nerve regeneration by TGFbeta1 through reducing inflammation and promoting VEGF-dependent angiogenesis Biomaterials 2012 33 4277 4287 10.1016/j.biomaterials.2012.02.042 22425554

[B59] Bukovsky A Presl J Krabec Z Bednarik T Ovarian function in adult rats treated with antithymocyte serum Experientia 1977 33 280 281 10.1007/BF02124112 300334

[B60] Bukovsky A Presl J Krabec Z Effects of postnatal progesterone treatment on ovarian function in adult rats Experientia 1979 35 562 563 10.1007/BF01922773 571352

[B61] Bukovsky A Presl J Holub M The role of the immune system in ovarian function control Allergol Immunopathol 1981 9 447 456 6758562

[B62] Bukovsky A Presl J Holub M Mancal P Krabec Z The localization of brain-thymus shared antigen (Thy-1) and thymosin 5 within the adult rat ovary IRCS Med Sci 1982 10 69 70

[B63] Bukovsky A Michael SD Presl J Cell-mediated and neural control of morphostasis Med Hypotheses 1991 36 261 268 10.1016/0306-9877(91)90146-P 1686295

[B64] Bukovsky A Caudle MR Keenan JA Wimalasena J Foster JS Van Meter SE Quantitative evaluation of the cell cycle-related retinoblastoma protein and localization of Thy-1 differentiation protein and macrophages during follicular development and atresia, and in human corpora lutea Biol Reprod 1995 52 776 792 10.1095/biolreprod52.4.776 7780000

[B65] Bukovsky A Caudle MR Keenan JA Motta PM Regulation of ovarian function by immune system components: the tissue control system (TCS) Microscopy of Reproduction and Development: A Dynamic Approach 1997 Roma: Antonio Delfino Editore 79 89

[B66] Bukovsky A Caudle MR Ekerdt DJ Immunology: animal models Encyclopedia of Aging 2002 New York: Macmillan Reference USA 691 695

[B67] Bukovsky A Ayala ME Dominguez R Keenan JA Wimalasena J Elder RF Caudle MR Changes of ovarian interstitial cell hormone receptors and behavior of resident mesenchymal cells in developing and adult rats with steroid-induced sterility Steroids 2002 67 277 289 10.1016/S0039-128X(01)00159-3 11856552

[B68] Bukovsky A Cell commitment by asymmetric division and immune system involvement Prog Mol Subcell Biol 2007 45 179 204 10.1007/978-3-540-69161-7_8 17585501

[B69] Bukovsky A Gupta SK Svetlikova M White RS Copas P Upadhyaya NB Van Meter SE Gonzalez-Bulnes A Immunoregulation of ovarian homeostasis Novel Concepts in Ovarian Endocrinology 2008 Kerala, India: Research Signpost 131 168

[B70] Bukovsky A Caudle MR Svetlikova M Steroid-mediated differentiation of neural/neuronal cells from epithelial ovarian precursors in vitro Cell Cycle 2008 7 3577 3583 10.4161/cc.7.22.7101 19001872

[B71] Bukovsky A Caudle MR Carson RJ Gaytan F Huleihel M Kruse A Schatten H Telleria CM Immune physiology in tissue regeneration and aging, tumor growth, and regenerative medicine Aging 2009 1 157 181 20195382 10.18632/aging.100024 PMC2830052

[B72] Bukovsky A Caudle MR Virant-Klun I Gupta SK Dominguez R Svetlikova M Xu F Immune physiology and oogenesis in fetal and adult humans, ovarian infertility, and totipotency of adult ovarian stem cells Birth Defects Res C Embryo Today 2009 87 64 89 10.1002/bdrc.20146 19306350

[B73] Hillier SG Zeleznik AJ Knazek RA Ross GT Hormonal regulation of preovulatory follicle maturation in the rat J Reprod Fertil 1980 60 219 229 10.1530/jrf.0.0600219 6776276

[B74] Dominguez R Zipitria D Aguilar L Riboni L Effects of unilateral destruction of the cervico-vaginal plexus on ovulation in the rat J Endocrinol 1981 91 483 486 10.1677/joe.0.0910483 7328371

[B75] Nakamura Y Kato H Terranova PF Abdominal vagotomy decreased the number of ova shed and serum progesterone levels on estrus in the cyclic hamster Endocrinol Jpn 1992 39 141 145 10.1507/endocrj1954.39.141 1606915

[B76] Chryssikopoulos A The relationship between the immune and endocrine systems Ann N Y Acad Sci 1997 816 83 93 10.1111/j.1749-6632.1997.tb52132.x 9238258

[B77] Vinatier D Dufour P Tordjeman-Rizzi N Prolongeau JF Depret-Moser S Monnier JC Immunological aspects of ovarian function: role of the cytokines Eur J Obstet Gynecol Reprod Biol 1995 63 155 168 10.1016/0301-2115(95)02227-9 8903772

[B78] Allen E Ovogenesis during sexual maturity Am J Anat 1923 31 439 481 10.1002/aja.1000310502

[B79] Pearl R Schoppe WF Studies on the physiology of reproduction in the domestic fowl. XVIII. Further observations on the anatomical basis of fecundity J Exp Zool 1921 34 101 189

[B80] Zuckerman S The number of oocytes in the mature ovary Recent Prog Horm Res 1951 6 63 109

[B81] Franchi LL Mandl AM Zuckerman S Zuckerman S The development of the ovary and the process of oogenesis The Ovary 1962 London: Academic Press 1 88

[B82] Zuckerman S Baker TG Zuckerman S, Weir BJ The development of the ovary and the process of oogenesis The Ovary, Volume I 1977 New York: Academic Press 41 67

[B83] Kingery HM Oogenesis in the white mouse J Morphol 1917 30 261 315 10.1002/jmor.1050300108

[B84] Gerard P Contribution a l'etude de l'ovarie des mammiferes. L'ovaire de *Galago mossambicus* (Young) Arch Biol 1920 43 357 391

[B85] Rao CRN On the structure of the ovary and the ovarian ovum of Loris lydekkerianus Cabr Qaurt J Micr Sci 1928 71 57 73

[B86] Zuckerman S Weir BJ The Ovary. Second Edition, Volume I 1977 New York: Academic Press

[B87] Bukovsky A Keenan JA Caudle MR Wimalasena J Upadhyaya NB Van Meter SE Immunohistochemical studies of the adult human ovary: possible contribution of immune and epithelial factors to folliculogenesis Am J Reprod Immunol 1995 33 323 340 7546251 10.1111/j.1600-0897.1995.tb00901.x

[B88] Bukovsky A Bukovsky A Fong CA Human oogenesis and follicular renewal from ovarian somatic stem cells Stem Cell Research Developments Bukovsky a 2007 Hauppauge, NY: Nova Science Publishers, Inc 229 272

[B89] Block E Quantitative morphological investigations of the follicular system in women. Variations at different ages Acta Anat (Basel) 1952 14 108 123 10.1159/000140595 14932631

[B90] Kerr JB Duckett R Myers M Britt KL Mladenovska T Findlay JK Quantification of healthy follicles in the neonatal and adult mouse ovary: evidence for maintenance of primordial follicle supply Reproduction 2006 132 95 109 10.1530/rep.1.01128 16816336

[B91] Johnson J Canning J Kaneko T Pru JK Tilly JL Germline stem cells and follicular renewal in the postnatal mammalian ovary Nature 2004 428 145 150 10.1038/nature02316 15014492

[B92] Ingram DL Zuckerman S Atresia The Ovary 1962 London: Academic Press 247 273

[B93] Gougeon A Echochard R Thalabard JC Age-related changes of the population of human ovarian follicles: increase in the disappearance rate of non-growing and early- growing follicles in aging women Biol Reprod 1994 50 653 663 10.1095/biolreprod50.3.653 8167237

[B94] Bukovsky A Virant-Klun I Simon C, Pellicer A Adult stem cells in the human ovary Stem Cells in Reproductive Medicine: Basic Science & Therapeutic Potential 2007 London: Informa Healthcare 53 69

[B95] Ioannou JM Oogenesis in adult prosimians J Embryol Exp Morphol 1968 17 139 145 6040545

[B96] van DM Polman JE De B,I van GK Bunschoten H Grootenhuis A Brindle J Aitken RJ Recombinant human zona pellucida protein ZP3 produced by chinese hamster ovary cells induces the human sperm acrosome reaction and promotes sperm-egg fusion Biol Reprod 1994 51 607 617 10.1095/biolreprod51.4.607 7819440

[B97] Ding J Rana N Dmowski WP Intracytoplasmic sperm injection into zona-free human oocytes results in normal fertilization and blastocyst development Hum Reprod 1999 14 476 478 10.1093/humrep/14.2.476 10099997

[B98] Balaban B Urman B Sertac A Alatas C Aksoy S Mercan R Oocyte morphology does not affect fertilization rate, embryo quality and implantation rate after intracytoplasmic sperm injection Hum Reprod 1998 13 3431 3433 10.1093/humrep/13.12.3431 9886529

[B99] Senger S Csokmay J Tanveer A Jones TI Sengupta P Lilly MA The nucleoporin Seh1 forms a complex with Mio and serves an essential tissue-specific function in Drosophila oogenesis Development 2011 138 2133 2142 10.1242/dev.057372 21521741 PMC3082312

[B100] Bukovsky A Caudle MR Svetlikova M Wimalasena J Ayala ME Dominguez R Oogenesis in adult mammals, including humans: a review Endocrine 2005 26 301 316 10.1385/ENDO:26:3:301 16034186

[B101] Allen E Creadick RN Ovogenesis during sexual maturity, the first stage, mitosis in the germinal epithelium, as shown by the colchicine technique Anat Rec 1937 69 191 195 10.1002/ar.1090690209

[B102] Zou K Yuan Z Yang Z Luo H Sun K Zhou L Xiang J Shi L Yu Q Zhang Y Production of offspring from a germline stem cell line derived from neonatal ovaries Nat Cell Biol 2009 11 631 636 10.1038/ncb1869 19363485

[B103] White YA Woods DC Takai Y Ishihara O Seki H Tilly JL Oocyte formation by mitotically active germ cells purified from ovaries of reproductive-age women Nat Med 2012 18 413 421 10.1038/nm.2669 22366948 PMC3296965

[B104] Baas T Repowering the ovary. Science-Business eXchange 2012 5 4 6

[B105] Clark AT Bodnar MS Fox M Rodriquez RT Abeyta MJ Firpo MT Pera RA Spontaneous differentiation of germ cells from human embryonic stem cells in vitro Hum Mol Genet 2004 13 727 739 10.1093/hmg/ddh088 14962983

[B106] Cauffman G Van d V Liebaers I Van SA DAZL expression in human oocytes, preimplantation embryos and embryonic stem cells Mol Hum Reprod 2005 11 405 411 10.1093/molehr/gah167 15879466

[B107] Alberts B Johnson A Lewis J Raff M Roberts K Walter P Molecular Biology of the Cell 2002 New York: Garland Science

[B108] Kelly SJ Studies of the developmental potential of 4- and 8-cell stage mouse blastomeres J Exp Zool 1977 200 365 376 10.1002/jez.1402000307 559722

[B109] Ginsburg M Snow MH McLaren A Primordial germ cells in the mouse embryo during gastrulation Development 1990 110 521 528 2133553 10.1242/dev.110.2.521

[B110] Lawson KA Hage WJ Clonal analysis of the origin of primordial germ cells in the mouse Ciba Found Symp 1994 182 68 84 7835158 10.1002/9780470514573.ch5

[B111] Tam PP Zhou SX The allocation of epiblast cells to ectodermal and germ-line lineages is influenced by the position of the cells in the gastrulating mouse embryo Dev Biol 1996 178 124 132 10.1006/dbio.1996.0203 8812114

[B112] Peters H McNatty KP The Ovary. A Correlation of Structure and Function in Mammals 1980 Berkeley and Los Angeles, California: University of California Press

[B113] Castrillon DH Quade BJ Wang TY Quigley C Crum CP The human VASA gene is specifically expressed in the germ cell lineage Proc Natl Acad Sci U S A 2000 97 9585 9590 10.1073/pnas.160274797 10920202 PMC16908

[B114] Simkins CS Development of the human ovary from birth to sexual maturity J Anat 1932 51 465 505 10.1002/aja.1000510208

[B115] Motta PM Makabe S Development of the ovarian surface and associated germ cells in the human fetus Cell Tissue Res 1982 226 493 510 7139687 10.1007/BF00214779

[B116] Motta PM Makabe S Germ cells in the ovarian surface during fetal development in humans. A three-dimensional microanatomical study by scanning and transmission electron microscopy J Submicrosc Cytol 1986 18 271 290 3712511

[B117] Olweus J BitMansour A Warnke R Thompson PA Carballido J Picker LJ Lund-Johansen F Dendritic cell ontogeny: a human dendritic cell lineage of myeloid origin Proc Natl Acad Sci U S A 1997 94 12551 12556 10.1073/pnas.94.23.12551 9356487 PMC25034

[B118] Byskov AG Skakkebaek NE Stafanger G Peters H Influence of ovarian surface epithelium and rete ovarii on follicle formation J Anat 1977 123 77 86 838624 PMC1234254

[B119] Van Wagenen G Simpson ME Embryology of the ovary and testis Homo sapiens and Macaca mulatta 1965 New Haven: Yale University Press

[B120] Everett NB Observational and experimental evidences relating to the origin and differentiation of the definite germ cells in mice J Exp Zool 1943 92 49 91 10.1002/jez.1400920104

[B121] Brambell FWR The development and morphology of the gonads of the mouse. Part 1. The morphogenesis of the indifferent gonad and of the ovary Proc Roy Soc 1927 101 391 409 10.1098/rspb.1927.0022

[B122] Motta PM Van Blerkom J Makabe S Changes in the surface morphology of ovarian 'germinal' epithelium during the reproductive cycle and in some pathological conditions J Submicrosc Cytol 1980 12 407 425

[B123] Sawyer HR Smith P Heath DA Juengel JL Wakefield SJ McNatty KP Formation of ovarian follicles during fetal development in sheep Biol Reprod 2002 66 1134 1150 10.1095/biolreprod66.4.1134 11906935

[B124] Bousfield GR Butnev VY Gotschall RR Baker VL Moore WT Structural features of mammalian gonadotropins Mol Cell Endocrinol 1996 125 3 19 10.1016/S0303-7207(96)03945-7 9027339

[B125] Bukovsky A Mesenchymal cells in tissue homeostasis and cancer Mod Asp Immunobiol 2000 1 43 47

[B126] Bukovsky A Caudle MR Keenan JA Upadhyaya NB Van Meter S Wimalasena J Elder RF Association of mesenchymal cells and immunoglobulins with differentiating epithelial cells BMC Dev Biol 2001 1 11 http://www.biomedcentral.com/1471-213X/1/11 10.1186/1471-213X-1-11 11439174 PMC34117

[B127] Swift CH Origin and early history of the primordial germ-cells of the chick Am J Anat 1914 15 483 516 10.1002/aja.1000150404

[B128] Reagan FP Some results and possibilities of early embryonic castration Anat Rec 1916 11 251 267 10.1002/ar.1090110507

[B129] Lillie FR The development of the chick 1908 New York: Henry Holt & Co

[B130] Baker TG Balin H, Glasser S Oogenesis and ovarian development Reproductive Biology 1972 Amsterdam: Excerpta Medica 398 437

[B131] Yao HH DiNapoli L Capel B Meiotic germ cells antagonize mesonephric cell migration and testis cord formation in mouse gonads Development 2003 130 5895 5902 10.1242/dev.00836 14561636 PMC4073601

[B132] Bukovsky A Oogenesis from human somatic stem cells and a role of immune adaptation in premature ovarian failure Curr Stem Cell Res Ther 2006 1 289 303 18220874 10.2174/157488806778226795

[B133] Auersperg N Wong AS Choi KC Kang SK Leung PC Ovarian surface epithelium: biology, endocrinology, and pathology Endocr Rev 2001 22 255 288 10.1210/er.22.2.255 11294827

[B134] Bukovsky A Immune system involvement in the regulation of ovarian function and augmentation of cancer Microsc Res Tech 2006 69 482 500 10.1002/jemt.20307 16703613

[B135] Bukovsky A Ayala ME Dominguez R Svetlikova M Selleck-White R Bone marrow derived cells and alternative pathways of oogenesis in adult rodents Cell Cycle 2007 6 2306 2309 10.4161/cc.6.18.4707 17890900

[B136] Motta PM Makabe S Naguro T Correr S Oocyte follicle cells association during development of human ovarian follicle. A study by high resolution scanning and transmission electron microscopy. Arch Histol Cytol 1994 57 369 394 10.1679/aohc.57.369 7880591

[B137] Liu Y Wu C Lyu Q Yang D Albertini DF Keefe DL Liu L Germline stem cells and neo-oogenesis in the adult human ovary Dev Biol 2007 306 112 120 10.1016/j.ydbio.2007.03.006 17428461

[B138] Tilly JL Johnson J Recent arguments against germ cell renewal in the adult human ovary: Is an absence of marker gene expression really acceptable evidence of an absence of oogenesis? Cell Cycle 2007 6 879 883 10.4161/cc.6.8.4185 17438374

[B139] Bukovsky A Caudle MR Gupta SK Svetlikova M Selleck-White R Ayala ME Dominguez R Mammalian neo-oogenesis and expression of meiosis-specific protein SCP3 in adult human and monkey ovaries Cell Cycle 2008 7 683 686 10.4161/cc.7.5.5453 18256545

[B140] Tres LL XY chromosomal bivalent: nucleolar attraction Mol Reprod Dev 2005 72 1 6 10.1002/mrd.20334 15915516

[B141] Faddy MJ Follicle dynamics during ovarian ageing Mol Cell Endocrinol 2000 163 43 48 10.1016/S0303-7207(99)00238-5 10963872

[B142] Niikura Y Niikura T Tilly JL Aged mouse ovaries possess rare premeiotic germ cells that can generate oocytes following transplantation into a young host environment Aging (Albany NY) 2009 1 971 978 20157580 10.18632/aging.100105 PMC2815754

[B143] Kay MM An overview of immune aging Mech Ageing Dev 1979 9 39 59 10.1016/0047-6374(79)90119-2 155761

[B144] Talbert GB Effect of maternal age on reproductive capacity Am J Obstet Gynecol 1968 102 451 477 4877579 10.1016/0002-9378(68)90019-7

[B145] Kirkwood TB Ovarian ageing and the general biology of senescence Maturitas 1998 30 105 111 10.1016/S0378-5122(98)00065-6 9871904

[B146] Balfour BM Drexhage HA Kamperdijk EW Hoefsmit EC Antigen-presenting cells, including Langerhans cells, veiled cells and interdigitating cells Ciba Found Symp 1981 84 281 301 7023875 10.1002/9780470720660.ch15

[B147] Hoefsmit EC Duijvestijn AM Kamperdijk EW Relation between Langerhans cells, veiled cells, and interdigitating cells Immunobiology 1982 161 255 265 10.1016/S0171-2985(82)80081-8 7047373

[B148] Knight SC Farrant J Bryant A Edwards AJ Burman S Lever A Clarke J Webster AD Non-adherent, low-density cells from human peripheral blood contain dendritic cells and monocytes, both with veiled morphology Immunology 1986 57 595 603 3007336 PMC1453865

[B149] Howard CJ Hope JC Dendritic cells, implications on function from studies of the afferent lymph veiled cell Vet Immunol Immunopathol 2000 77 1 13 10.1016/S0165-2427(00)00234-8 11068062

[B150] Mathe G Immunity aging. I. The chronic perduration of the thymus acute involution at puberty? Or the participation of the lymphoid organs and cells in fatal physiologic decline? Biomed Pharmacother 1997 51 49 57 10.1016/S0753-3322(97)87726-8 9161467

[B151] Rebar RW The thymus gland and reproduction: do thymic peptides influence the reproductive lifespan in females? J Am Geriatr Soc 1982 30 603 606 7050222 10.1111/j.1532-5415.1982.tb05672.x

[B152] Suh BY Naylor PH Goldstein AL Rebar RW Modulation of thymosin beta 4 by estrogen Am J Obstet Gynecol 1985 151 544 549 2983555 10.1016/0002-9378(85)90286-8

[B153] Lintern Moore S Pantelouris EM Ovarian development in athymic nude mice. The size and composition of the follicle population Mech Ageing Dev 1975 4 385 390 1228336 10.1016/0047-6374(75)90039-1

[B154] Lo PA Ruvolo G Gancitano RA Cittadini E Ovarian function following radiation and chemotherapy for cancer Eur J Obstet Gynecol Reprod Biol 2004 113 Suppl 1 S33 S40 15041128 10.1016/j.ejogrb.2003.11.008

[B155] Hoek A van Kasteren Y de Haan-Meulman M Schoemaker J Drexhage HA Dysfunction of monocytes and dendritic cells in patients with premature ovarian failure Am J Reprod Immunol 1993 30 207 217 8129847 10.1111/j.1600-0897.1993.tb00622.x

[B156] Hoek A van Kasteren Y de Haan-Meulman M Hooijkaas H Schoemaker J Drexhage HA Analysis of peripheral blood lymphocyte subsets, NK cells, and delayed type hypersensitivity skin test in patients with premature ovarian failure Am J Reprod Immunol 1995 33 495 502 7576124 10.1111/j.1600-0897.1995.tb00912.x

[B157] Rebar RW Lobo RA, Kesley J, Marcus R Premature ovarian failure Menopause Biology and Pathobiology 2000 San Diego: Academic Press 135 146

[B158] Edassery SL Shatavi SV Kunkel JP Hauer C Brucker C Penumatsa K Yu Y Dias JA Luborsky JL Autoantigens in ovarian autoimmunity associated with unexplained infertility and premature ovarian failure Fertil Steril 2010 94 2636 2641 10.1016/j.fertnstert.2010.04.012 20522323 PMC2948062

[B159] Kumar M Pathak D Venkatesh S Kriplani A Ammini AC Dada R Chromosomal abnormalities & oxidative stress in women with premature ovarian failure (POF) Indian J Med Res 2012 135 92 97 10.4103/0971-5916.93430 22382189 PMC3307192

[B160] Bukovsky A Caudle MR Keenan JA Wimalasena J Upadhyaya NB Van Meter SE Is corpus luteum regression an immune-mediated event? Localization of immune system components, and luteinizing hormone receptor in human corpora lutea Biol Reprod 1995 53 1373 1384 10.1095/biolreprod53.6.1373 8562694

[B161] Nagasawa H Yanai R Kikuyama S Mori J Pituitary secretion of prolactin, luteinizing hormone and follicle-stimulating hormone in adult female rats treated neonatally with oestrogen J Endocrinol 1973 59 599 604 10.1677/joe.0.0590599 4796894

[B162] Matsumoto A Asai T Wakabayashi K Effects of x-ray irradiation on the subsequent gonadotropin secretion in normal and neonatally estrogenized female rats Endocrinol Jpn 1975 22 233 241 10.1507/endocrj1954.22.233 1175524

[B163] Deshpande RR Chapman JC Michael SD The anovulation in female mice resulting from postnatal injections of estrogen is correlated with altered levels of CD8+ lymphocytes Am J Reprod Immunol 1997 38 114 120 10.1111/j.1600-0897.1997.tb00285.x 9272210

[B164] Swanson HE van der Werff t Bosch JJ The "early-androgen" syndrome; differences in response to prenatal and postnatal administration of various doses of testosterone propionate in female and male rats Acta Endocrinol (Copenh) 1964 47 37 50 14208150

[B165] Coghlan A Doubts cast over 'eggs on tap' New Scientist 2005 2499 13

[B166] Nussbaum M Zur Differenzierung des Geschlechts im Terreich Arch mikrosk Anat EntwMech 1880 18 121 131

[B167] Telfer EE Germline stem cells in the postnatal mammalian ovary: A phenomenon of prosimian primates and mice? Reprod Biol Endocrinol 2004 2 24 10.1186/1477-7827-2-24 15149546 PMC434530

[B168] Gougeon A Is neo-oogenesis in the adult ovary, a realistic paradigm? Gynecol Obstet Fertil 2010 38 398 401 10.1016/j.gyobfe.2010.04.013 20576550

[B169] Parte SC Bhartiya D Telang J Daithankar VV Salvi V Zaveri K Hinduja I Detection Characterization and Spontaneous Differentiation in vitro of Very Small Embryonic-like Putative Stem Cells in Adult Mammalian Ovary 2011 Stem Cells Dev 10.1089/scd.2010.0461 PMC3148829 21291304

[B170] Johnson J Bagley J Skaznik-Wikiel M Lee HJ Adams GB Niikura Y Tschudy KS Tilly JC Cortes ML Forkert R Oocyte generation in adult mammalian ovaries by putative germ cells in bone marrow and peripheral blood Cell 2005 122 303 315 10.1016/j.cell.2005.06.031 16051153 PMC11771209

[B171] Kirby ML Bockman DE Neural crest and normal development: a new perspective Anat Rec 1984 209 1 6 10.1002/ar.1092090102 6731866

[B172] Bockman DE Kirby ML Dependence of thymus development on derivatives of the neural crest Science 1984 223 498 500 10.1126/science.6606851 6606851

[B173] McNatty KP Heath DA Henderson KM Lun S Hurst PR Ellis LM Montgomery GW Morrison L Thurley DC Some aspects of thecal and granulosa cell function during follicular development in the bovine ovary J Reprod Fertil 1984 72 39 53 10.1530/jrf.0.0720039 6540808

[B174] Morley P Armstrong DT Calaresu FR Ovarian nerve extracts influence androgen production by cultured ovarian thecal cells Neuroendocrinology 1989 50 93 99 10.1159/000125207 2547178

[B175] Morley P Armstrong DT Calaresu FR Site at which ovarian nerve extracts inhibit thecal androgen production Mol Cell Endocrinol 1990 71 33 40 10.1016/0303-7207(90)90072-G 2163932

[B176] Bukovsky A Caudle MR Keenan JA Wimalasena J Upadhyaya NB Van Meter SE Is irregular regression of corpora lutea in climacteric women caused by age-induced alterations in the "tissue control system"? Am J Reprod Immunol 1996 36 327 341 10.1111/j.1600-0897.1996.tb00183.x 8985508

[B177] Mossman HW Duke KL Greep RO Some comparative aspects of the mammalian ovary Handbook of Physiology, Sect. 7: Endocrinology 1973 Washington: Am. Physiol. Soc 389 402

[B178] Bukovsky A Origin of germ cells and follicular renewal in adult human ovaries. Presented at microscopy & microanalysis conference 2005 - July 31 - august 4, Honolulu, Hawaii (invited) 2005 (Abstract)

[B179] Bukovsky A Ovarian stem cells and mammalian neo-oogenesis Microsc Microanal 2008 14 Suppl 2 1474 1475

[B180] McNatty KP Makris A DeGrazia C Osathanondh R Ryan KJ The production of progesterone, androgens, and estrogens by granulosa cells, thecal tissue, and stromal tissue from human ovaries in vitro J Clin Endocrinol Metab 1979 49 687 699 10.1210/jcem-49-5-687 489711

[B181] McNatty KP Makris A Osathanondh R Ryan KJ Effects of luteinizing hormone on steroidogenesis by thecal tissue from human ovarian follicles in vitro Steroids 1980 36 53 63 10.1016/0039-128X(80)90067-7 7414656

[B182] Nelson LR Bulun SE Estrogen production and action J Am Acad Dermatol 2001 45 S116 S124 10.1067/mjd.2001.117432 11511861

[B183] Blaustein A Kaganowicz A Wells J Tumor markers in inclusion cysts of the ovary Cancer 1982 49 722 726 10.1002/1097-0142(19820215)49:4<722::AID-CNCR2820490421>3.0.CO;2-C 7034921

[B184] Martinez ML Fontenot GK Harris JD The expression and localization of zona pellucida glycoproteins and mRNA in cynomolgus monkeys (*Macaca fascicularis*) J Reprod Fertil Suppl 1996 50 35 41 8984186

[B185] Prasad SV Skinner SM Carino C Wang N Cartwright J Dunbar BS Structure and function of the proteins of the mammalian Zona pellucida Cells Tissues Organs 2000 166 148 164 10.1159/000016730 10729725

[B186] Santos TA Dias C Henriques P Brito R Barbosa A Regateiro F Santos AA Cytogenetic analysis of spontaneously activated noninseminated oocytes and parthenogenetically activated failed fertilized human oocytes–implications for the use of primate parthenotes for stem cell production J Assist Reprod Genet 2003 20 122 130 10.1023/A:1022630924236 12735388 PMC3455586

[B187] Pepling ME Spradling AC Female mouse germ cells form synchronously dividing cysts Development 1998 125 3323 3328 9693136 10.1242/dev.125.17.3323

[B188] Pepling ME Spradling AC Mouse ovarian germ cell cysts undergo programmed breakdown to form primordial follicles Dev Biol 2001 234 339 351 10.1006/dbio.2001.0269 11397004

[B189] Virant-Klun I Zech N Rozman P Vogler A Cvjeticanin B Klemenc P Malicev E Meden-Vrtovec H Putative stem cells with an embryonic character isolated from the ovarian surface epithelium of women with no naturally present follicles and oocytes Differentiation 2008 76 8 843 856 10.1111/j.1432-0436.2008.00268.x 18452550

[B190] Virant-Klun I Rozman P Cvjeticanin B Vrtacnik-Bokal E Novakovic S Ruelicke T Parthenogenetic Embryo-Like Structures in the Human Ovarian Surface Epithelium Cell Culture in Postmenopausal Women with No Naturally Present Follicles and Oocytes Stem Cells Dev 2009 18 1 137 150 10.1089/scd.2007.0238 18605894

[B191] Virant-Klun I Skutella T Stem cells in aged mammalian ovaries Aging (Albany NY) 2010 2 3 6 20228938 10.18632/aging.100117 PMC2837201

[B192] Bukovsky A How Can Female Germline Stem Cells Contribute to the Physiological Neo-Oogenesis in Mammals and Why Menopause Occurs? Microsc Microanal 2011 17 498 505 20633318 10.1017/S143192761000036X

[B193] Gosden RG Transplantation of fetal germ cells J Assist Reprod Genet 1992 9 118 123 10.1007/BF01203750 1627926

[B194] Faddy MJ Gosden RG Gougeon A Richardson SJ Nelson JF Accelerated disappearance of ovarian follicles in mid-life: implications for forecasting menopause Hum Reprod 1992 7 1342 1346 1291557 10.1093/oxfordjournals.humrep.a137570

[B195] Nandedkar T Narkar M Stem cell research: its relevance to reproductive biology Indian J Exp Biol 2003 41 724 739 15255375

[B196] Oatley J Hunt PA Of mice and (wo)men: purified oogonial stem cells from mouse and human ovaries Biol Reprod 2012 86 196 10.1095/biolreprod.112.100297 22402962 PMC6322432

[B197] Tilly JL Telfer EE Purification of germline stem cells from adult mammalian ovaries: a step closer towards control of the female biological clock? Mol Hum Reprod 2009 15 393 398 10.1093/molehr/gap036 19509111 PMC2696346

[B198] Bukovsky A Gupta SK Bansal P Chakravarthy S Chaudhary M Svetlikova M White RS Copas P Upadhyaya NB Van Meter SE Production of monoclonal antibodies against recombinant human zona pellucida glycoproteins: utility in immunolocalization of respective zona proteins in ovarian follicles J Reprod Immunol 2008 78 102 114 10.1016/j.jri.2007.10.004 18313762

[B199] Virant-Klun I Skutella T Stimpfel M Sinkovec J Ovarian surface epithelium in patients with severe ovarian infertility: a potential source of cells expressing markers of pluripotent/multipotent stem cells J Biomed Biotechnol 2011 2011 381928 22187524 10.1155/2011/381928 PMC3237017

[B200] Bukovsky A Follicular renewal and age-related changes in ovaries NIH/NIA Grant Application: 1 R01 AG028003-01 2005 27 59

[B201] Luke B Brown MB Wantman E Lederman A Gibbons W Schattman GL Lobo RA Leach RE Stern JE Cumulative birth rates with linked assisted reproductive technology cycles N Engl J Med 2012 366 2483 2491 10.1056/NEJMoa1110238 22738098 PMC3623697

[B202] Bukovsky A Virant-Klun I Svetlikova M Willson I Ovarian germ cells Methods Enzymol 2006 419 208 258 17141058 10.1016/S0076-6879(06)19010-2

[B203] Bukovsky A Caudle MR Immune physiology of the mammalian ovary - a review Am J Reprod Immunol 2008 59 12 26 18154592 10.1111/j.1600-0897.2007.00562.x

[B204] Lee HJ Selesniemi K Niikura Y Niikura T Klein R Dombkowski DM Tilly JL Bone marrow transplantation generates immature oocytes and rescues long-term fertility in a preclinical mouse model of chemotherapy-induced premature ovarian failure J Clin Oncol 2007 25 3198 3204 10.1200/JCO.2006.10.3028 17664466

[B205] Van Blerkom J Motta PM The Cellular Basis of Mammalian Reproduction 1979 Baltimore-Munich: Urban & Schwarzenberg

[B206] Olszanecka A Posnik-Urbanska A Kawecka-Jaszcz K Czarnecka D Subclinical organ damage in perimenopausal women with essential hypertension Pol Arch Med Wewn 2010 120 390 398 20980944

[B207] Cuzick J Glasier A La Vecchia C Maraganore DM Negri E Rossi M Spector T Trichopoulos D van Baak MA Zocchetti C Perimenopausal risk factors and future health Hum Reprod Update 2011 17 706 717 21565809 10.1093/humupd/dmr020

[B208] Corrigan EC Raygada MJ Vanderhoof VH Nelson LM A woman with spontaneous premature ovarian failure gives birth to a child with fragile X syndrome Fertil Steril 2005 84 1508 16275254 10.1016/j.fertnstert.2005.06.019

[B209] Gersak K Meden-Vrtovec H Peterlin B Fragile X premutation in women with sporadic premature ovarian failure in Slovenia Hum Reprod 2003 18 1637 1640 10.1093/humrep/deg327 12871874

